# The Q-junction and the inflammatory response are critical pathological and therapeutic factors in CoQ deficiency

**DOI:** 10.1016/j.redox.2022.102403

**Published:** 2022-07-15

**Authors:** Pilar González-García, María Elena Díaz-Casado, Agustín Hidalgo-Gutiérrez, Laura Jiménez-Sánchez, Mohammed Bakkali, Eliana Barriocanal-Casado, Germaine Escames, Riccardo Zenezini Chiozzi, Franziska Völlmy, Esther A. Zaal, Celia R. Berkers, Albert J.R. Heck, Luis C. López

**Affiliations:** a Departamento de Fisiología, Facultad de Medicina, Universidad de Granada, 18016, Granada, Spain; b Instituto de Biotecnología, Centro de Investigación Biomédica, Universidad de Granada, 18016, Granada, Spain; c Ibs.Granada, 18016, Granada, Spain; d Departamento de Genética, Facultad de Ciencias, Universidad de Granada, 18071, Granada, Spain; e GENYO, Centre for Genomics and Oncological Research, Genomic Medicine Department, Pfizer-University of Granada-Andalusian Regional Government, 18016, Granada, Spain; f Biomolecular Mass Spectrometry and Proteomics, Bijvoet Center for Biomolecular Research and Utrecht Institute of Pharmaceutical Sciences, Utrecht University, 3584CH, Utrecht, Netherlands; g Netherlands Proteomics Centre, Padualaan 8, 3584 CH, Utrecht, the Netherlands; h Division of Cell Biology, Metabolism & Cancer, Department of Biomolecular Health Sciences, Faculty of Veterinary Medicine, Utrecht University, 3508 TD, Utrecht, the Netherlands

**Keywords:** Coenzyme Q, Mitochondrial disease, Therapy, Omics, Phenolic compound, CoQ, Coenzyme Q, VA, vanillic acid, β-RA, β-resorcylic acid, 4-HB, 4-hydroxybenzoic acid, 2,4-diHB, 2,4-dihydroxybenzoic acid, DMQ, demethoxyubiquinone, CoQ_9_, Coenzyme Q9, DMQ_9_, demethoxyubiquinone-9, MEFs, mouse embryonic fibroblasts, IL, interleukin, CHDH, choline dehydrogenase, CMC4, Cx_9_C motif-containing protein 4, COL1A1, collagen a-1 chain, ETFDH, electron transfer flavoprotein dehydrogenase, GPDH, glycerol-3-phosphate dehydrogenase, MASP1, mannose-binding lectin-associated serine protease-1, N-Ac-Glu, N-Acetyl-Glucosamine, N-Ac-Glu-6P, N-Acetyl-Glucosamine-6-Phosphate, PRODH, proline dehydrogenase, PZP, pregnancy zone protein, SQOR, sulfide quinone oxidoreductase, 4-HPP, 4-Hydroxyphenylpyruvate, DHODH, dihydroorotate dehydrogenase

## Abstract

Defects in Coenzyme Q (CoQ) metabolism have been associated with primary mitochondrial disorders, neurodegenerative diseases and metabolic conditions. The consequences of CoQ deficiency have not been fully addressed, and effective treatment remains challenging. Here, we use mice with primary CoQ deficiency (*Coq9*^*R239X*^), and we demonstrate that CoQ deficiency profoundly alters the Q-junction, leading to extensive changes in the mitochondrial proteome and metabolism in the kidneys and, to a lesser extent, in the brain. CoQ deficiency also induces reactive gliosis, which mediates a neuroinflammatory response, both of which lead to an encephalopathic phenotype. Importantly, treatment with either vanillic acid (VA) or β-resorcylic acid (β-RA), two analogs of the natural precursor for CoQ biosynthesis, partially restores CoQ metabolism, particularly in the kidneys, and induces profound normalization of the mitochondrial proteome and metabolism, ultimately leading to reductions in gliosis, neuroinflammation and spongiosis and, consequently, reversing the phenotype. Together, these results provide key mechanistic insights into defects in CoQ metabolism and identify potential disease biomarkers. Furthermore, our findings clearly indicate that the use of analogs of the CoQ biosynthetic precursor is a promising alternative therapy for primary CoQ deficiency and has potential for use in the treatment of more common neurodegenerative and metabolic diseases that are associated with secondary CoQ deficiency.

## Highlights

• Coenzyme Q deficiency induces the Q-junction alteration, which leads to profound metabolic consequences.
• Coenzyme Q deficiency also induces gliosis, neuroinflammation and spongiosis.
• β-resorcylic acid or vanillic acid, two analogs of the precursor for CoQ biosynthesis, partially restores the Q-junction.
• As a consequence, both compounds normalize mitochondrial metabolism and reduce gliosis, neuroinflammation and spongiosis.
• These effects lead to the rescue of the phenotype.

## Graphical abstract

**Figure:**
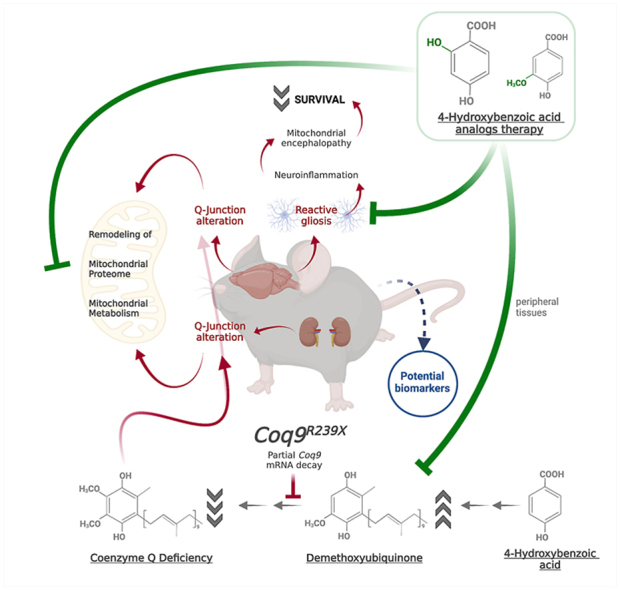

## 1 Introduction

Mitochondrial dysfunction is a common feature of neurodegenerative diseases, including Parkinson disease, Alzheimer disease and multiple system atrophy [1]. In fact, multiple system atrophy has been associated with some genetic variants of a gene involved in Coenzyme Q (CoQ) biosynthesis [2]. CoQ is mainly synthesized and localized in mitochondria, where it forms the Q-junction that links multiple metabolic pathways to ATP synthesis [3,4]. Primary CoQ deficiency causes a mitochondrial syndrome with varied clinical phenotypes [5,6]. Five major phenotypes have been described: 1) encephalomyopathy (recurrent myoglobinuria, encephalopathy, and mitochondrial myopathy); 2) cerebellar ataxia (cerebellar atrophy associated with other neurologic manifestations and, occasionally, endocrine dysfunctions); 3) infantile multisystemic form; 4) isolated myopathy, characterized by muscle weakness, myoglobinuria, exercise intolerance, and elevated creatine kinase (CK); and 5) nephropathy. Growth retardation, deafness, hearing loss, and cardiomyopathy have also been described in CoQ_10_-deficient patients [5]. This heterogeneity has been partially explained by 1) the association of the phenotype to mutations in specific genes of the CoQ biosynthetic pathway [5,6]; 2) the tissue specificities in the stability of the protein complex for CoQ biosynthesis (Complex Q) and the residual levels of CoQ; 3) the presence of intermediate metabolites of the CoQ biosynthetic pathway; 4) the disruption of the sulfide metabolism; 5) the increased oxidative stress; or 6) the decline in ATP synthesis [3]. However, additional mechanisms may exist, considering the multiple metabolic roles of CoQ [3,7].

The conventional treatment for CoQ deficiency is oral supplementation with high doses of CoQ_10_. However, therapeutic outcomes are heterogeneous, and some patients have a poor response [5,6]. The lack of efficacy of CoQ_10_ therapy is due to its modest absorption and bioavailability, which limits the amount of exogenous CoQ_10_ that reaches the mitochondria within the cells [8,9]. To address this limitation, an increase in the endogenous CoQ biosynthesis was proposed as an alternative strategy to increase the levels of CoQ in mitochondria. With this aim, analogs of 4-hydroxybenzoic acid (4-HB), the precursor of the CoQ biosynthetic pathway, have been rationally tested in different models of CoQ deficiency. Specifically, 2,4-dihydroxybenzoic acid (2,4-diHB), also named β-resorcylic acid (β-RA), has a hydroxyl group that is normally added to the benzoquinone ring in a reaction catalyzed by the hydroxylase COQ7 in the CoQ biosynthetic pathway; therefore, β-RA could bypass a defect in COQ7 (Fig. S1). Consistent with this, β-RA supplementation increased the levels of CoQ in *COQ7* null yeast and human skin fibroblasts with mutations in *COQ7* [[10], [11], [12]]. Since cells with defects in COQ7 accumulate the substrate of the reaction, demethoxyubiquinone (DMQ), β-RA treatment also decreased DMQ levels, supporting this bypass mechanism [12]. Moreover, COQ7 needs another protein, COQ9, for its stability and function (Fig. S1), and as a result, defects in COQ9 lead to CoQ deficiency and accumulation of DMQ [13]. Consequently, β-RA supplementation increased CoQ levels and decreased DMQ levels in cells with COQ9 defects [12,14]. *In vivo*, oral β-RA supplementation decreased the DMQ/CoQ ratio in different tissues of *Coq7* conditional knockout (aog*Coq7*) and *Coq9*^*R239X*^ mice, leading to marked rescue of the phenotype in both models [15,16]. However, β-RA did not modify the DMQ/CoQ ratio or the mitochondrial bioenergetics in the brain of the encephalopathic *Coq9*^*R239X*^ mice, even though it reduced astrogliosis and spongiosis [15], suggesting that β-RA may act through additional therapeutic mechanisms. In support of this possibility, β-RA rescued the renal phenotype of podocyte-specific conditional knockouts of *Coq6* and *Adck4* (*Coq6*^*podKO*^ and *Adck4*^*ΔPodocyte*^), but the therapeutic mechanisms were not identified and must not be related to a bypass effect [17,18], suggesting that β-RA could also act through CoQ-independent therapeutic mechanisms. Other 4-HB analogs, such as 3,4-hydroxybenzoic acid (3,4-diHB) and vanillic acid (VA), were able to bypass defects in COQ6, a monooxygenase required for the hydroxylation of the C5 carbon of the ring, in yeast and human cells (Fig. S1) [[19], [20], [21]]. Surprisingly, VA strongly upregulated COQ4 and stimulated CoQ biosynthesis in cells with a mutation in *COQ9*, leading to an increase in cell viability [12]. However, VA has not been tested *in vivo* in a mammalian model of CoQ deficiency.

Here, we show that VA rescues the phenotype of *Coq9*^*R239X*^ mice. Moreover, our multiomics analysis reveals that VA and β-RA act in the Q-junction, correcting perturbations of the mitochondrial proteome and metabolism. In addition, both treatments decreased neuroinflammation, reducing gliosis and spongiosis, which ultimately rescued the phenotype.

## 2 Results

### 2.1 VA supplementation rescues the phenotype of *Coq9*^*R239X*^ mice and induces a remarkable improvement in the histopathological signs of encephalopathy

**Fig. 1:**
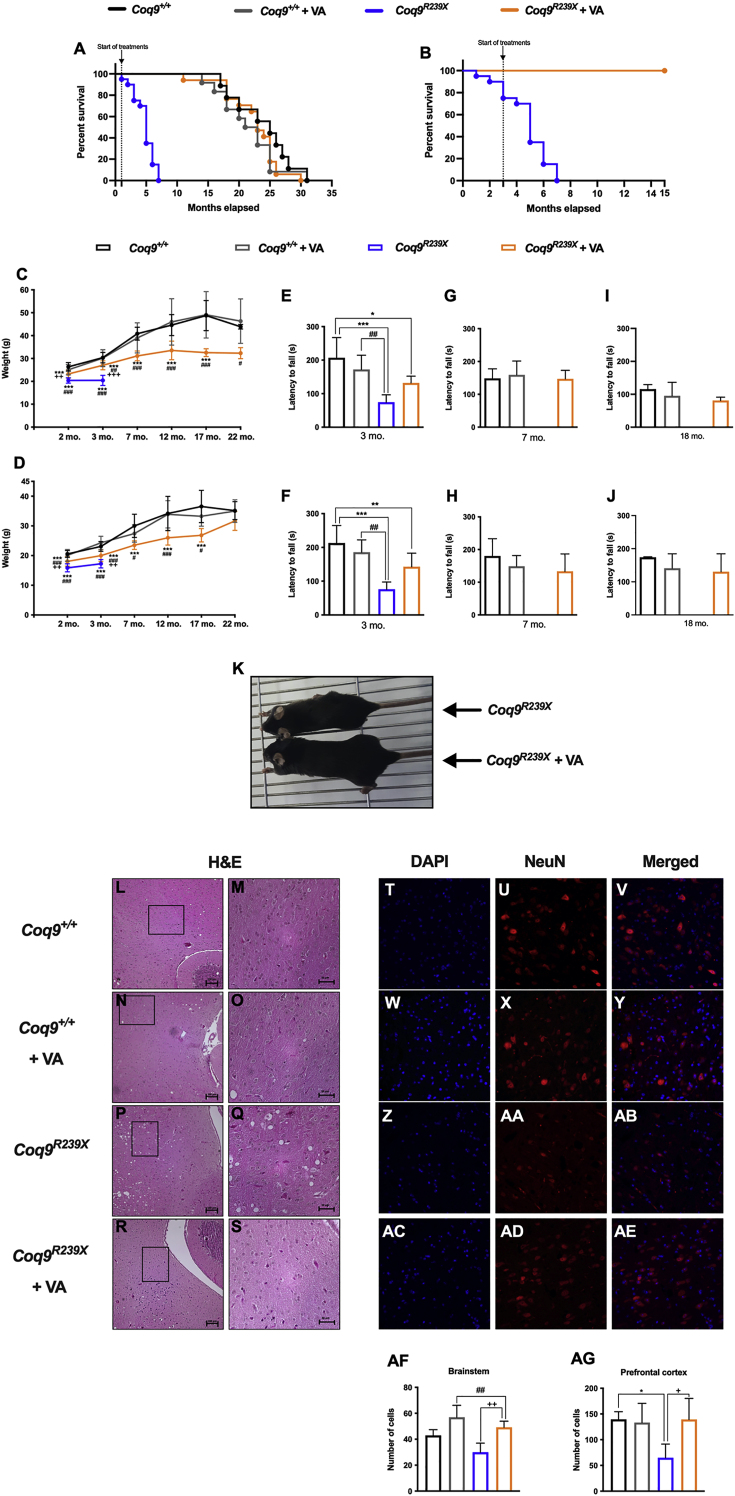
**Survival, phenotypic characterization and pathological features of the brain of *Coq9***^***R239X***^**mice after VA treatment.** (**A**) Survival curve of *Coq9*^*+/+*^ mice, *Coq9*^*+/+*^ mice under 1% VA supplementation, *Coq9*^*R239X*^ mice and *Coq9*^*R239X*^ mice under 1% VA supplementation. The treatments started at 1 month of age [Log-rank (Mantel-Cox) test or Gehan-Breslow-Wilcoxon test]. *Coq9*^*+/+*^, n = 10; *Coq9*^*+/+*^after VA treatment, n = 15; *Coq9*^*R239X*^, n = 20; *Coq9*^*R239X*^ after VA treatment, n = 17. (**B**) Survival curve of *Coq9*^*R239X*^ mice and *Coq9*^*R239X*^ mice after 1% VA treatment started at 3 months of age. *Coq9*^*R239X*^, n = 20; *Coq9*^*R239X*^ after VA treatment, n = 7. (**C**) Body weight of male *Coq9*^*+/+*^ mice, *Coq9*^*+/+*^ mice under 1% VA supplementation, *Coq9*^*R239X*^ mice and *Coq9*^*R239X*^ mice after 1% VA supplementation. *Coq9*^*+/+*^, n = 17; *Coq9*^*+/+*^after VA treatment, n = 19; *Coq9*^*R239X*^, n = 18; *Coq9*^*R239X*^ after VA treatment, n = 22. (**D**) Body weight of female *Coq9*^*+/+*^ mice, *Coq9*^*+/+*^ mice under 1% VA supplementation, *Coq9*^*R239X*^ mice and *Coq9*^*R239X*^ mice after 1% VA supplementation. *Coq9*^*+/+*^, n = 20; *Coq9*^*+/+*^after VA treatment, n = 15; *Coq9*^*R239X*^, n = 12; *Coq9*^*R239X*^ after VA treatment, n = 20. (**E**, **G** and **I**) Rotarod test of male *Coq9*^*+/+*^ mice, *Coq9*^*+/+*^ mice under 1% VA supplementation, *Coq9*^*R239X*^ mice and *Coq9*^*R239X*^ mice under 1% VA supplementation at 3 months of age (**E**), 7 months of age (**G**) and 18 months of age (**I**). n = 9–16 for each group. (**F**, **H** and **J**) Rotarod test of female *Coq9*^*+/+*^ mice, *Coq9*^*+/+*^ mice under 1% VA supplementation, *Coq9*^*R239X*^ mice and *Coq9*^*R239X*^ mice under 1% VA supplementation at 3 months of age (**H**), 7 months of age (**I**) and 18 months of age (**J**). n = 9–16 for each group. (**K**) Comparative image of a *Coq9*^*R239X*^ mouse and a *Coq9*^*R239X*^ mouse after 1% VA treatment at 3 months of age. (**L**–**S**) H&E stain in the brainstem of *Coq9*^*+/+*^ mice (**L** and **M**), *Coq9*^*+/+*^ mice under 1% VA supplementation (**N** and **O**), *Coq9*^*R239X*^ mice (**P** and **Q**) and *Coq9*^*R239X*^ mice under 1% VA supplementation (**R** and **S**) at 3 months of age. (**T**-**AE**) NeuN stain in the brainstem of *Coq9*^*+/+*^ mice (**T, U** and **V**), *Coq9*^*+/+*^ mice under 1% VA supplementation (**W, X** and **Y**), *Coq9*^*R239X*^ mice (**Z, AA** and **AB**) and *Coq9*^*R239X*^ mice under 1% VA supplementation (**AC, AD** and **AE**) at 3 months of age. (**AF** and **AG**) Number of neurons in brainstem (**AF**) and prefrontal cortex (**AG**) of *Coq9*^*+/+*^ mice, *Coq9*^*+/+*^ mice under 1% VA supplementation, *Coq9*^*R239X*^ mice and *Coq9*^*R239X*^ mice under 1% VA supplementation at 3 months of age. Data are expressed as mean ± SD. *P < 0.05, **P < 0.01, ***P < 0.001, differences *versus Coq9*^*+/+*^; ^#^P < 0.05, ^##^P < 0.01, ^###^P < 0.001, differences *versus Coq9*^*+/+*^ after VA treatment; +P < 0.05, ++P < 0.01, +++P < 0.001, *versus Coq9*^*R239X*^; (one-way ANOVA with a Tukey's *post hoc* test; n = 5–22 for each group).

We have previously characterized *Coq9*^*R239X*^ mice as a model of fatal mitochondrial encephalopathy with widespread CoQ deficiency and accumulation of DMQ. The deficit in CoQ induces a brain-specific impairment of mitochondrial bioenergetics performance which lead to neuronal death and demyelinization with severe vacuolization and astrogliosis in the brain of *Coq9*^*R239X*^ mice that consequently die between 3 and 7 months of age [13]. Oral supplementation with β-RA at a concentration of 1% or 0.33% (w/w) increased the survival of *Coq9*^*R239X*^ mice to a maximum lifespan of 25 or 34 months, respectively [15,22]. Similarly, here, we show that oral supplementation with VA, starting at 1 month of age, resulted in a remarkable increase in the survival of the mutant mice, with a maximum lifespan of 30 months and a median survival of 22 months, which was comparable to the survival of wild-type mice (Fig. 1A). Additionally, *Coq9*^*R239X*^ mice treated with VA starting at 3 months of age exceeded the lifespan of the mutant mice without treatment and remained alive until at least 15 months of age (Fig. 1B). Therefore, VA shows therapeutic benefits when the treatment starts at different stages of disease progression. The survival of the wild-type mice was not significantly modified by supplementation with VA (Fig. 1A).

The survival increase induced by VA in *Coq9*^*R239X*^ mice was accompanied by a phenotypic rescue. The body weight of *Coq9*^*R239X*^ mice treated with VA significantly increased compared with that of *Coq9*^*R239X*^ mice, in both males (Fig. 1C) and females (Fig. 1D). In contrast to β-RA [22], VA did not alter the body weight of wild-type mice (Fig. 1C and D). Treatment with VA also improved the motor coordination in *Coq9*^*R239X*^ mice. The latency to fall decreased in *Coq9*^*R239X*^ mice compared to wild-type mice at 3 months of age, and supplementation with VA produced a slight increase in this parameter in the mutant, while no differences were detectable in wild-type mice (Fig. 1E and F). Subsequent rotarod assays at 7 and 18 months of age showed that the improvement in the motor phenotype of the treated *Coq9*^*R239X*^ mice persisted over time (Fig. 1G–J). The phenotypic rescue was easily identifiable by an overall improvement in the health status of the treated *Coq9*^*R239X*^ mice (Fig. 1K and Movie S1)

Supplementary data related to this article can be found at https://doi.org/10.1016/j.redox.2022.102403.

The following is the supplementary data related to this article:

Multimedia component 1 Multimedia component 1

Spongiform degeneration is absent in *Coq9*^*+/+*^ mice before (Fig. 1L and M) and after VA supplementation (Fig. 1N and O) but is a histopathological feature of the encephalopathy in *Coq9*^*R239X*^ mice (Fig. 1P and Q) [13]. This spongiosis clearly diminished in the brainstem (Fig. 1R and S) and diencephalon (Fig S2 A-H) of the mutant mice treated with VA. Additionally, as well as no changes were detected in the *Coq9*^*+/+*^ with and without the treatment (Fig. 1T–Y and AF), a significant decrease in neurons, marked as NeuN-positive cells, was detected in the brainstem of *Coq9*^*R239X*^ mice (Fig. 1Z-AB and AF) compared to wild-type mice. VA supplementation normalized the presence of NeuN-positive cells in the affected areas (Fig 1AC-AF). Similar results were achieved for the number of NeuN-positive cells in the prefrontal cortex (Fig. 1AG; Fig S2 I-T). In contrast, the kidneys, liver and spleen of untreated and treated *Coq9*^*R239X*^ mice and wild-type mice showed similar structures, with no differences between the experimental groups (Fig. S2). In wild-type mice, treatment with VA did not induce morphological alterations in the kidneys, liver, or spleen (Fig. S2).

### 2.2 VA induces tissue-specific modulation of CoQ metabolism

**Fig. 2:**
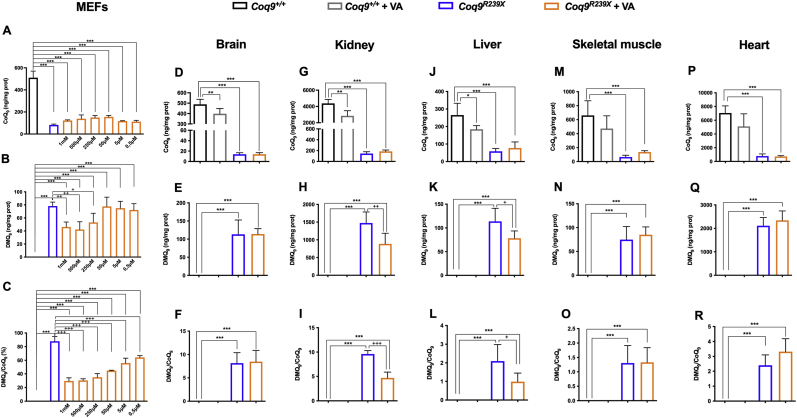
Levels of CoQ_9_, DMQ_9_ and DMQ_9_/CoQ_9_ ratio in *Coq9*^*R239X*^ mice MEFs and tissues after VA treatment. (**A**, **B** and **C**) Levels of CoQ_9_ (**A**), DMQ_9_ (**B**) and DMQ_9_/CoQ_9_ ratio (**C**) in MEFs from *Coq9*^*+/+*^ and *Coq9*^*R239X*^ mice after the supplementation with VA at 1 mM, 500 μM, 250 μM, 50 μM, 5 μM and 0,5 μM (**D-R**) Levels of CoQ_9_ (**D, G, J, M** and **P**), DMQ_9_ (**E, H, K, N** and **Q**) and DMQ_9_/CoQ_9_ ratio (**F, I, L, O** and **R**) in brain (**D, E** and **F**), kidney (**G, H** and **I**), liver (**J, K** and **L**), skeletal muscle (**M, N** and **O**) and heart (**P, Q** and **R**) of *Coq9*^*+/+*^ mice, *Coq9*^*+/+*^ mice after 1% VA treatment, *Coq9*^*R239X*^ mice and *Coq9*^*R239X*^ mice after 1% VA treatment. Data are expressed as mean ± SD. *P < 0.05, **P < 0.01, ***P < 0.001, differences *versus Coq9*^*+/+*^; +P < 0.05, ++P < 0.01, +++P < 0.001, *versus Coq9*^*R239X*^; (one-way ANOVA with a Tukey's *post hoc* test; n = 5 for each group).

Since VA is an analog of the natural precursor of the CoQ biosynthetic pathway, we investigated whether the rescue of the phenotype observed after VA treatment was due to the modulation of CoQ biosynthesis. To that end, we measured the levels of CoQ_9_, DMQ_9_ (major forms of CoQ and DMQ in rodents) and DMQ_9_/CoQ_9_ ratio, as molecular responsible of the progression of the clinical symptoms in some cases of CoQ deficiency [15,22], in mouse embryonic fibroblasts (MEFs) and in the most relevant tissues of *Coq9*^*R239X*^ mice. MEFs and tissues from *Coq9*^*R239X*^ mice show severe deficiency of CoQ_9_, accumulation of DMQ_9_ and an increased DMQ_9_/CoQ_9_ ratio [13,15] (Fig. 2). Treatment with VA increased levels of CoQ_9_, decreased levels of DMQ_9_ and decreased DMQ_9_/CoQ_9_ ratio in a dose-dependent manner (Fig. 2A, B and 2C). *In vivo*, supplementation with VA did not modify the CoQ_9_ levels (Fig. 2D), the DMQ_9_ levels (Fig. 2E) or the DMQ_9_/CoQ_9_ ratio (Fig. 2F) in the brain. However, VA treatment produced a slight increase in CoQ_9_ levels and significantly decreased the DMQ_9_ levels and the DMQ_9_/CoQ_9_ ratio in the kidneys (Fig. 2G–I) and the liver (Fig. 2J-L). A slight increase in CoQ_9_ levels was also detected in skeletal muscle (Fig. 2M) of the mutant mice after VA treatment but not in DMQ_9_ levels or the DMQ_9_/CoQ_9_ ratio (Fig. 2N and O). No changes were detected in the heart after the treatment (Fig. 2P–R) (Fig. S3). These results are similar to those previously obtained with β-RA, although they were less pronounced [15]. In wild-type animals, VA induced a slight decrease in CoQ_9_ levels (Fig. 2D, G, J, M and P).

To determine whether the tissue-specific differences in the response of the CoQ biosynthetic pathway to VA supplementation could be due to the bioavailability of this compound, we analyzed the levels of VA in the brain (Fig. S4A), kidneys (Fig. S4B) and liver (Fig. S4C) of *Coq9*^*R239X*^ mice after oral supplementation with VA. The brain showed the lowest values of VA, although those were in the same range than those in the liver. Thus, the bioavailability of VA is not the only reason for the tissue-specific differences in CoQ biosynthesis, although that requires further investigation.

**Fig. 3:**
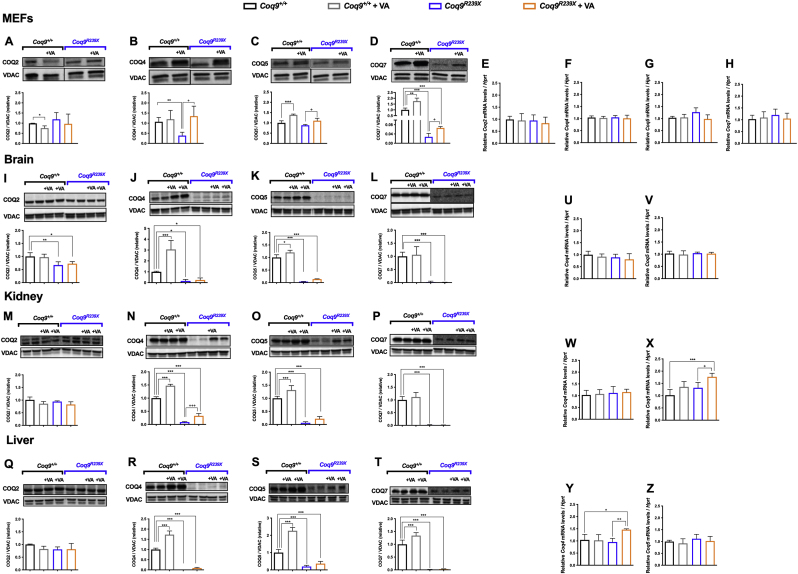
Effect of VA treatment on the levels of CoQ biosynthetic proteins and mRNA levels in the MEFs and tissues of *Coq9*^*R239X*^ mice. (**A-D**) Representative images of Western blots of the CoQ biosynthetic proteins COQ2 (**A**), COQ4 (**B**), COQ5 (**C**) and COQ7 (**D**), and the quantitation of the protein bands in MEFs from *Coq9*^*+/+*^ and *Coq9*^*R239X*^ mice after supplementation with VA at 1 mM (**E-H**) Relative mRNA levels for the genes *Coq2* (**E**), *Coq4* (**F**), *Coq5* (**G**) and *Coq7* (**H**) in MEFs from *Coq9*^*+/+*^ and *Coq9*^*R239X*^ mice after supplementation with VA at 1 mM (**I-L**) Representative images of Western blots of the CoQ biosynthetic proteins COQ2 (**I),** COQ4 (**J**), COQ5 (**K**) and COQ7 (**L**), and the quantitation of the protein bands in the brain of *Coq9*^*+/+*^ mice, *Coq9*^*+/+*^ mice after 1% VA treatment, *Coq9*^*R239X*^ mice and *Coq9*^*R239X*^ mice after 1% VA treatment. (**M-P**) Representative images of Western blots of the CoQ biosynthetic proteins COQ2 (**M),** COQ4 (**N**), COQ5 (**O**) and COQ7 (**P**), and the quantitation of the protein bands in the kidney of *Coq9*^*+/+*^ mice, *Coq9*^*+/+*^ mice after 1% VA treatment, *Coq9*^*R239X*^ mice and *Coq9*^*R239X*^ mice after 1% VA treatment. (**Q-T**) Representative images of Western blots of the CoQ biosynthetic proteins COQ2 (**Q),** COQ4 (**R**), COQ5 (**S**) and COQ7 (**T**), and the quantitation of the protein bands in the liver of *Coq9*^*+/+*^ mice, *Coq9*^*+/+*^ mice after 1% VA treatment, *Coq9*^*R239X*^ mice and *Coq9*^*R239X*^ mice after 1% VA treatment. **(U** and **V**) Relative mRNA levels of the genes *Coq4* (**U**) and *Coq5* (**V**) in brain from *Coq9*^*+/+*^ and *Coq9*^*R239X*^ mice after the supplementation with VA at 1 mM **(W** and **X**) Relative mRNA levels of the genes *Coq4* (**W**) and *Coq5* (**X**) in the kidneys from *Coq9*^*+/+*^ and *Coq9*^*R239X*^ mice after the supplementation with VA at 1 mM **(Y** and **Z**) Relative mRNA levels of the genes *Coq4* (**Y**) and *Coq5* (**Z**) in the liver from *Coq9*^*+/+*^ and *Coq9*^*R239X*^ mice after the supplementation with VA at 1 mM. Data are expressed as mean ± SD. *P < 0.05, **P < 0.01, ***P < 0.001, differences *versus Coq9*^*+/+*^; +P < 0.05, ++P < 0.01, +++P < 0.001, *versus Coq9*^*R239X*^; (one-way ANOVA with a Tukey's *post hoc* test; n = 3 for each group for cell samples, n = 5 for each group for tissue samples).

To determine whether VA interferes with the Complex Q, we analyzed the levels of the protein components of Complex Q. MEFs from *Coq9*^*R239X*^ mice showed normal levels of COQ2 and COQ5 but decreased levels of COQ4 and COQ7 (Fig. 3A–D) (Fig. S5). Supplementation with VA at a concentration of 1 mM increased the levels of COQ4 (Fig. 3B), COQ5 (Fig. 3C) and COQ7 (Fig. 3D) (Fig. S5). These increases in protein levels were not due to increases in the mRNA levels (Fig. 3E–H). *In vivo*, the levels of COQ2, COQ4, COQ5 and COQ7 were decreased in the brain (Fig. 3I and J, K and L) and, to a greater extent, except for COQ2, in the kidneys (Fig. 3M − P) and liver (Fig. 3Q–T) (Fig. S5) of *Coq9*^*R239X*^ mice compared to the same tissues in *Coq9*^*+/+*^ mice. Treatment with VA increased the levels of COQ4 and COQ5 in the kidneys (Fig. 3N and O) and to a smaller extent in the liver (Fig. 3R and S) (Fig. S5). However, the corresponding mRNA levels were not altered (Fig. 3U–Z), only increased for *Coq5* in the kidneys (Fig. 3X) and *Coq4* in the liver (Fig. 3Y) in the treated *Coq9*^*R239X*^ mice. In contrast, the levels of COQ2, COQ4, COQ5 and COQ7 in the brain were not altered after the treatment (Fig. 3I-L) (Fig. S5). This is consistent with the data previously reported for treatment with β-RA in the same animal model [15], although VA has a more intense effect on the upregulation of COQ4. In samples from *Coq9*^*+/+*^mice, supplementation with VA also produced changes in the levels of CoQ biosynthetic proteins. In *Coq9*^*+/+*^ MEFs, VA decreased the levels of COQ2 (Fig. 3A) but increased the levels of COQ5 (Fig. 3C) and COQ7 (Fig. 3D) (Fig. S5). *In vivo*, the levels of COQ4 and COQ5 were significantly increased in the brain (Fig. 3J and K), kidneys (Fig. 3N and O) and liver (Fig. 3R and S) (Fig. S5) in *Coq9*^*+/+*^ mice treated with VA. The levels of COQ7 were also increased in the liver in the treated *Coq9*^*+/+*^mice (Fig. 3T) (Fig. S5). Together, these results indicate that VA increases the levels of COQ4 and COQ5 in both *Coq9*^*+/+*^ and *Coq9*^*R239X*^ mice, probably via a compensatory mechanism caused by competition, with higher km, with 4-hydroxibezoic acid (4-HB), the natural substrate for COQ2 [23].

### 2.3 VA induces a DMQ/CoQ ratio-dependent increase in mitochondrial bioenergetics

**Fig. 4:**
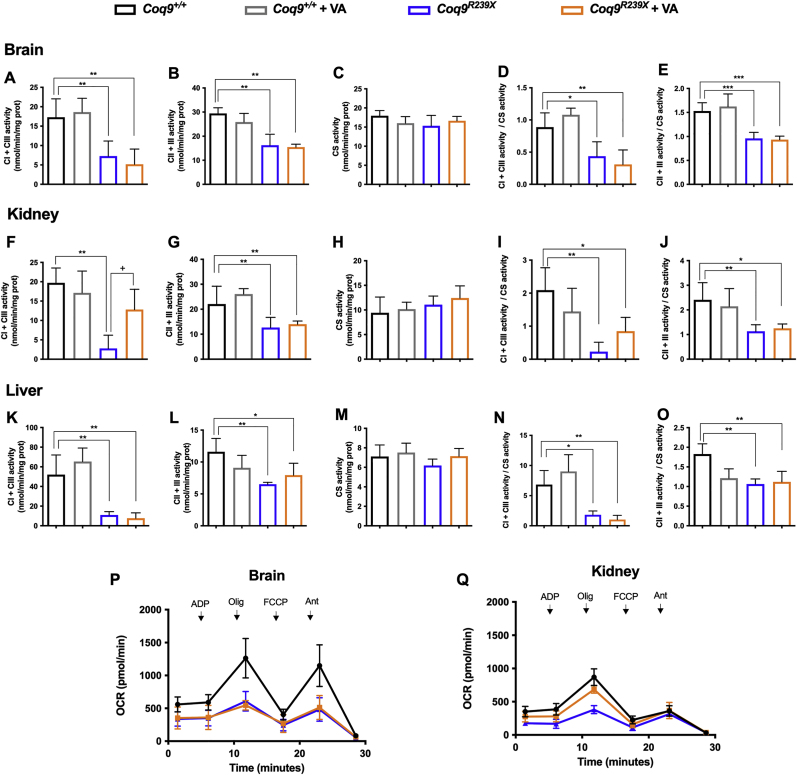
Tissue mitochondrial function after VA treatment in *Coq9*^*R239X*^ mice. (**A, F** and **K**) CoQ-dependent Complex I+III activities in the brain (**A**), kidneys (**F**) and liver (**K**) of *Coq9*^*+/+*^ mice, *Coq9*^*+/+*^ mice after 1% VA treatment, *Coq9*^*R239X*^ mice and *Coq9*^*R239X*^ mice after 1% VA treatment. (**B, G** and **L**) CoQ-dependent Complex II+III activities in the brain (**B**), kidneys (**G**) and liver (**L**) of *Coq9*^*+/+*^ mice, *Coq9*^*+/+*^ mice after 1% VA treatment, *Coq9*^*R239X*^ mice and *Coq9*^*R239X*^ mice after 1% VA treatment. (**C, H** and **M**) Citrate synthase activity of the brain (**C**), kidneys (**H**) and liver (**M**) in *Coq9*^*+/+*^ mice, *Coq9*^*+/+*^ mice after 1% VA treatment, *Coq9*^*R239X*^ mice and *Coq9*^*R239X*^ mice after 1% VA treatment. (**D, I** and **N**) CoQ-dependent Complex I+III activities normalized by citrate synthase activity in the brain (**D**), kidneys (**I**) and liver (**N**) of *Coq9*^*+/+*^ mice, *Coq9*^*+/+*^ mice after 1% VA treatment, *Coq9*^*R239X*^ mice and *Coq9*^*R239X*^ mice after 1% VA treatment. (**E, J** and **O**) CoQ-dependent Complex II+III activities normalized by citrate synthase activity in the brain (**E**), kidney (**J**) and liver (**O**) of *Coq9*^*+/+*^ mice, *Coq9*^*+/+*^ mice after 1% VA treatment, *Coq9*^*R239X*^ mice and *Coq9*^*R239X*^ mice after 1% VA treatment. (**P** and **Q**) Mitochondrial oxygen consumption rate (represented as State 3°, in the presence of ADP and substrates) in the brain (**P**) and kidneys (**Q**) of *Coq9*^*+/+*^ mice, *Coq9*^*R239X*^ mice and *Coq9*^*R239X*^ mice after 1% VA treatment. The data represent three technical replicates and the figures are representative of three biological replicates. *Coq9*^*+/+*^ mice, *Coq9*^*R239X*^ mice and *Coq9*^*R239X*^ mice after 1% VA treatment were included in all graphs. ADP = adenosine diphosphate; Olig = oligomycin; FCCP = carbonyl cyanide-p-trifluoromethoxyphenylhydrazone; Ant = antimycin A. Data are expressed as mean ± SD. *P < 0.05, **P < 0.01, ***P < 0.001, differences *versus Coq9*^*+/+*^; +P < 0.05, ++P < 0.01, +++P < 0.001, *versus Coq9*^*R239X*^; (one-way ANOVA with a Tukey's *post hoc* test; n = 5 for each group).

Due to the role of CoQ in mitochondrial function and due to the fact that VA partially modulates CoQ biosynthesis, we evaluated the effect of the treatment on mitochondrial bioenergetics. In the brain, kidneys and liver of *Coq9*^*R239X*^ mice, the activities of CoQ-dependent mitochondrial complexes I+III (CI+III) and CII+III (Fig. 4A-O) and the mitochondrial oxygen consumption rate (OCR) (Fig. 4P–Q) were significantly decreased compared to the values in wild-type animals. Treatment with VA did not improve any of these parameters in the brain of the treated mutant mice (Fig. 4A–E and P). In the kidneys, however, VA treatment normalized the CI+III activity (Fig. 4F and I) and induced a significant increase in OCR (Fig. 4Q). Furthermore, VA treatment partially normalized CII+III activity (Fig. 4L) in the liver. Overall, these results demonstrate that the tissue-specific responses of CoQ metabolism to VA determine the extent of improvement in mitochondrial bioenergetics, a fact that was also previously observed with β-RA therapy [15].

### 2.4 4-HB analogs induce anti-neuroinflammatory effects

**Fig. 5:**
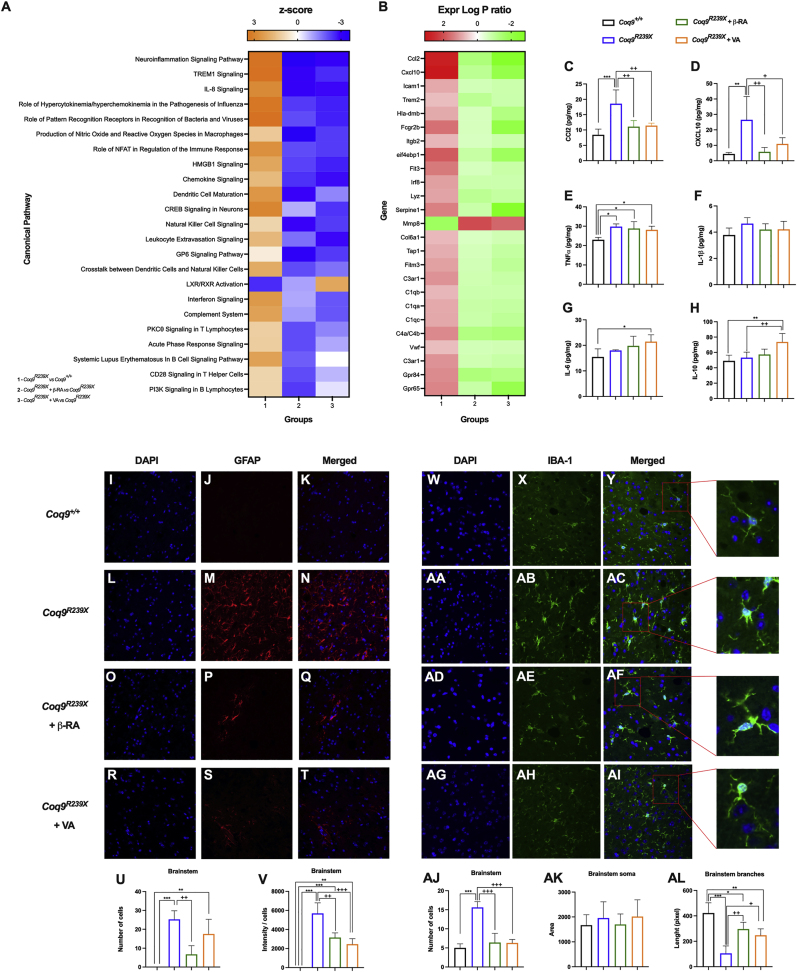
Gene expression profile and characterization of the neuroinflammatory status after treatment of *Coq9*^*R239X*^ mice with 4-HB analogs. (**A**) Representative heatmap of the canonical pathways altered by the mutation and normalized by either β-RA or VA treatments in the brainstem of *Coq9*^*+/+*^ mice, *Coq9*^*R239X*^ mice, *Coq9*^*R239X*^ mice after 1% β-RA treatment and *Coq9*^*R239X*^ mice after 1% VA treatment at 3 months of age. (**B**) Representative heatmap of the expression level of the genes altered by the mutation and normalized by either β-RA or VA treatments in the brainstem of *Coq9*^*+/+*^ mice, *Coq9*^*R239X*^ mice, *Coq9*^*R239X*^ mice after 1% β-RA treatment and *Coq9*^*R239X*^ mice after 1% VA treatment at 3 months of age. (**C–H**) Levels of the cytokines CCl2 (**C**), CXCL10 (**D**), TNFα (**E**), IL-1β (**F**), IL-6 (**G**) and IL-10 (**H**) in the brainstem of *Coq9*^*+/+*^ mice, *Coq9*^*R239X*^ mice, *Coq9*^*R239X*^ mice after 1% β-RA treatment and *Coq9*^*R239X*^ mice after 1% VA treatment at 3 months of age. (**I**–**T**) GFAP stain in the brainstem of *Coq9*^*+/+*^ mice (**I, J** and **K**), *Coq9*^*R239X*^ mice (**L, M** and **N**), *Coq9*^*R239X*^ mice under 1% β-RA supplementation (**O, P** and **Q**) and *Coq9*^*R239X*^ mice under 1% VA supplementation (**R, S** and **T**) at 3 months of age. (**U** and **V**) Quantification of GFAP expression by number of cells (**U**) and intensity/cells (**V**) in the brainstem of *Coq9*^*+/+*^ mice, *Coq9*^*R239X*^ mice, *Coq9*^*R239X*^ mice under 1% β-RA supplementation and *Coq9*^*R239X*^ mice under 1% VA supplementation at 3 months of age. (**W**-**AI**) IBA-1 stain in the brainstem of *Coq9*^*+/+*^ mice (**W, X** and **Y**), *Coq9*^*R239X*^ mice (**AA, AB** and **AC**), *Coq9*^*R239X*^ mice under 1% β-RA supplementation (**AD, AE** and **AF**) and *Coq9*^*R239X*^ mice under 1% VA supplementation (**AG, AH** and **AI**) at 3 months of age. (**AJ, AK** and **AL**) Quantification of IBA-1 expression by number of cells (**AJ**) and phenotype analysis of soma area (**AK**) and branches length (**AL**) in the brainstem of *Coq9*^*+/+*^ mice, *Coq9*^*R239X*^ mice, *Coq9*^*R239X*^ mice under 1% β-RA supplementation and *Coq9*^*R239X*^ mice under 1% VA supplementation at 3 months of age. Data are expressed as mean ± SD. *P < 0.05, **P < 0.01, ***P < 0.001, differences *versus Coq9*^*+/+*^; +P < 0.05, ++P < 0.01, +++P < 0.001, *versus Coq9*^*R239X*^; (one-way ANOVA with a Tukey's *post hoc* test; n = 5 for each group).

Keeping in mind the previously described results, as well as the similarity between them and the results previously reported in *Coq9*^*R239X*^ mice treated with β-RA [15,22], we decided to analyze the transcriptomic profiles of *Coq9*^*R239X*^ mice treated with either VA or β-RA to find potential common therapeutic mechanisms. We thus carried out an RNA-Seq experiment on the mouse brainstem, since we did not find cerebral changes neither in CoQ metabolism or mitochondrial bioenergetics, even though the encephalopathic phenotype was rescued with both treatments. For this analysis, we divided the mice into four experimental groups: *Coq9*^*+/+*^, *Coq9*^*R239X*^, *Coq9*^*R239X*^ treated with 1% β-RA and *Coq9*^*R239X*^ treated with 1% VA. The analysis revealed upregulation of canonical pathways related to the inflammatory signaling pathway in *Coq9*^*R239X*^ mice compared to *Coq9*^*+/+*^ mice (Fig. 5A). In particular, the genes *Ccl2* and *Cxcl10*, which encode the proinflammatory cytokines CXCL10 and CCL2, respectively, were highly expressed in the mutant mice compared to the *Coq9*^*+/+*^ mice (Fig. 5B). This was confirmed by quantifying the gene expression of *Ccl2* and *Cxcl1*0 by quantitative real-time PCR (Figs. S6A–B) and determining CCL2 and CXC10 levels with a ProcartaPlex Inmunoassay (Fig. 5C and D). Remarkably, treatment with either VA or β-RA normalized the levels of the altered genes (Fig. 5A and B; Figs. S6A–B), as well as the levels of the cytokines (Fig. 5C and D). Other neuroinflammatory genes were also upregulated in the brainstem of *Coq9*^*R239X*^ mice (Fig. 5A and B) and repressed under both treatments (Fig. 5A and B). Moreover, the proinflammatory cytokine IL-1β showed a trend toward decreased levels (Fig. 5E and F), while the anti-inflammatory cytokines IL-6 and IL-10 showed a trend toward increased levels (Fig. 5G and H) under both treatments.

Neuroinflammation in neurodegenerative diseases is typically mediated by gliosis. Accordingly, compared to *Coq9*^*+/+*^ mice (Fig. 5I, J and K; Fig S6C, D and E), the brainstem (Fig. 5L, M, and N) and cerebellum (Fig S6F, G and H) of *Coq9*^*R239X*^ mice showed features of reactive astrogliosis [13]. Both the β-RA and VA treatments significantly diminished the number and intensity of reactive astrocytes, marked as GFAP-positive cells, in the brainstem (Fig. 5O–V) and cerebellum (Fig. S6I-P). Additionally, proliferation of microglia, marked as IBA-1-positive cells, compared to the wild-type mice (Fig. 5W, X and Y), was apparent in the brainstem of the mutant mice (Fig. 5AA, AB, AC and AJ). Moreover, the microglia in the brainstem of *Coq9*^*R239X*^ mice showed a reactive morphology, with hypertrophy, thickened soma, and short branches, which are the typical features of a proinflammatory state (Fig. 5 AA, AB, AC, AK and AL). Supplementation with either β-RA or VA reduced the number and reactivity of microglia (Fig. 5AD-AL), thus promoting an anti-inflammatory phenotype (Fig. 5AK and AL). The individual effects of β-RA and VA on microglia distribution and reactivity were also observed in the prefrontal cortex and cerebellum (Fig. S7).

Together, these results reveal the antineuroinflammatory effects of both 4-HB analogs as a key therapeutic approach for rescuing the encephalopathic phenotype associated with CoQ deficiency.

### 2.5 4-HB analogs modify the levels of secreted proteins in the plasma

Since the observed antineuroinflammatory effects could be mediated by an indirect mechanism through potential endocrine factors, we analyzed the plasma proteome in the same groups. The analyses identified proteins whose levels were significantly altered in *Coq9*^*R239X*^ mice compared to wild-type mice, and normalized by both treatments. Specifically, 5 proteins (Fig. S8A) met that criterion, i.e.: SERPINA3, pregnancy zone protein (PZP), mannose-binding lectin-associated serine protease-1 (MASP1), collagen α-1 chain (COL1A1) and anaphylatoxin-like domain-containing protein (AI182371). Specifically, SERPINA3, MASP1, COL1A1 and AI182371 were downregulated in the mutant mice, and PZP was upregulated. Treatment with either β-RA or VA upregulated SERPINA3, MASP1, COL1A1 and AI182371 and downregulated PZP (Fig. S8B). Interestingly, some of these proteins have been related to immune functions, e.g., SERPINA 3 [24], MASP1 [25] and AI182371 (GO annotation), or to pathophysiological conditions of the CNS, e.g., SERPINA 3 [26] and PZP [27,28]. The levels of the other 11 proteins were also modified after β-RA and VA treatment compared to vehicle treatment, although they were not altered in *Coq9*^*R239X*^ mice compared to wild-type mice (Fig. S8A).

### 2.6 4-HB analogs normalize the mitochondrial proteome in the context of CoQ deficiency

Tissues from *Coq9*^*R239X*^ mice show an altered mitochondrial proteome [29]. Thus, to further evaluate the common therapeutic mechanism of 4-HB analogs, we performed quantitative proteomics analysis of mitochondrial fractions from the brain and kidneys of mice in the same experimental groups and with the previously described criteria. In the brain, proline dehydrogenase (PRODH) and Cx_9_C motif-containing protein 4 (CMC4) levels were significantly altered in *Coq9*^*R239X*^ mice compared to wild-type mice, and these changes were normalized by both treatments. PRODH, a CoQ-dependent enzyme involved in proline metabolism and the urea cycle, was upregulated in *Coq9*^*R239X*^ mice compared to wild-type mice, while CMC4, a protein with nonessential role in cytochrome *c* oxidase subassembly [30], was downregulated in the mutant mice. Treatment with either β-RA or VA reversed this pattern, as it induced a downregulation of PRODH and an upregulation of CMC4 (Figs. S8C and D; Fig. S9A). Moreover, nine additional proteins were also modified after β-RA and VA treatments compared to vehicle treatment, although they were not altered in *Coq9*^*R239X*^ mice compared to wild-type mice (Fig. S8C).

**Fig. 6:**
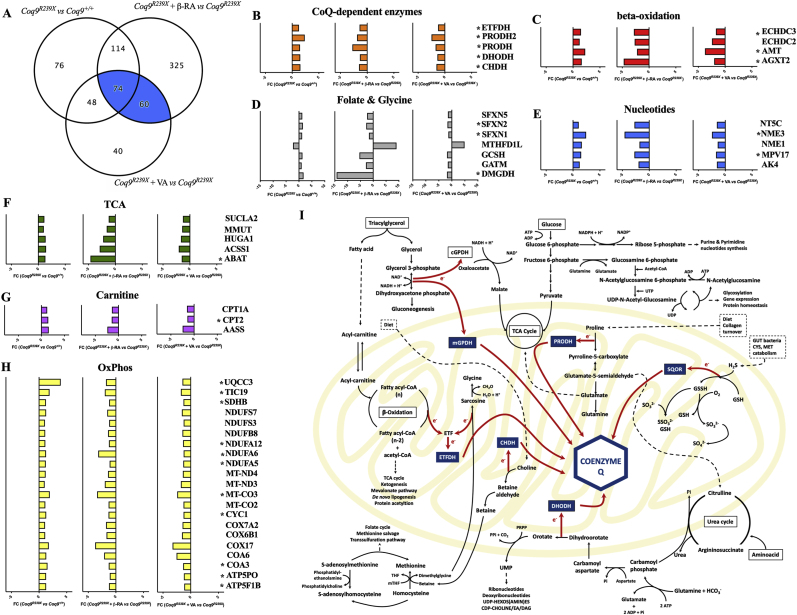
Mitochondrial proteome after 4-HB analogs treatment in the kidneys of *Coq9*^*R239X*^ mice. (**A**) Global differences in protein levels between experimental groups. The blue segment represents the 74 proteins altered by the mutation and by β-RA and VA treatments, and the 60 proteins modified by β-RA and VA treatments. (**B–H**) Fold change of the level of the proteins modified by the mutation and normalized by either β-RA or VA treatments in the renal mitochondrial proteome in the kidney of *Coq9*^*+/+*^ mice, *Coq9*^*R239X*^ mice, *Coq9*^*R239X*^ mice after 1% β-RA treatment and *Coq9*^*R239X*^ mice after 1% VA treatment at 3 months of age. Proteins are classified according to their functions in CoQ-dependent enzyme (**B**), beta-oxidation (**C**), folate and glycine (**D**), nucleotides (**E**), citric acid cycle (TCA) (**F**), carnitine (**G**) and OxPhos system (**H**). *p < 0.05. Mitochondrial proteomics was performed in isolated mitochondria. *Coq9*^*+/+*^, n = 5; *Coq9*^*R239X*^, n = 5; *Coq9*^*R239X*^ after β-RA treatment, n = 6; *Coq9*^*R239X*^ after VA treatment, n = 5. (**I**) Schematic representation of the Q-junction and its connection with the cell metabolism. . (For interpretation of the references to colour in this figure legend, the reader is referred to the Web version of this article.)

In the kidneys, 74 mitochondrial proteins met the previously described criteria, and 60 additional proteins were also modified with either β-RA or VA treatments compared to vehicle treatment, although they were not altered in *Coq9*^*R239X*^ mice compared to wild-type (Fig. 6A). All of these proteins were grouped in the MitoPathways [31] (Fig. 6B–H). Interestingly, the CoQ-dependent enzymes electron transfer flavoprotein dehydrogenase (ETFDH), proline dehydrogenase 2 (PRODH2), PRODH, dihydroorotate dehydrogenase (DHODH) and choline dehydrogenase (CHDH) were upregulated in *Coq9*^*R239X*^ mice compared to *Coq9*^*+/+*^ mice (Fig. 6B and I), probably as a response to low levels of CoQ. These proteins are part of the Q-junction (Fig. 6I) and they are directly or indirectly involved in fatty acid oxidation, branched-chain amino acid metabolism, lysine and glycine metabolism, choline and betaine metabolism, proline metabolism, glyoxylate and pyruvate metabolism, nucleotide metabolism, folate and 1-C metabolism, the urea and tricarboxylic acid (TCA) cycles, as well as the transsulfuration pathway and the methionine cycle (Fig. 6I) [3,4,31]. Most likely due to this alteration in the Q-junction and its linked pathways, upregulation of proteins involved in beta-oxidation (Fig. 6C), folate and glycine metabolism (Fig. 6D), nucleotide metabolism (Fig. 6E), the TCA cycle (Fig. 6F), the carnitine pathway (Fig. 6G) and the OxPhos system (Fig. 6H) was also detected in the mutant mice. Importantly, treatment with either β-RA or VA induced the downregulation of these proteins (Fig. 6B–H). The results for PRODH, DMGDH, OPA1 and CRYAB were validated by western blotting of tissue homogenates (Figs. S9B–E). Similar changes were observed for proteins related to protein synthesis, mitochondrial protein import, carriers, mitochondrial fusion, chaperones, and others (Fig. S10). Therefore, treatment with β-RA or VA partially normalizes the mitochondrial proteome disruption caused by dysfunctional Q-junction.

### 2.7 β-RA and VA restore the CoQ-dependent metabolism in the context of CoQ deficiency

**Fig. 7:**
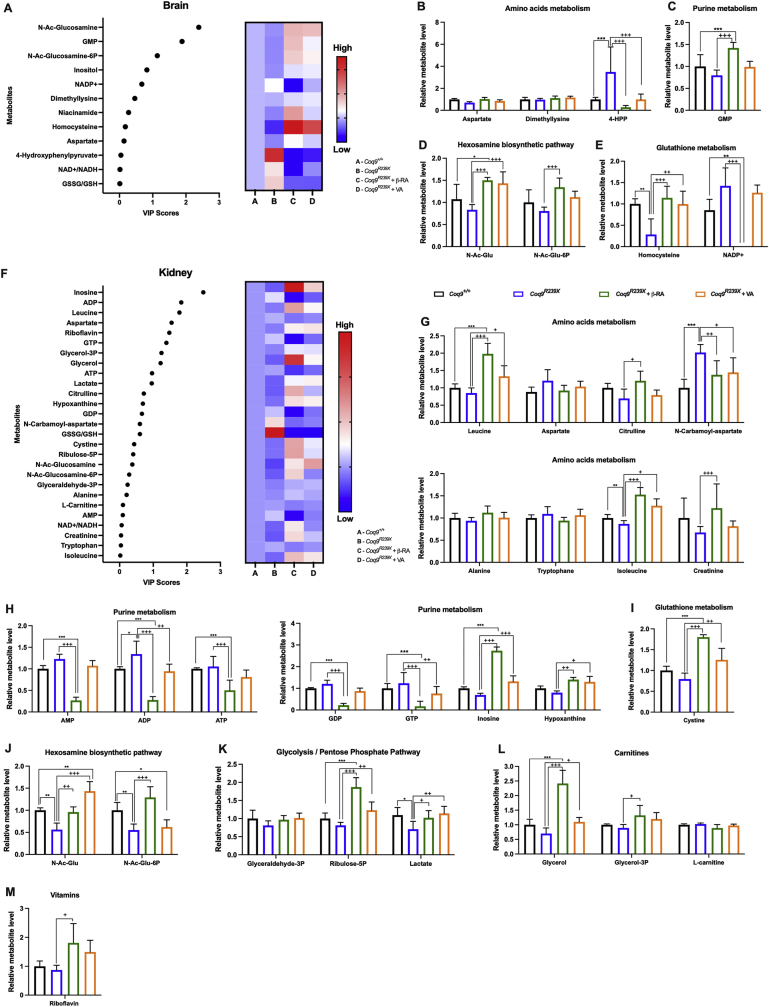
Effect of the treatment with 4-HB analogs in the cerebral and renal metabolism of *Coq9*^*R239X*^ mice. (**A**) Variable importance in projection (VIP) scores plots for the metabolites modified by the mutation and normalized by β-RA and VA treatments in the brain. The right heatmap indicates the metabolite levels for each experimental group. (**B-E**) Key metabolites related to amino acid metabolism (**B**), purine metabolism (**C**), hexosamines biosynthetic pathway (**D**) and glutathione metabolism (**E**) detected in the brain of *Coq9*^*+/+*^ mice, *Coq9*^*R239X*^ mice, *Coq9*^*R239X*^ mice after 1% β-RA treatment and *Coq9*^*R239X*^ mice after 1% VA treatment at 3 months of age. (**F**) Variable importance in projection (VIP) scores plots for the metabolites modified by the mutation and normalized by β-RA and VA treatments in the kidneys. The right heatmap indicates the metabolite levels for each experimental group. (**G-M**) Key metabolites related to amino acids metabolism (G), purine metabolism (H), glutathione metabolism (I), hexosamines biosynthetic pathway (J), glycolysis/pentose phosphate pathway (K), carnitines (L) and vitamins (M) detected in the kidneys of *Coq9*^*+/+*^ mice, *Coq9*^*R239X*^ mice, *Coq9*^*R239X*^ mice after 1% β-RA treatment and *Coq9*^*R239X*^ mice after 1% VA treatment at 3 months of age. Data are expressed as average peak area ±SD compared to wild-type. *P < 0.05, **P < 0.01, ***P < 0.001, differences *versus Coq9*^*+/+*^; +P < 0.05, ++P < 0.01, +++P < 0.001, *versus Coq9*^*R239X*^; (one-way ANOVA with a Tukey's *post hoc* test; n = 5 for each group).

**Fig. 8:**
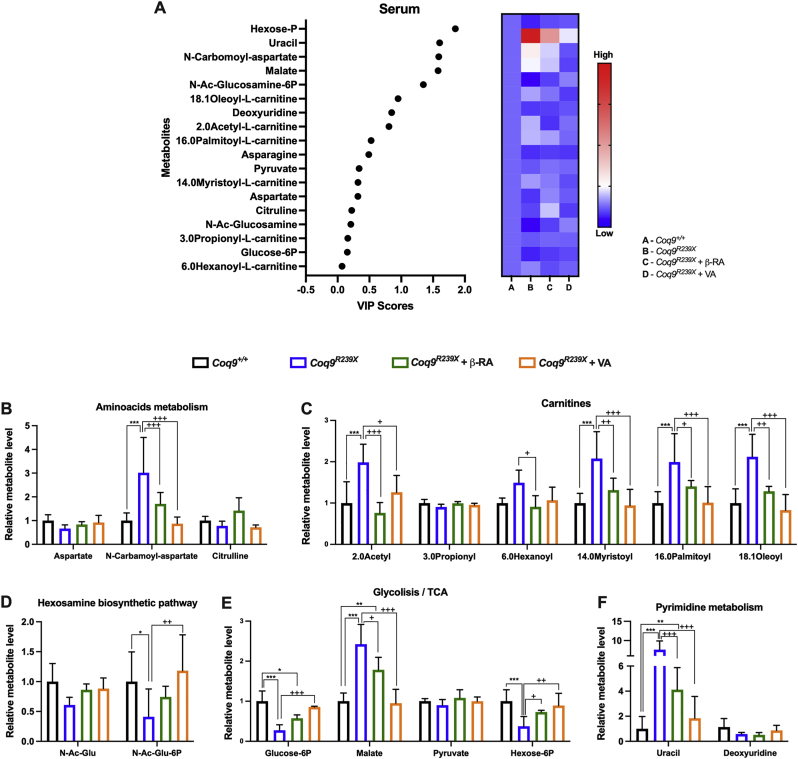
Effect of the treatment with 4-HB analogs on serum metabolites in *Coq9*^*R239X*^ mice. (**A**) Variable importance in projection (VIP) scores plots for the metabolites modified by the mutation and normalized by β-RA and VA treatments in the brain. The right heatmap indicates the metabolite levels for each experimental group. (**B–F**) Key metabolites related to amino acid metabolism (**B**), carnitines (**C**), hexosamines biosynthetic pathway (**D**), glycolysis/TCA (**E**) and pyrimidine metabolism (**F**) detected in the serum of *Coq9*^*+/+*^ mice, *Coq9*^*R239X*^ mice, *Coq9*^*R239X*^ mice after 1% β-RA treatment and *Coq9*^*R239X*^ mice after 1% VA treatment at 3 months of age. Data are expressed as average peak area ±SD compared to wild-type. *P < 0.05, **P < 0.01, ***P < 0.001, differences *versus Coq9*^*+/+*^; +P < 0.05, ++P < 0.01, +++P < 0.001, *versus Coq9*^*R239X*^; (one-way ANOVA with a Tukey's *post hoc* test; n = 5 for each group).

The changes in the mitochondrial proteome must lead to adaptations in mitochondrial metabolism. Thus, we explored the metabolic changes induced by β-RA and VA in the same experimental groups previously subjected to transcriptomics and proteomics analyses, following the same analytical criteria (Fig. 7A-M and Fig. 8A–F). Metabolites were also sorted based on their VIP score values (Fig. 7, Fig. 8A) and grouped in metabolic pathways based on an enrichment analysis (Fig. S11). In the brain (Fig. 7D), kidneys (Fig. 7J) and plasma (Fig. 8D), decreased levels of both N-Ac-glucosamine and N-Ac-glucosamine-6P levels were detected in the *Coq9*^*R239X*^ mice, compared to those in the wild-type mice. Both metabolites are involved in the hexosamine biosynthetic pathway, a branch of glycolysis that can be adapted to changes in the TCA cycle [32] (Fig. 6I). This pathway is involved in O-linked-N-acetylglucosaminylation (O-GlcNAcylation) (Fig. 6I), which is crucial for neuronal survival and is affected by aging and in neurodegenerative diseases [[33], [34], [35], [36], [37], [38], [39], [40], [41], [42], [43]]. Either β-RA or VA supplementation increased the levels of both metabolites in the mutant mice (Fig. 7, Fig. 8D), most likely due to overcompensation to normalize the TCA cycle.

It is also interesting that 4-hydroxyphenylpyruvate (4-HPP) accumulated in the brains of the mutant mice (Fig. 7B). This metabolite is an intermediate in the synthesis of the CoQ benzoquinone ring (Fig. S1), and therefore, a feedback mechanism induced by the defect in CoQ biosynthesis in *Coq9*^*R239X*^ mice could be the origin of this abnormality. Consistent with that possibility, either β-RA or VA restored 4-HPP levels (Fig. 7D), indicating that both compounds are used in the CoQ biosynthetic pathway.

In the kidneys, inosine and hypoxanthine levels were moderately decreased in the mutant mice compared to the wild-type mice, and the treatments with 4-HB analogs significantly increased the levels of both metabolites in *Coq9*^*R239X*^ mice (Fig. 7H). Additionally, some of the identified metabolites are related to the metabolic pathways linked to the enzymes of the Q-junction. For instance, 1) the catabolic pathway for the essential amino acids leucine and isoleucine is linked to ETFDH activity. Both leucine and isoleucine showed a trend toward decreased levels in *Coq9*^*R239X*^ mice compared to *Coq9*^*+/+*^ mice and a significant increase after either β-RA or VA supplementation (Fig. 7G). 2) Aspartate degradation is related to the urea cycle and to the activity of the CoQ-dependent enzymes PRODH and DHODH, while N-carbamoyl-aspartate is mainly connected to DHODH activity (Fig. 6I). The levels of aspartate and N-carbamoyl-aspartate increased in the mutant mice and to decrease after the treatments (Fig. 7G). 3) Glycerol-3P and glycerol are metabolites of the glycerol phosphate shuttle linked to GPDH activity (Fig. 6I). Both metabolites showed a trend toward decreased levels in the mutant mice, and both treatments led to an increase in their levels (Fig. 7L).

In the serum, five carnitines were highly increased in the serum of *Coq9*^*R239X*^ mice, i.e., 2.0 acetyl-l-carnitine, 6.0 hexanoyl-l-carnitine, 14.0 myristoyl-l-carnitine, 16.0 palmitoyl-l-carnitine and the 18.1 oleoyl-l-carnitine (Fig. 8C). Treatment with the 4-HB analogs significantly decreased the levels of these carnitine metabolites. However, the levels of 3.0 propionyl-l-carnitine were similar in the four experimental groups (Fig. 8C). These changes may be related to the levels and activity of the CoQ-dependent enzyme ETFDH, which participates in β-oxidation (Fig. 6I). In fact, an accumulation of carnitines in plasma has also been described in other models of CoQ deficiency [44,45]. The levels of N-carbamoyl-aspartate significantly increased in *Coq9*^*R239X*^ mice compared to wild-type mice, and supplementation with either β-RA or VA normalized these levels (Fig. 8B), similar to the results in the kidneys. The N-carbamoyl-aspartate is related to the CoQ-dependent enzyme DHODH and to the ribonucleotide metabolism (Fig. 6I). As a possible consequence, the uracil levels were significantly increased, while the deoxyuridine levels were decreased in the mutant mice (Fig. 8F). Supplementation with 4-HB analogs induced normalization of the levels of these metabolites. Since the metabolism of glucosamines is linked to the pentose phosphate pathway (PPP), changes in the glucosamine pathway could explain the modifications observed in the levels of hexose-P and glucose-6P (Fig. 8E). Hexose-P and glucose-6P levels significantly decreased in *Coq9*^*R239X*^ mice compared to the wild-type mice, and the β-RA and VA treatments normalized these levels. Together, the metabolic profiles reveal a metabolic adaptation in response to the disruption in the Q-junction and a powerful metabolic correction effect of the 4-HB analogs, even though the levels of CoQ and DMQ were not completely normalized.

## 3 Discussion

The metabolic consequences of low CoQ levels have not been clearly elucidated in the context of the multiple metabolic pathways linked to the Q-junction. Nevertheless, 4-HB analogs induce powerful therapeutic effects in different animal models of CoQ deficiency [[15], [16], [17], [18],^22^], although the precise mechanisms are not completely understood. Here, we answer these central scientific questions by demonstrating that low levels of CoQ induce an adaptation of the mitochondrial proteome, most likely due to the disruption of the Q-junction as a primary event. Additionally, CoQ deficiency, together with the accumulation of DMQ, induces reactive astrogliosis and microgliosis, promoting neuroinflammation and spongiform degeneration. Importantly, oral treatment with 4-HB analogs induces partial normalization of the DMQ/CoQ ratio, particularly in peripheral tissues, leading to profound normalization of the mitochondrial proteome and metabolism. Furthermore, 4-HB analogs reduce gliosis and neuroinflammation, with subsequent rescue of the encephalopathic phenotype in *Coq9*^*R239X*^ mice.

Different pathophysiological mechanisms have been previously identified as explanations for the clinical heterogeneity of CoQ deficiency. Mainly, a decline in ATP synthesis [13,[46], [47], [48]], increased oxidative stress [46,47,49,50], the disruption of the sulfide metabolism [51,52], and a defect in the *de novo* biosynthesis of pyrimidines [48] were previously described as consequences of CoQ deficiency. Using a more integrative approach, our study reveals that the disruption of the Q-junction is the origin of a metabolic disarrangement caused by CoQ deficiency. Interestingly, proteins of the Q-junction respond differently to CoQ deficiency and to the treatments with 4-HB analogs, e.g., 1) the levels of SQOR are decreased under CoQ deficiency and 4-HB analogs do not significantly reverse that change; 2) the levels of GPDH remain stable both under CoQ deficiency and after treatment with 4-HB analogs; and 3) the levels of PRODH, ETFDH, DHODH and CHDH are increased in the context of CoQ deficiency and normalized by treatment with 4-HB analogs. A similar specific response to CoQ deficiency has been recently shown in the *mon* zebrafish model, which exhibits CoQ deficiency and a preferential use of the CoQ pool by the DHODH during erythropoiesis [53]. Our study also shows tissue-specific differences in the Q-junction, which may contribute to the phenotype development and therapeutic success. Another remarkable finding of this work is the identification of neuroinflammation as a key feature and therapeutic target of the encephalopathic phenotype associated with CoQ deficiency. Neuroinflammation has been identified as a key pathologic mechanism in other mouse models of mitochondrial disease and neurodegeneration [54,55]. Neuroinflammatory mediators can be released by both reactive astrocytes and microglia [56], particularly in the brainstem, diencephalon, and cerebellum, resembling the pathological features of Leigh syndrome [57,58].

Therapeutically, previous studies have shown that β-RA rescues the phenotype of different models of CoQ deficiency [[15], [16], [17], [18],^22^]. In this study, we show that another 4-HB analog, VA, is also able to rescue the encephalopathic phenotype of *Coq9*^*R239X*^ mice. Importantly, we reveal common therapeutic mechanisms of both phenolic acids, mainly related to their specific effects on CoQ metabolism and the functioning of the Q-junction and the ability to reduce reactive gliosis, neuroinflammation and spongiosis. The effects on the Q-junction and related pathways are more intense in the kidneys than in the brain, suggesting that this particular effect could be mainly linked to the reduction in the DMQ/CoQ ratio. Thus, the accumulation of DMQ and the decrease in CoQ levels alter not only the function of CI+III [59] but also the activities of other dehydrogenases of the Q-junction. Accordingly, in the opposite context of supraphysiological levels of CoQ_10_, SQOR is upregulated, leading to a decrease in the levels of enzymes of the transsulfuration pathway and a subsequent adaptation of the serine biosynthesis and folate cycle [60]. The second therapeutic mechanism described here, the anti-inflammatory effects, could be directly or indirectly mediated by phenolic compounds and remains to be elucidated. Thus, some complementary hypotheses could explain the therapeutic effects of 4-HB analogs in the brain of *Coq9*^*R239X*^ mice. First, 4-HB analogs may have CoQ-independent functions with therapeutic potential for mitochondrial encephalopathies; second, 4-HB analogs may have effects in some specific cell types of the brain, a fact that may be masked in the analyses in the whole brain; and third, since 4-HB analogs may have some limitations to cross the blood brain barrier due to the binding to serum albumin [61,62], the observed effects may be due to tissue–brain crosstalk after the reduction of DMQ_9_/CoQ_9_ ratio in peripheral tissues and the subsequent improvement of the mitochondrial bioenergetics, thus leading to the reduction of gliosis, spongiosis, and neuroinflammation. In this context, a few plasma proteins with potential involvement in neuroinflammation have been identified. Additionally, these anti-inflammatory effects could explain the therapeutic benefits of β-RA in the *Coq6*^*podKO*^ and *Adck4*^*ΔPodocyte*^ mouse models, since an immune response has been described as part of the nephrotic phenotype associated with CoQ deficiency [63,64].

In addition to the common response to both β-RA and VA, the omics and molecular analyses also revealed some differences between the two treatments. For example, VA strongly upregulates COQ4 [12] and COQ5, while β-RA minimally affects the levels of these proteins [15]. Thus, the different effects of the two compounds on the DMQ/CoQ ratio could be explained by different mechanisms of action in the Complex Q, although this has not been yet investigated. Moreover, we generally observed more intense effects of β-RA in the DMQ/CoQ ratio and in the omics data than those of VA. Such differences might be important for potential translation into the clinic and for dose adjustment. Additionally, it is remarkable that only β-RA is able to decrease the white adipose tissue content in wild-type mice [22].

From the translational perspective, the omics data from the serum are also important, since they could potentially be useful biomarkers to follow the progression of the disease and the response to treatment. In particular, we found different types of potential biomarkers. 1) An increase in acylcarnitines was observed, which has also been reported in the *Pdss2* and *Adck2* mouse models [45,52], most likely as a result of the impairment of fatty acid oxidation due to the dysfunctional Q-junction (ETFDH and GPDH, in particular). 2) A decrease in N-Ac-Glu and N-Ac-Glu-6P levels in the serum, brain and kidneys was observed. These two metabolites work in the hexosamine biosynthetic pathway and are involved in O-linked glycosylation, which has been associated with the regulation of mitochondrial function [65], and is related to neuroinflammation and myelinization [66,67]. 3) A decrease in the levels of SERPINA3, MASP1, COL1A1 and AI182371 and an increase in PZP was observed. Importantly, all these changes were normalized by treatment with either β-RA or VA. Nevertheless, the use of these potential biomarkers requires further validation in animal models, as well as in the clinic.

In conclusion, this preclinical study provides plausible mechanistic explanations and a better understanding of the phenotypic heterogeneity of CoQ deficiency syndrome, the molecular and metabolic consequences of CoQ deficiency and the integrative response to the treatment with 4-HB analogs. These data are mechanistically relevant beyond primary CoQ deficiency, since other diseases with secondary CoQ deficiency, e.g., mitochondrial diseases [[68], [69], [70]], metabolic syndrome [71] or neurodegenerative diseases [2,72], may share common pathologic mechanisms and respond to treatments with 4-HB analogs. Furthermore, our data are relevant for the potential translation of these therapeutic options into the clinic.

## 4 Methods

### 4.1 Animals and treatments

*Coq9*^+/+^ (wild-type) and *Coq9*^*R239X*^ mice were used in the study, both of which harbour a C57BL/6J genetic background. The *Coq9*^*R239X*^ mouse model (MGI: 5473628) was previously generated and characterized [13,14,51]. All animal manipulations were performed according to a protocol approved by the Institutional Animal Care and Use Committee of the University of Granada (procedures numbers February 18, 2019/016 18 February and September 16, 2019/153 September 16, 2019) and were in accordance with the European Convention for the Protection of Vertebrate Animals used for Experimental and Other Scientific Purposes (CETS #123) and the Spanish law (R.D. 53/2013). Mice were housed in the Animal Facility of the University of Granada under an SPF zone with lights on at 7:00 a.m. and off at 7:00 p.m. Mice had unlimited access to water and rodent chow (SAFE® 150, which provides 21%, 12.6% and 66.4% energy from proteins, lipids and nitrogen-free extracts, respectively). Unless stated otherwise, the analytical experiments were completed in animals at 3 months of age.

Vanillic acid (VA) and β‐resorcylic acid (β‐RA) (Merck Life Science S.L.U, Madrid, Spain) were given to the mice in the chow at a concentration of 1% (w/w). β‐RA at 1% was previously reported as therapeutically successful in *Coq9*^*R239X*^ mice [15]*.* A control group with vehicle at the same dose was studied. Mice began receiving the assigned treatments at 1 month of age (except in the experiment represented in Fig. 1B, in which the animals started the treatments at 3 months of age) and the analyses were performed at the age indicated for each case. Animals were randomly assigned to experimental groups. Data were randomly collected and processed.

The body weights were recorded once a month. The motor coordination was assessed at different months of age using the rotarod test by recording the length of time that mice could remain on the rod (“latency to fall”). Rotarod parameters were set at beginning speed of 0 rpm with an acceleration rate of 0.13 rpm/s and a maximum speed of 40 rpm.

### 4.2 Cell culture and pharmacological treatment

Mouse embryonic fibroblasts (MEFs) from *Coq9*^*+/+*^ and *Coq9*^*R239X*^ mice were grown in high glucose DMEM-GlutaMAX medium supplemented with10% FBS, 1% MEM non-essential amino acids and 1% antibiotics/antimycotic in a humidified atmosphere of 5% CO_2_ at 37 °C. MEFs were treated with different concentrations of vanillic acid (1 mM, 500 μM, 250 μM, 50 μM, 5 μM and 0.5 μM) during 7 days. Vanillic acid was dissolved in 4% DMSO, giving a final concentration of DMSO in cell culture of 0.04%. After treatment, cells were collected and analyzed. A control group with vehicle at the same dose was studied.

### 4.3 Histology and immunohistochemistry

After cervical dislocation, brains were isolated and embedded in paraffin. Sagittal sections (4 μm) were mounted on glass slides for hematoxylin-eosin staining [22], Masson's trichrome and immunohistochemistry studies. Three consecutive sections corresponding to Fig. 104 of the mouse brain atlas [73] were selected for analysis by an examiner blinded to the different experimental groups. To identify neurons (NeuN, 1:300, Merck Milipore), astrocytes (GFAP, 1:500 Sigma Aldrich) and microglial activation (Iba1, 1:500, Wako), immunohistochemistry was performed. Briefly, after deparaffination, sections were boiled using 0.1 M sodium citrate buffer at pH 6, heating at 90C in a water bath for 40 min. After numerous washes in phosphate-buffered saline (PBS, 0.1 M, 0.02% Triton-X100), sections were incubated with the primary antibody at 4°C overnight. Then, secondary antibodies conjugated with AlexaFluor 488 or 594 were used at 37 °C during 2h. Finally, the slides were mounted with ProLongTM Gold Andifade Mountant with DAPI (Invitrogen). Visualization and photography of the samples was carried out with an epifluorescence microscope (Nikon Ni–U). By using the ImageJ software (NH, Bethesda, USA), number of cells, morphology and fluorescence intensity were analyzed in cortex, brainstem and cerebellum.

### 4.4 Liquid chromatography – mass spectrometry (LC-MS) – based metabolomics

For metabolites extraction, 10 μl of serum was diluted in 1 ml lysis buffer composed of methanol/acetonitrile/H_2_O (2:2:1) and shook for 10 min at 4°C before centrifugation 15 min at full speed and 4°C. For brain and kidney samples, 35–50 mg of tissue was ground in a mortar under liquid nitrogen, and metabolites were extracted by adding 500 μl lysis buffer and shaking for 20 min before centrifugation. The supernatants were collected for LC-MS analysis. The LC-MS analysis procedure and parameters were used as described before (Zaal et al., 2017). LC-MS analysis was performed on an Exactive mass spectrometer (Thermo Scientific) coupled with a Dionex Ultimate 3000 autosampler and pump (Thermo Scientific). The MS operated in polarity-switching mode with spray voltages of 4.5 kV and −3.5 kV. Metabolites were separated using a Sequant ZIC-pHILIC column (2.1 x 150 mm, 5 μm, guard column 2.1 x 20 mm, 5 μm; Merck) with elution buffers acetonitrile and eluent A (20 mM (NH_4_)_2_CO_3_, 0.1% NH_4_OH in ULC/MS grade water (Biosolve)). The flow rate was set at 150 μl/min and the gradient ran from 20% A to 60% A in 20 min, followed by a wash at 80% and re-equilibration at 20% A. Metabolites were identified and quantified using TraceFinder software (Thermo Scientific). Metabolites were identified based on exact mass within 5 ppm and further validated by concordance with retention times of standards. The peak areas of the identified metabolites were in their respective linear range of detection. Peak intensities were additionally normalized based on the total peak intensity of the total metabolites in order to correct for technical variations during mass spectrometry analysis.

### 4.5 Transcriptome analysis by RNA-Seq

The RNeasy Lipid Tissue Mini Kit (Qiagen) was used to extract total RNAs from the brainstem and kidneys of five animals in each experimental group. The RNAs were precipitated and their quality and quantity assessed using an Agilent Bioanalyzer 2100 and an RNA 6000 chip (Agilent Technologies). The cDNA libraries were then constructed using the TruSeq RNA Sample Prep Kit v2 (Illumina, Inc.) and their quality checked using an Agilent Bioanalyzer 2100 and a DNA 1000 chip (Agilent Technologies). The libraries were Paired End sequenced in a HiSeq 4000 system (Illumina, Inc.) at Macrogen Inc. We aimed for 4–5 Giga Bases outcome per sample. The quality of the resulting sequencing reads was assessed using FastQC. The GRCm38.p5 fasta and gtf files of the reference mouse genome were downloaded from the Ensembl database and indexed using the bwtsw option of BWA (Linden et al., 2012). BWA, combined with *xa2multi.pl* and SAMtools (Li et al., 2009), was also used for aligning the sequencing reads against the reference genome, and HTSeq was used for counting the number of reads aligned to each genomic locus (Mishanina et al., 2015). The alignments and counting were carried out in our local server following the protocols as described [74].

After elimination of the genomic loci that aligned to <5 reads in <5 samples and normalization of the read counts by library size, the differential gene expression was detected [15], using the Generalized Linear Model (glmLRT option) statistic in EdgeR. We used a 0.05 *P*‐level threshold after False Discovery Rate correction for type I error. The heatmap figure was made using the heatmap function in R (https://www.r-project.org/). Annotation of the differentially expressed genes was obtained from the Mouse Genome Informatics (http://www.informatics.jax.org/).

The transcripts that filled the inclusion criteria were then subjected to gene classification, using a databank based on hand-curated literature mining for specific protein–protein interactions and regulatory networks (Ingenuity Pathway Analysis (IPA); Ingenuity Systems, Redwood City, CA, USA). The general canonical pathways, biological functions and diseases implicated for the significantly changed transcripts by Ingenuity Pathway Analysis were evaluated and *P*-values <0.01 were considered significant. Furthermore, specific functional networks based on published knowledge on protein–protein interactions and regulatory networks were constructed using Ingenuity Pathway Analysis (Raimundo et al., 2009).

### 4.6 Mitochondrial proteomics analysis

Both *Coq9*^+/+^ mice and *Coq9* ^+/+^ mice under 1% of VA and β‐RA supplementation were sacrificed, and the brain and kidneys were removed and washed in saline buffer. The tissues were chopped with scissors in 3 ml HEENK (10 mM HEPES, 1 mM EDTA, 1 mM EGTA, 10 mM NaCl, 150 mM KCl, pH 7.1, 300 mOsm/l) containing 1 mM phenylmethanesulfonyl fluoride (from 0.1 M stock in isopropanol) and 1x protease inhibitor cocktail (Pierce). The tissues were homogenized with a 3 ml dounce homogenizer (5 passes of a tight-fitting Teflon piston). Each homogenate obtained was rapidly subjected to standard differential centrifugation methods until the mitochondrial pellet was obtained as previously described [75]. Briefly, the homogenate was centrifuged at 600 *g* for 5 min at 4 °C (twice), and the resulting supernatant was centrifuged at 9000 *g* for 5 min at 4 °C. The final pellet, corresponding to a crude mitochondrial fraction, was resuspended in 500 μl of HEENK medium without PMSF or protease inhibitor [75]. Protein concentration was determined (using Bradford dye (BIO-RAD) and a Shimadzu spectrophotometer, resulting in approximately 3 mg protein for renal mitochondria and 1.5 mg for cerebral mitochondria. To verify the content of the mitochondrial fraction, Complex IV activity was determined by optical absorption of the difference spectrum at 550 nm, as previously described [51].

The purified mitochondria were spun down to remove the previous buffer, and lysis buffer (1% sodium deoxycholate SDC in 100 mM tris at pH 8.5) was added to the pellets. Samples were boiled for 5 min at 99°C to denature all the proteins and then sonicated by microtip probe sonication (Hielscher UP100H Lab Homogenizer) for 2 min with pulses of 1s on and 1s off at 80% amplitude. Protein concentration was estimated by BCA assay and 200 μg were taken of each sample. 10 mM tris (2-carboxyethyl) phosphine and 40 mM chloroacetamide (final concentration) at 56 °C were added to each of these 200 μg samples for 10 min to reduce and alkylate disulfide bridges. After this step, samples were digested with LysC (Wako) in an enzyme/protein ratio of 1:100 (w/w) for 1 h, followed by a trypsin digest (Promega) 1:50 (w/w) overnight. Protease activity was quenched with trifluoroacetic acid (TFA) to a final pH of ∼2. Samples were then centrifuged at 5,000*g* for 10 min to eliminate the insoluble SDC, and loaded on an OASIS HLB (Waters) 96-well plate. Samples were washed with 0.1% TFA, eluted with a 50/50 ACN and 0.1% TFA, dried by SpeedVac (Eppendorf, Germany), and resuspended in 2% formic acid prior to MS analysis. 5 μg were taken from each sample and pooled in order to be used for quality control for MS (1 QC was analyzed every 12 samples) and to be fractionated at high-pH for the Match between runs.

Plasma was extracted in EDTA tubes and the most abundant proteins were removed using the Multiple Affinity Removal Spin Cartridge Mouse 3 (Agilent, 5188–5289). The cells were lysed by the addition of 5 μl of lysis buffer (1% SDC, 10 mM TCEP, 100 mM TRIS, 40 mM chloroacetamide at pH 8.5) for up to 20 μg of protein. Samples were boiled for 5 min at 95°C to denature all the proteins and then sonicated for 15 min with pulses of 30s on and 30s off (Bioruptor, model ACD-200, Diagenode). The pelleted cell debris was discarded after centrifugation at full speed for 10 min and the supernatant was digested overnight at 37°C with LysC (Wako) in an enzyme/protein ratio of 1:75 (w/w) and with trypsin (Sigma) at 1:50 (w/w). Protease activity was quenched with formic acid (FA) to a final concentration of 2% FA. Samples were then centrifuged at 20,000*g* for 20 min to eliminate the insoluble SDC, and loaded on an OASIS HLB (Waters) 96-well plate. Samples were washed with 0.1% FA, eluted with a 50/50 ACN and 0.1% FA, dried by SpeedVac (Eppendorf, Germany), and resuspended in 2% formic acid prior to MS analysis.

All samples with the QC and 7 high-pH fractions were acquired using an UHPLC 1290 system (Agilent Technologies; Santa Clara, USA) coupled on-line to an Q Exactive HF mass spectrometer (Thermo Scientific; Bremen, Germany). Peptides were first trapped (Dr. Maisch Reprosil C18, 3 μm, 2 cm × 100 μm) prior to separation on an analytical column (Agilent Poroshell EC-C18, 2.7 μm, 50 cm × 75 μm). Trapping was performed for 5 min in solvent A (0.1% v/v formic acid in water), and the gradient was as follows: 13%–44% solvent B (0.1% v/v formic acid in 80% v/v ACN) over 95 min, 44–100% B over 2 min, then the column was cleaned for 4 min and equilibrated for 10 min (flow: 200 nL/min). The mass spectrometer was operated in a data-dependent mode. Full-scan MS spectra from *m*/*z* 375–1600 Th were acquired in the Orbitrap at a resolution of 60,000 after accumulation to a target value of 3E6 with a maximum injection time of 20 ms. The 15 most abundant ions were fragmented with a dynamic exclusion of 24 s. HCD fragmentation spectra (MS/MS) were acquired in the Orbitrap at a resolution of 30,000 after accumulation to a target value of 1E5 with an isolation window of 1.4 Th and maximum injection time 50 ms.

All raw files were analyzed by MaxQuant v1.6.10 software [76] using the integrated Andromeda Search engine and searched against the mouse UniProt Reference Proteome (November 2019 release with 55,412 protein sequences) with common contaminants. Trypsin was specified as the enzyme, allowing up to two missed cleavages. Carbamidomethylation of cysteine was specified as fixed modification and protein N-terminal acetylation, oxidation of methionine, and deamidation of asparagine were considered variable modifications. We used all the default settings and activated “Match between runs” (time window of 0.7 min and alignment time window of 20 min) and LFQ with standard parameters. The files generated by MaxQuant were opened in Perseus for the preliminary data analysis: the LFQ data were first log2 transformed, then identifications present in at least N (3/5) biological replicates were kept for further analysis; missing values were then imputed using the standard settings of Perseus. Ingenuity Pathway Analysis (IPA) analysis was used to identify changes in metabolic canonical pathways and their z-score predictions [77].

### 4.7 Sample preparation and Western blot analysis in tissues and cells

For western blot analyses, a glass-Teflon homogenizer was used to homogenize mouse kidneys, liver and brain samples at 1100 rpm in T-PER® buffer (Thermo Scientific) with protease and phosphatase inhibitor cocktail (Pierce). Homogenates were sonicated and centrifuged at 1000g for 5 min at 4 °C, and the resultant supernatants were used for western blot analyses. For western blot analyses in cells, the pellets containing the cells were re-suspended in RIPA buffer with protease inhibitor cocktail. About 40 μg of protein from the sample extracts were electrophoresed in 12% Mini-PROTEAN TGXTM precast gels (Bio-Rad) using the electrophoresis system mini-PROTEAN Tetra Cell (Bio-Rad). Proteins were transferred onto PVDF 0.45-μm membranes using a *Trans*-blot Cell (Bio-Rad) and probed with target antibodies. Protein–antibody interactions were detected using peroxidase-conjugated horse anti-mouse, anti-rabbit, or anti-goat IgG antibodies and Amersham ECLTM Prime Western Blotting Detection Reagent (GE Healthcare, Buckinghamshire, UK). Band quantification was carried out using an Image Station 2000R (Kodak, Spain) and a Kodak 1D 3.6 software. Protein band intensity was normalized to VDAC1 for mitochondrial proteins and to β-actin for cytosolic proteins. The data were expressed in terms of percent relative to wild-type mice or control cells.

The following primary antibodies were used: anti-COQ2 (Origene, TA341982), anti-COQ4 (Proteintech, 16654-1-AP), anti-COQ5 (Proteintech, 17453-AP), anti-COQ7 (Proteintech, 15083-1-AP), anti-PRODH (Cell Signaling, #22980), anti-OPA1 (Cell Signaling, #27733), anti-CRYAB (Cell Signaling, #15808), anti-DMGDH (Cell Signaling, #24813), anti-SERPINA1 (Cell Signaling, #16382), anti-SERPINA3 (Cell Signaling, #12192), anti-VDAC1 (Abcam, ab14734) and anti-ACTIN (Invitrogen, #MA5-15739-HRP).

### 4.8 Gene expression analyses

Total cellular RNA from tissue was extracted following the TRI Reagent Solution from Applied Biosystems and electrophoresed in agarose 1.5% to check RNA integrity. Total RNA was quantified by optical density at 260/280 nm and was used to generate cDNA with High-Capacity cDNA Reverse Transcription Kit (Applied Biosystems). Amplification was performed with quantitative real-time PCR, by standard curve method, with specific Taqman probes (from Applied Biosystems) for the targeted gene mouse *Coq2* (Mm01203260_m1), *Coq4* (Mm00618552_m1), *Coq5* (Mm00518239_m1), *Coq7* (Mm00501588_m1), *Ccl2* (Mm00441242_m1) and *Cxcl10* (Mm00445235_m1) and the mouse *Hprt* probe as a standard loading control (Mm01545399_m1).

### 4.9 Quantification of CoQ_9_ and DMQ_9_ levels in tissues and cells

After lipid extraction from homogenized tissues [13] or cells [78], CoQ_9_ and DMQ_9_ levels were determined via reversed‐phase HPLC coupled to electrochemical detection, as previously described. Since DMQ_9_ is not commercially available, its identification is based in the mass of the molecule and its retention time, as previously shown [13]. Then, a standard curve of CoQ_9_ was used for a quantitative estimation. The results were expressed in ng CoQ/mg protein.

### 4.10 CoQ‐dependent respiratory chain activities

Coenzyme Q‐dependent respiratory chain activities were measured in tissue samples of brain, kidneys and liver. Tissue samples were homogenized in CPT medium (0.05 M Tris-HCl, 0.15 M KCl, pH 7.5) at 1100 rpm in a glass–Teflon homogenizer. Homogenates were sonicated and centrifuged at 600 g for 20 min at 4 °C, and the supernatants obtained were used to measure CoQ-dependent respiratory chain activities (CI + III and CII + III) as previously described [9]. The results were expressed in nmol reduced cyt *c*/min/mg prot and nmol reduced cyt *c*/min/citrate synthase activity. The citrate synthase activity was measured, with the previously obtained supernatants, at 30°C in the presence of 0.3 mM acetyl-CoA and 0.1 mM DTNB (5,5-dithio-bis-(2-nitrobenzoic acid)). The reaction was initiated by the addition of 0.5 mM oxalacetate and the absorbance was monitored at 412 nm.

### 4.11 Mitochondrial respiration

Mitochondrial isolation from the brain and the kidneys was performed as previously described [15,79]. Mitochondrial respiration was measured using an XF^e24^ Extracellular Flux Analyzer (Seahorse Bioscience) [15,79,80]. Mitochondria were first diluted in cold MAS 1X for plating (3.5 μg/in brain; 2 μg/well in kidney). Next, 50 μl of mitochondrial suspension was delivered to each well (except for background correction wells) while the plate was on ice. The plate was then centrifuged at 2,000*g* for 10 min at 4°C. After centrifugation, 450 μl of MAS 1X + substrate (10 mM succinate, 2 mM malate, 2 mM glutamate and 10 mM pyruvate) was added to each well. Respiration by the mitochondria was sequentially measured in a coupled state with the substrate present (basal respiration or State 2) followed by State 3° (phosphorylating respiration, in the presence of ADP and substrates). State 4 (non-phosphorylating or resting respiration) was measured after addition of oligomycin when all ADP was consumed, and then maximal uncoupler-stimulated respiration was measured by FCCP (State 3u). Injections were as follows: port A, 50 μl of 40 mM ADP (4 mM final); port B, 55 μl of 30 μg/ml oligomycin (3 μg/ml final); port C, 60 μl of 40 μM FCCP (4 μM final); and port D, 65 μl of 40 μM antimycin A (4 μM final). All data were expressed in pmol/min/mg protein.

### 4.12 Quantification of VA levels in mice tissues

Tissues from mice were homogenized in water. The homogenate samples were then treated with a solution of methanol/water (80:20, v/v), shook for 1 min, sonicated for 15 min and then centrifuged at 5,000*g* for 25 min at 4°C (Borges et al., 2017).

The supernatants were analyzed using a Thermo Scientific™ UltiMate™ 3000 UHPLC system (Waltham, Massachusetts, United States), consisting of an UltiMate™ 3000 UHPLC RS binary pump and an UltiMate™ 3000 UHPLC sample manager coupled to a Thermo Scientific™ Q Exactive™ Focus Hybrid Quadrupole-Orbitrap™ detector of mass spectrometer (MS/MS) with an electrospray ionization in negative mode (Waltham, Massachusetts, United States). The analytical separation column was a Hypersil GOLD™ C18, 3 μm, 4.6 × 150 mm column (Thermo Scientific™) and the flow rate was 0.6 ml/min. The mobile phase consisted of two solutions: eluent A (H_2_O + 0.1% Formic acid, MS grade, Thermo Scientific™) and eluent B (acetonitrile + 0.1% Formic acid, MS grade, Thermo Scientific™). Samples were eluted over 30 min with a gradient as follow: 0 min, 95% eluent A; 0–25 min, 70% eluent A; 25–25.1 min, 95% eluent A; 25.1–30 min, 95% eluent A. Capillary and auxiliary gas temperatures were set at 275 and 450 °C, respectively. Sheath gas flow rate used was at 55 arbitrary units, the auxiliary gas flow rate used was at 15 arbitrary units, and sweep gas flow was used at 3 arbitrary units. Mass spectrometry analyses were carried out in full scan mode between 110 and 190 uma. To quantify the levels of VA, we used a standard curve with the compound at a concentration of 100 ng/ml, 10 ng/ml and 1 ng/ml.

### 4.13 Statistical analysis

The number of animals in each group were calculated in order to detect gross ∼60% changes in the biomarker measurements (based upon alpha=0.05 and power of beta=0.8). We used the application available at http://www.biomath.info/power/index.htm. Animals were genotyped and randomly assigned to experimental groups in separate cages by the technician of the animal facility. Most statistical analyses were performed using the Prism 9 scientific software. Data are expressed as the mean ± SD of five to ten experiments per group. A one-way ANOVA with a Tukey's post hoc test was used to compare the differences between the three experimental groups. Studies with two experimental groups were evaluated using unpaired Student's t-test. A P value of <0.05 was considered to be statistically significant. Survival curve was analyzed by log-rank (Mantel-Cox) and the Gehan-Breslow-Wilcoxon tests. The statistical tests used for the transcriptomics and proteomics analyses are described in their respective sections.

## Author contribution

P.G.-G led the study, developed the phenotypic and survival assay and the body weight measurements; conducted the tests to assess the mitochondrial bioenergetics, Western blot analyses, enzymatic assays, UHPLC EC and MS analysis, serum proteomics and metabolomics, tissues metabolomics, and IPA analyses; analyzed the results; designed the figures; and wrote the manuscript. M.E.D.-C. performed the morphological analyses. A.H.-G. conducted the mitochondrial proteomics experiments and contributed the phenotypic experiments. L.J.-S. performed the immunohistochemical analyses. M.B. developed and supervised the transcriptomics analyses. E.B.-C. contributed to the mitochondrial assays, the management of the mouse colony, and the phenotyping tests. G.E. contributed to the discussions. R.Z.C. supervised the proteomics experiments and analyses in mitochondria. F.V. supervised the proteomics experiments and analyses in serum. E.A.Z. supervised the metabolomics experiments and analyses. C.R.B. supervised the metabolomics experiments and analyses. A.J.R.H. supervised the proteomics analyses. L.C.L. conceived the idea for the project, supervised the experiments, and edited the manuscript. All authors have read, revised and agreed to the published version of the manuscript. The results shown in this article constituted a section of P.G.-G's doctoral thesis at the University of Granada.

## 5 Data availability

The mass spectrometry proteomics data were deposited to the ProteomeXchange (http://www.proteomexchange.org/) on December 11th, 2021. Consortium via the PRIDE partner repository with the dataset identifier PXD030303. RNA-Seq data were generated as described above. The files have been uploaded to the repository Gene Expression Omnibus. The accession number is PRJNA796310. All data can be found at https://www.ncbi.nlm.nih.gov/geo/query/acc.cgi?acc=PRJNA796310.

## Declaration of interests

The authors declare that they have no known competing financial interests or personal relationships that could have appeared to influence the work reported in this paper.
