# Supplementary material for: The Q-junction and the inflammatory response are critical pathological and therapeutic factors in CoQ deficiency
Source: Redox Biol. 2022 Jul 15;55:102403. doi: 10.1016/j.redox.2022.102403 (PMC9301574; doi:10.1016/j.redox.2022.102403)
Supplement: Multimedia component 2 [file mmc2.pdf]

Figure S5. COQ2 in MEFs of wild-type and mutant mice with and without treatment.

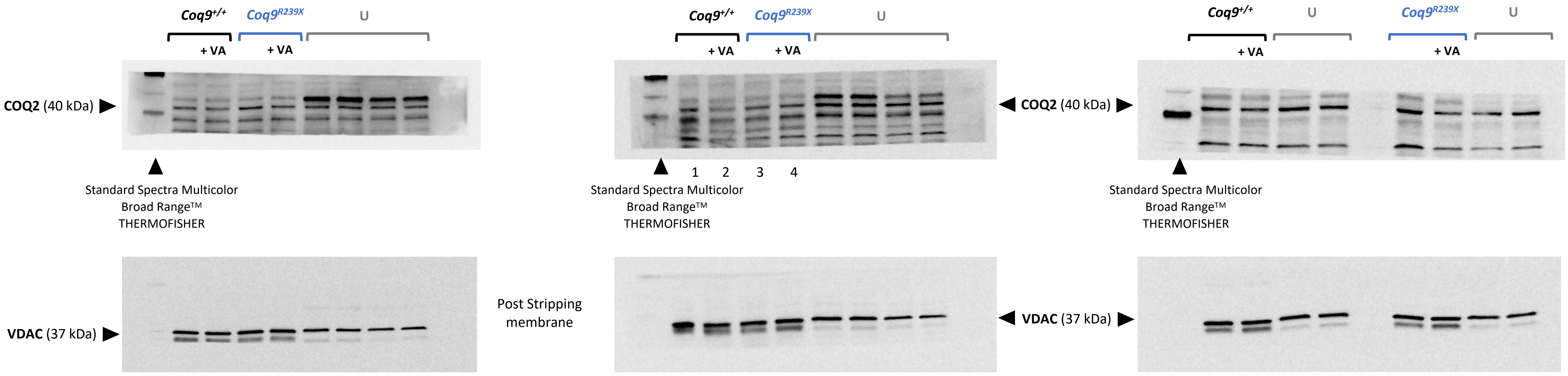

All membranes were cut before the incubation with the primary antibody.

Note: lines 1, 2, 3 and 4 are represented in Figure 3A in the main text.

U = Unrelated to this study

Figure S5. COQ4 in MEFs of wild-type and mutant mice with and without treatment.

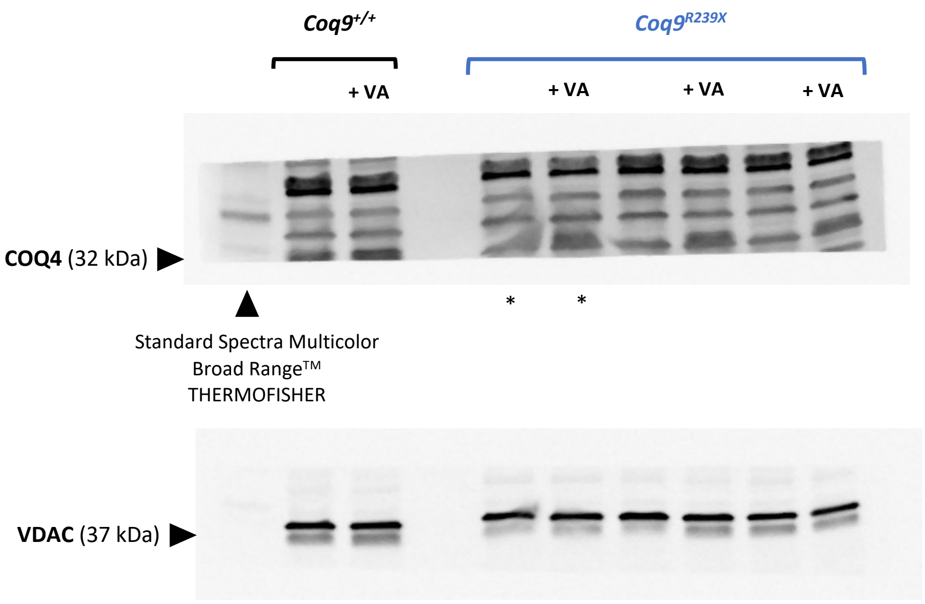

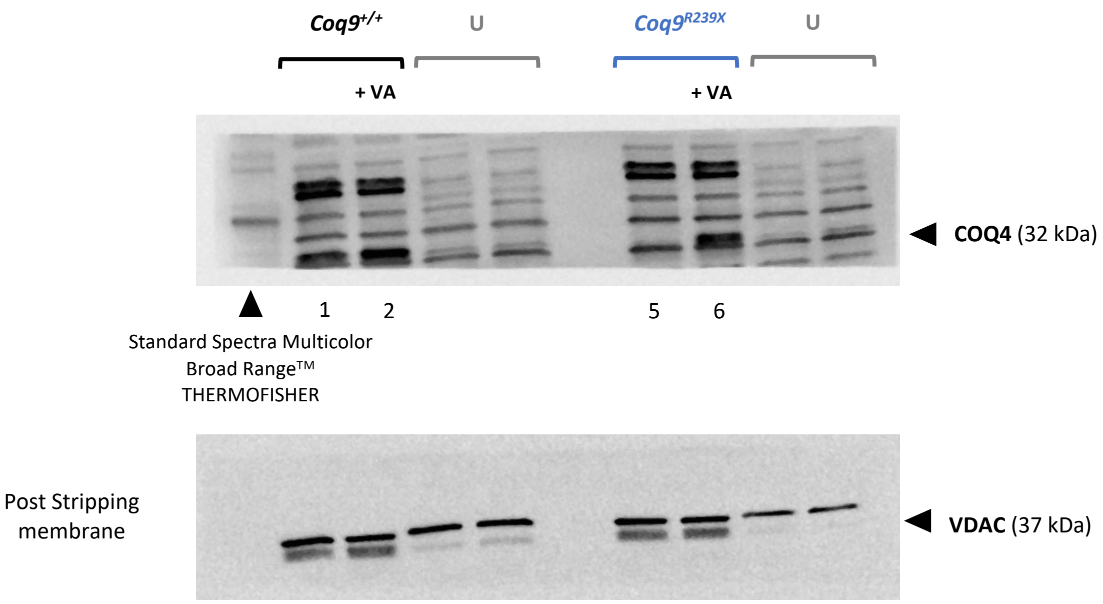

All membranes were cut before the incubation with the primary antibody.

Note: lines 1, 2, 5 and 6 are represented in Figure 3B in the main text.

U = Unrelated to this study

* Repeted samples 5 and 6.

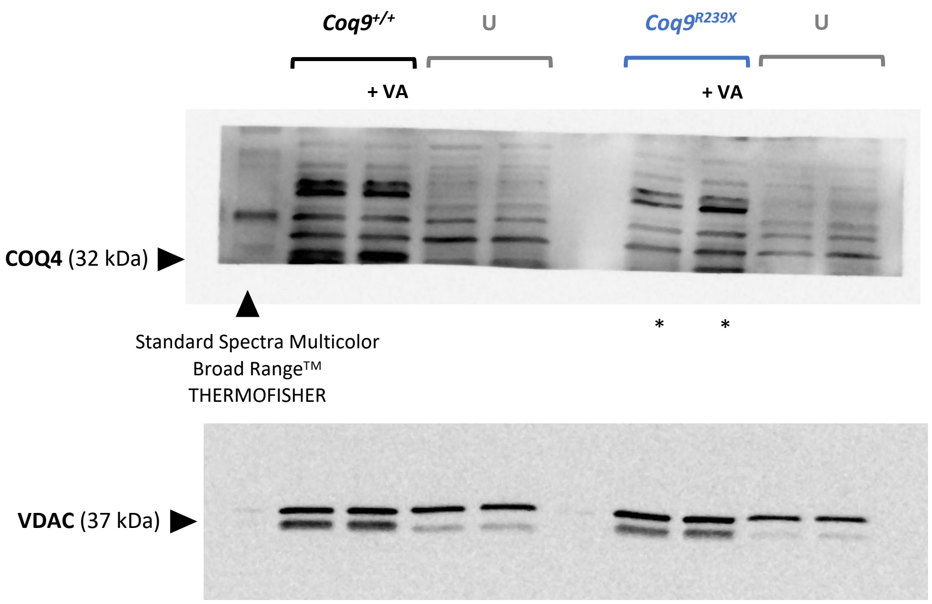

Figure S5. COQ5 in MEFs of wild-type and mutant mice with and without treatment.

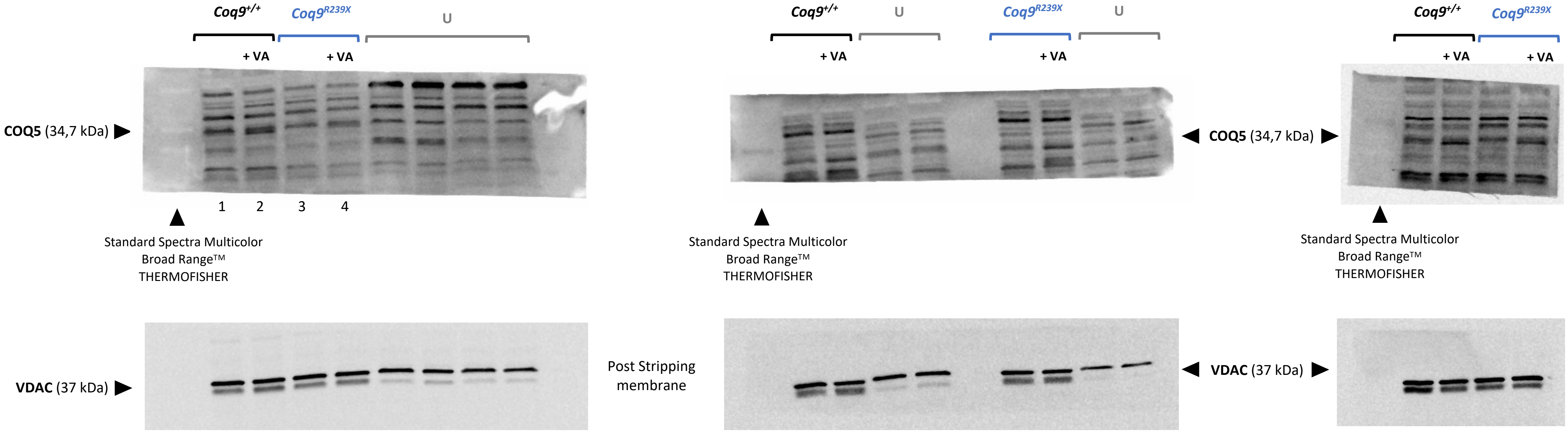

All membranes were cut before the incubation with the primary antibody.

Note: lines 1, 2, 3 and 4 are represented in Figure 3C in the main text.

U = Unrelated to this study

Figure S5. COQ7 in MEFs of wild-type and mutant mice with and without treatment.

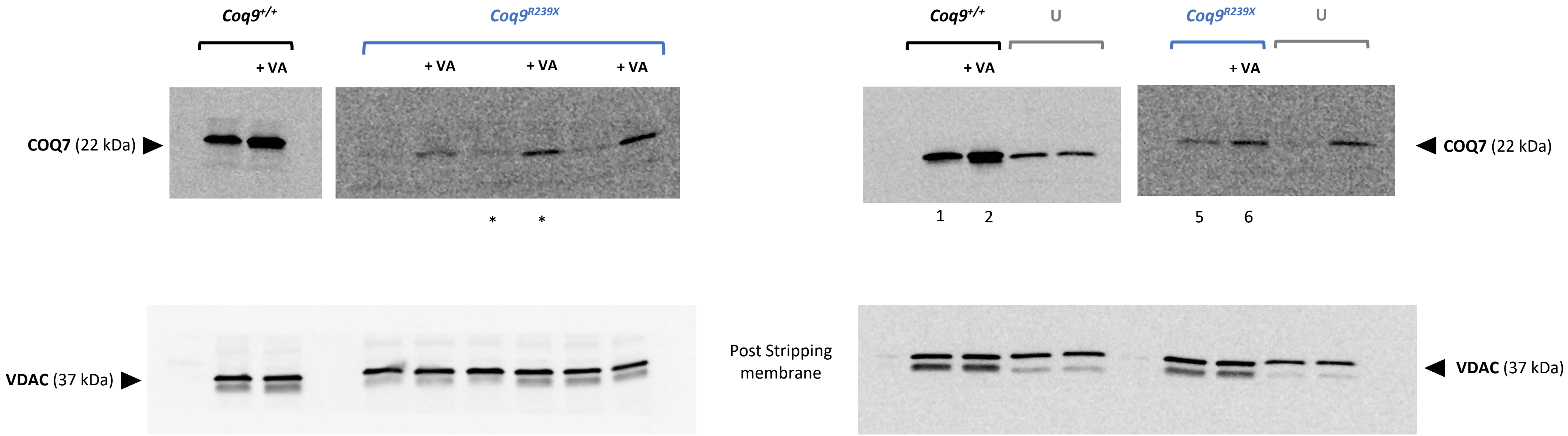

All membranes were cut before the incubation with the primary antibody.

Note: lines 1, 2, 5 and 6 are represented in Figure 3D in the main text.

U = Unrelated to this study

* Repeted samples 5 and 6.

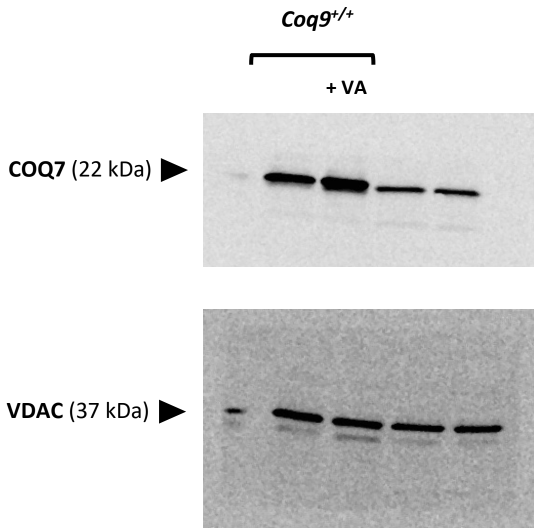

Figure S5. COQ2 in brain of wild-type and mutant mice with and without treatment.

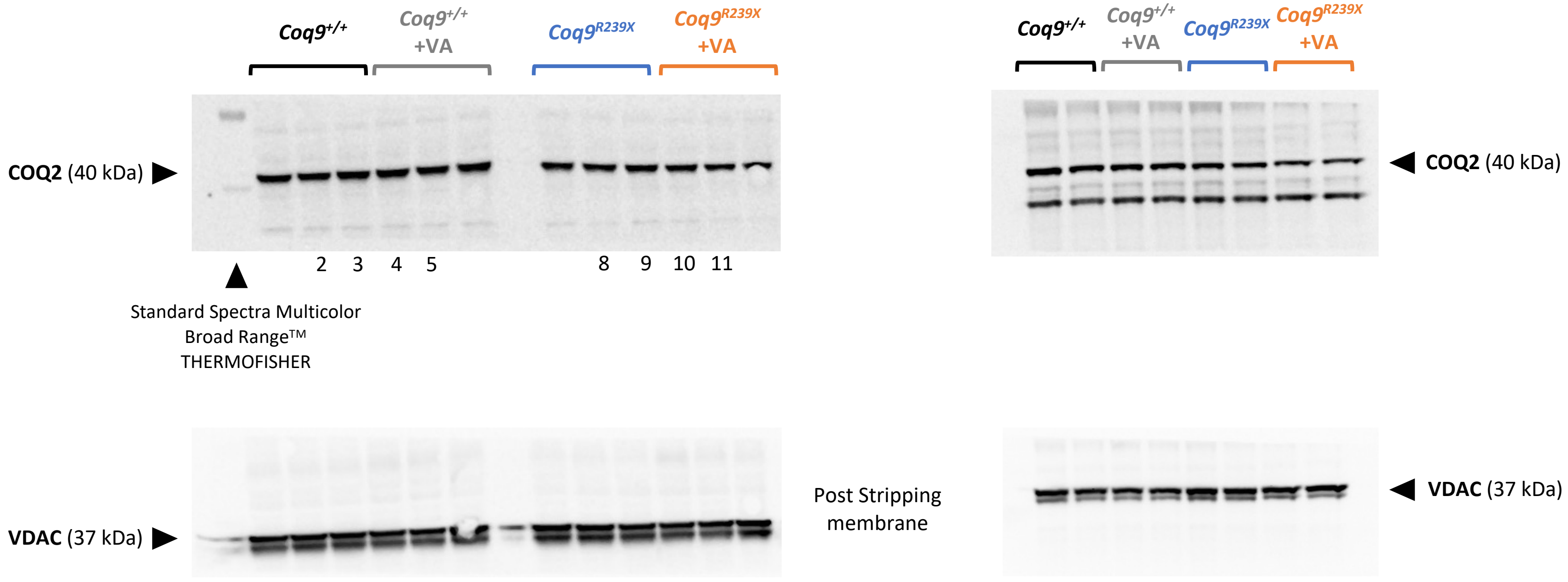

All membranes were cut before the incubation with the primary antibody.

Note: lines 2, 3, 4, 5, 8, 9, 10 and 11 are represented in Figure 3I in the main text.

Figure S5. COQ4 in brain of wild-type and mutant mice with and without treatment.

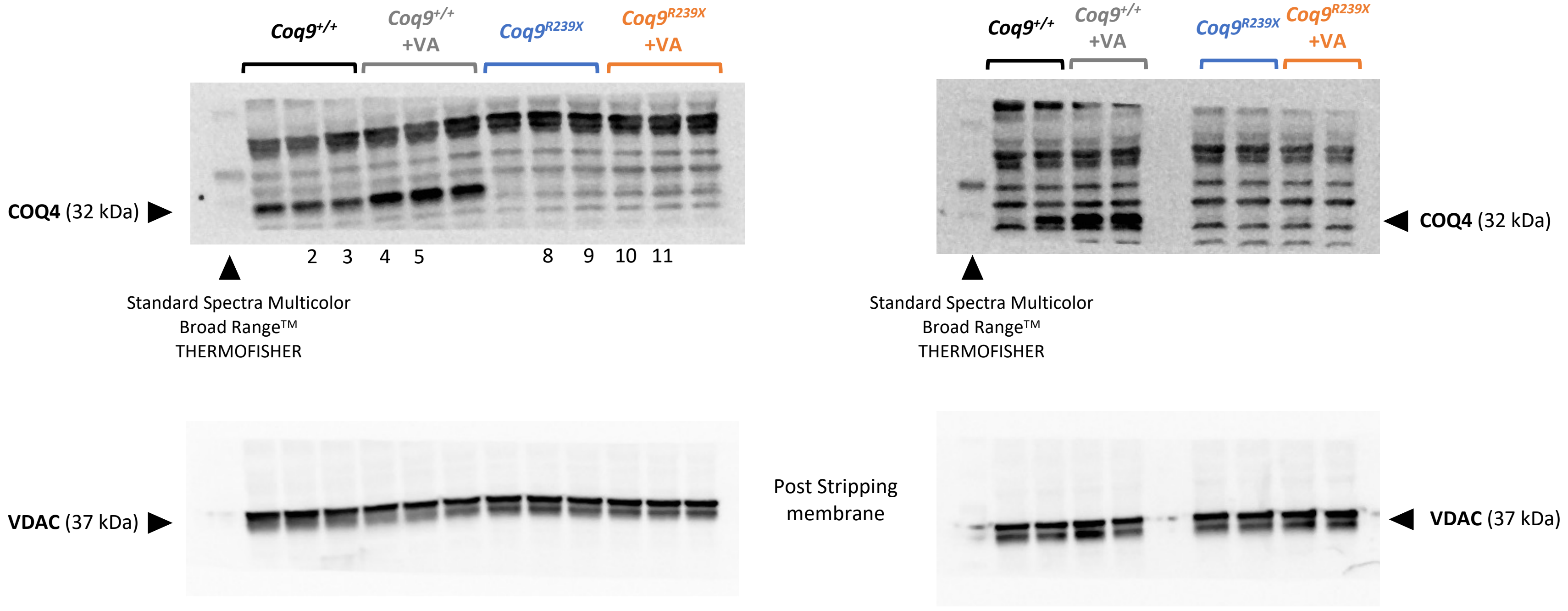

All membranes were cut before the incubation with the primary antibody.

Note: lines 2, 3, 4, 5, 8, 9, 10 and 11 are represented in Figure 3J in the main text.

Figure S5. COQ5 in brain of wild-type and mutant mice with and without treatment.

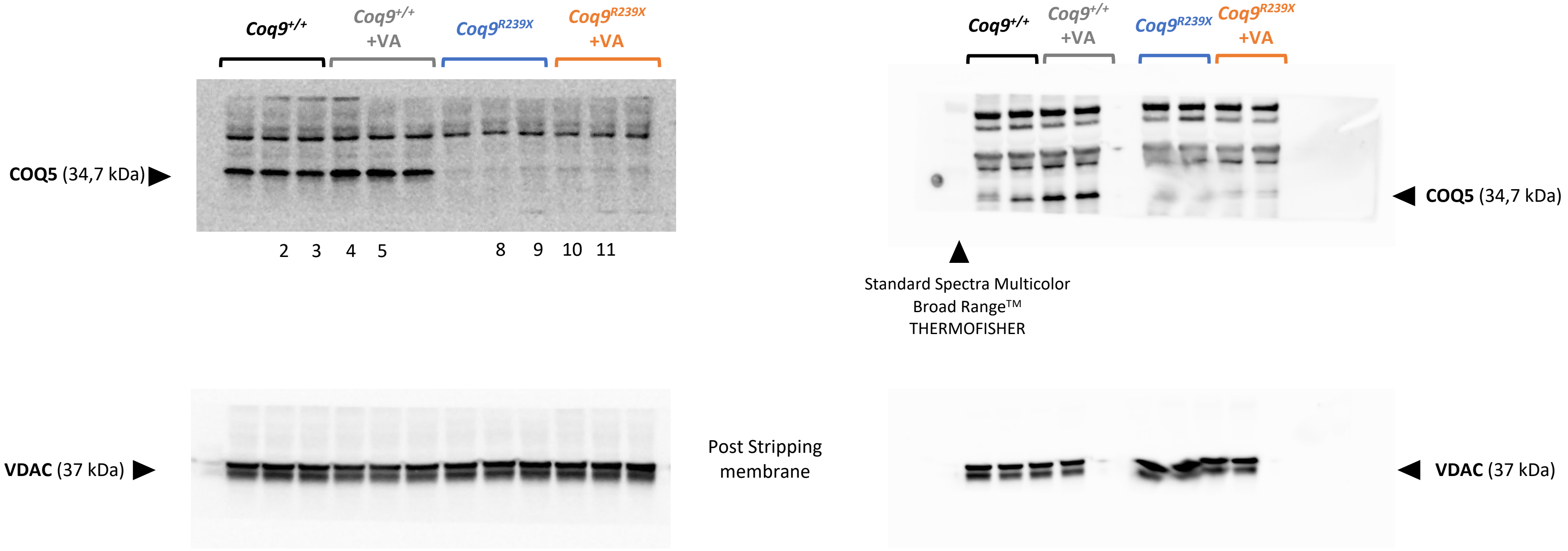

All membranes were cut before the incubation with the primary antibody.

Note: lines 2, 3, 4, 5, 8, 9, 10 and 11 are represented in Figure 3K in the main text.

Figure S5. COQ7 in brain of wild-type and mutant mice with and without treatment.

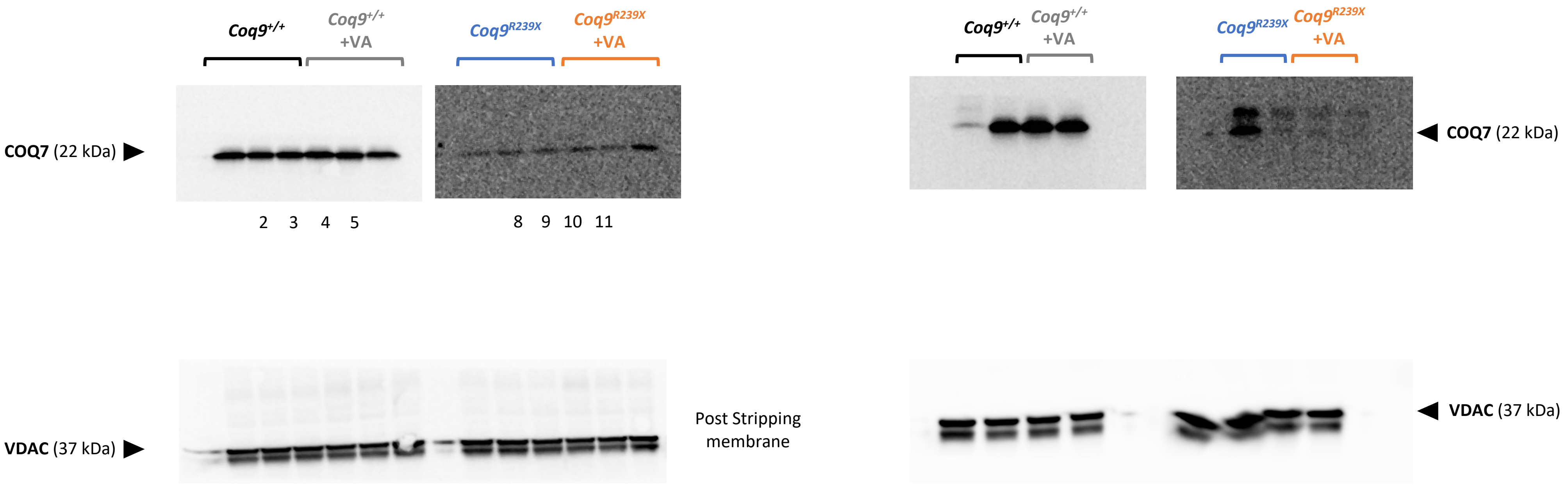

All membranes were cut before the incubation with the primary antibody.

Note: lines 2, 3, 4, 5, 8, 9, 10 and 11 are represented in Figure 3L in the main text.

Figure S5. COQ2 in kidney of wild-type and mutant mice with and without treatment.

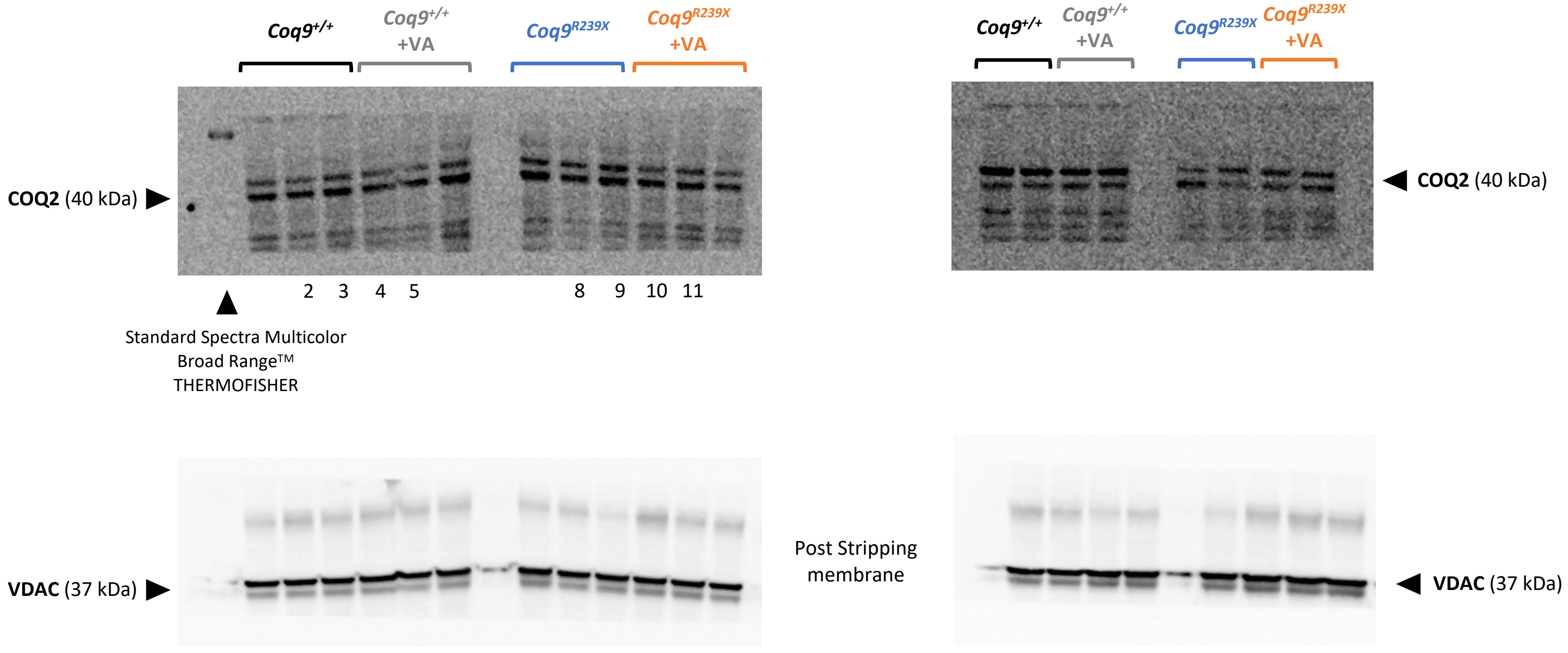

All membranes were cut before the incubation with the primary antibody.

Note: lines 2, 3, 4, 5, 8, 9, 10 and 11 are represented in Figure 3M in the main text.

Figure S5. COQ4 in kidney of wild-type and mutant mice with and without treatment.

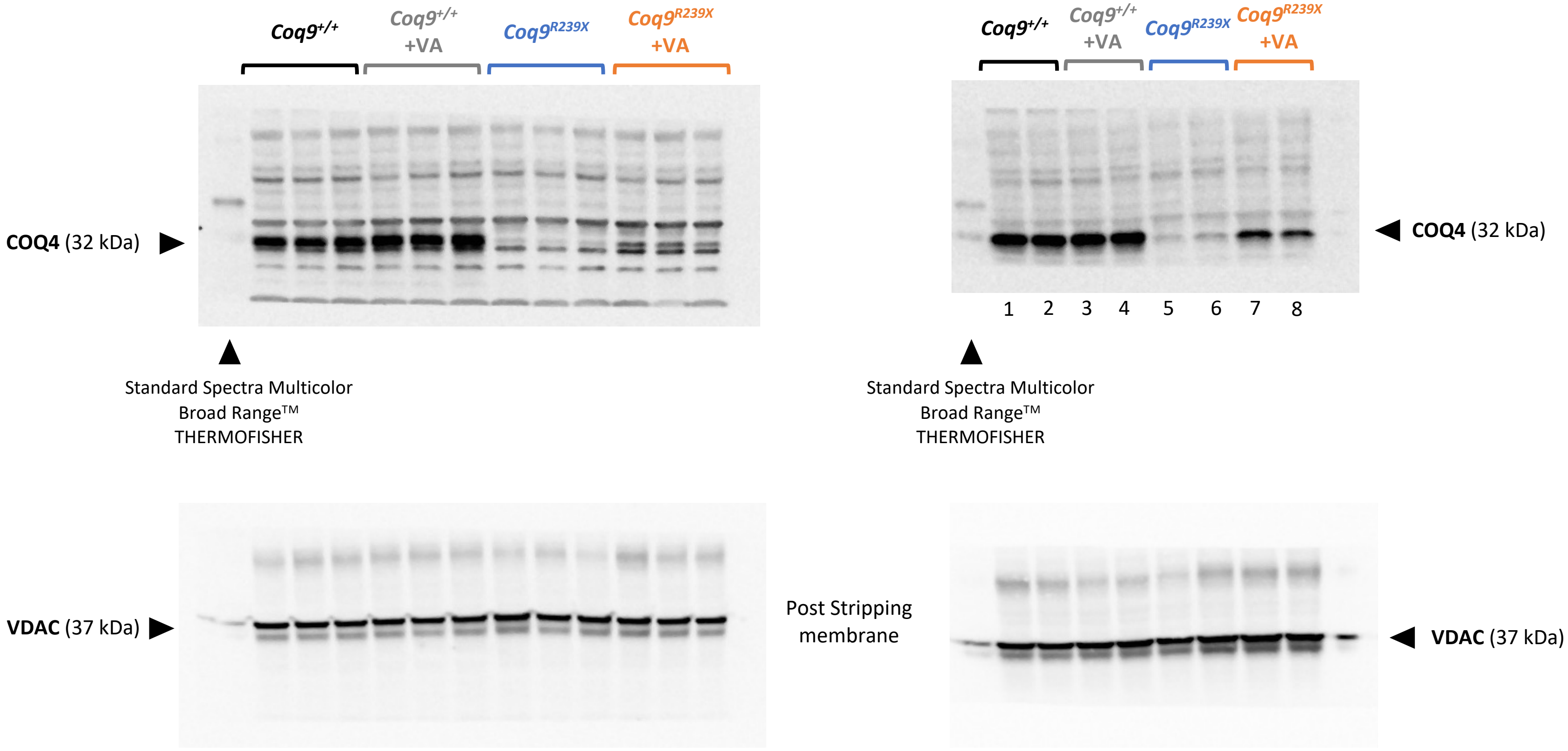

All membranes were cut before the incubation with the primary antibody.

Note: lines 1, 2, 3, 4, 5, 6, 7 and 8 are represented in Figure 3N in the main text.

Figure S5. COQ5 in kidney of wild-type and mutant mice with and without treatment.

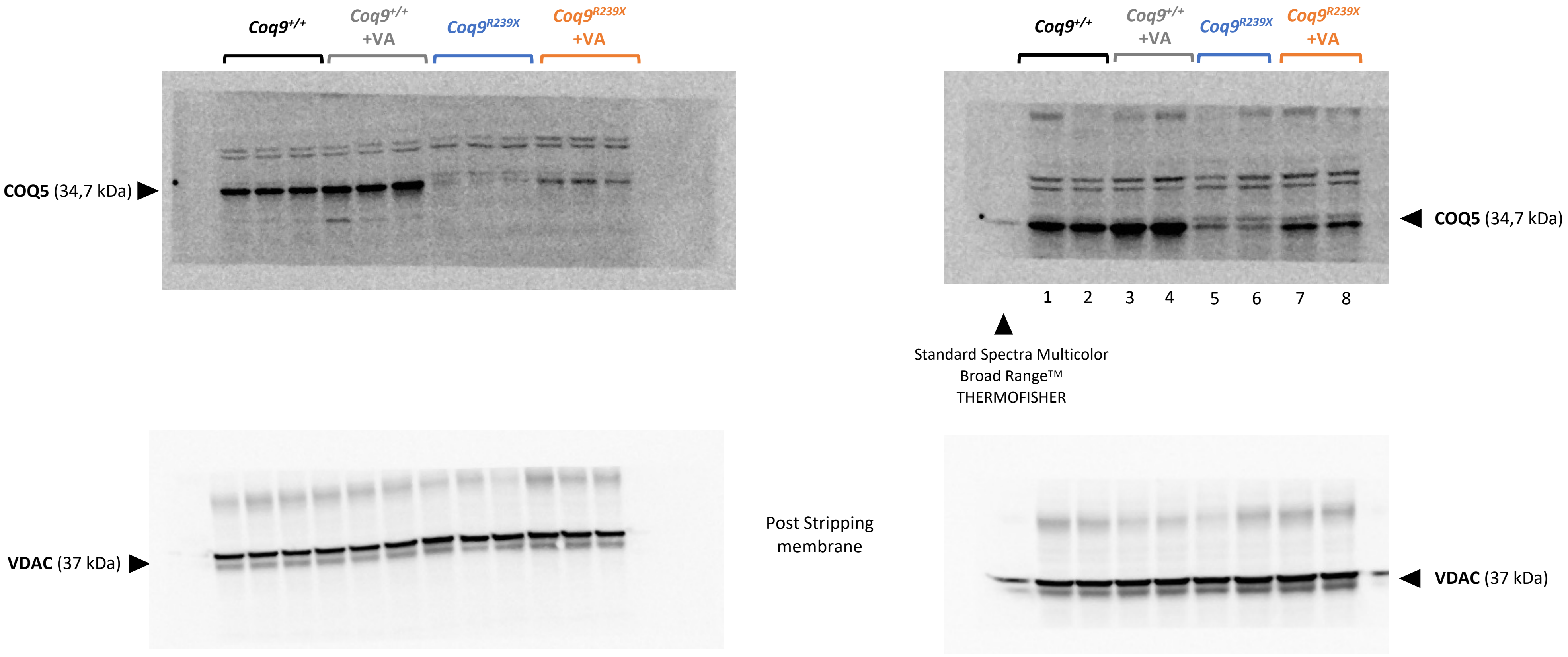

All membranes were cut before the incubation with the primary antibody.

Note: lines 1, 2, 3, 4, 5, 6, 7 and 8 are represented in Figure 3O in the main text.

Figure S5. COQ7 in kidney of wild-type and mutant mice with and without treatment.

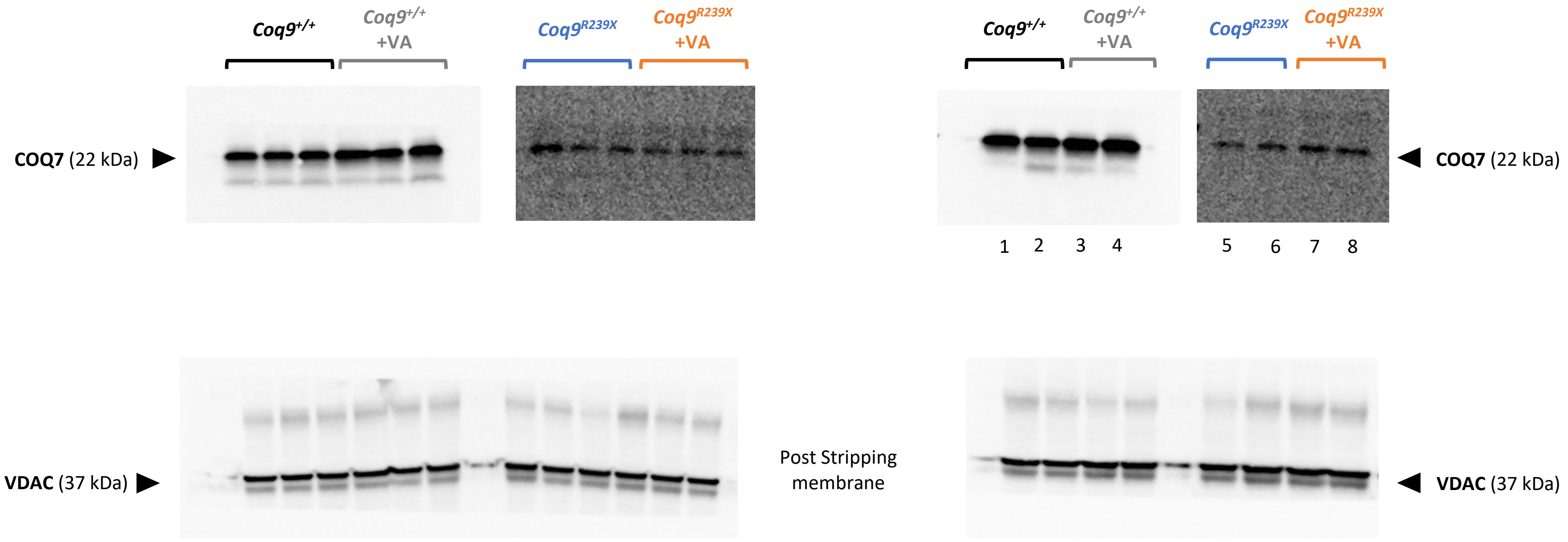

All membranes were cut before the incubation with the primary antibody.

Note: lines 1, 2, 3, 4, 5, 6, 7 and 8 are represented in Figure 3P in the main text.

Figure S5. COQ2 in liver of wild-type and mutant mice with and without treatment.

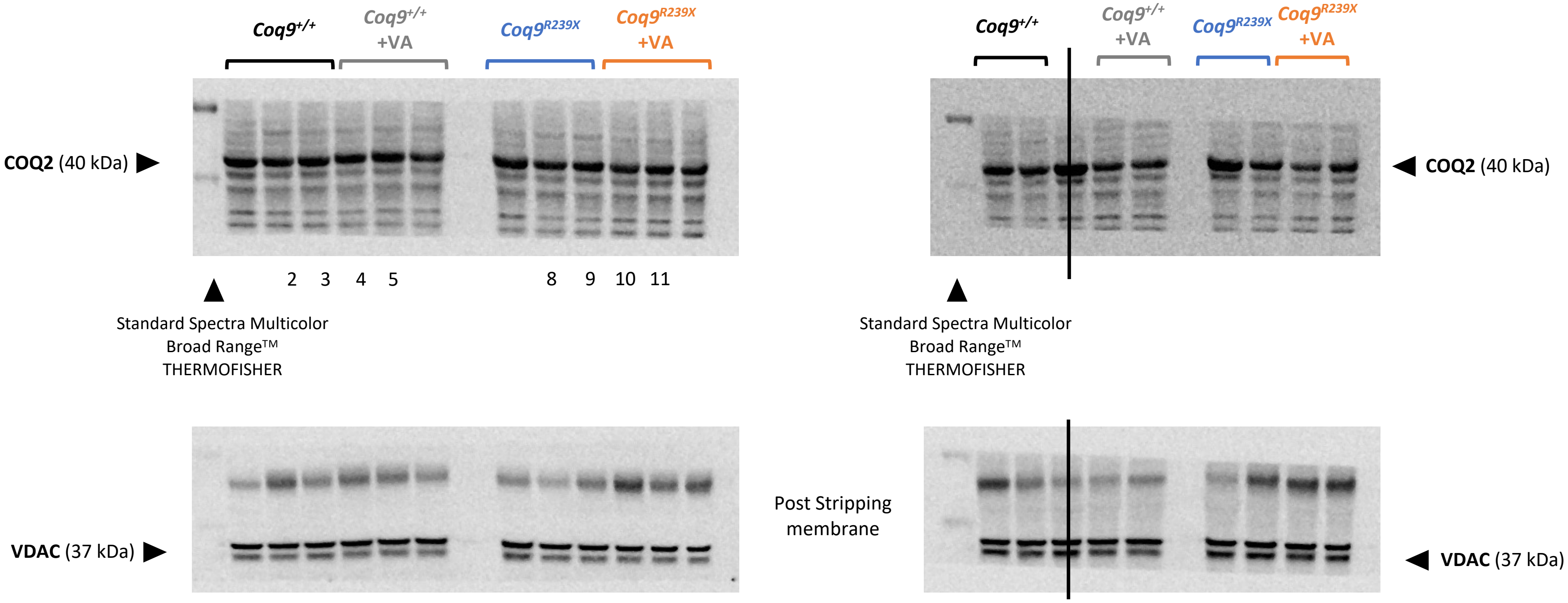

All membranes were cut before the incubation with the primary antibody.

Note: lines 2, 3, 4, 5, 8, 9, 10 and 11 are represented in Figure 3Q in the main text.

Figure S5. COQ4 in liver of wild-type and mutant mice with and without treatment.

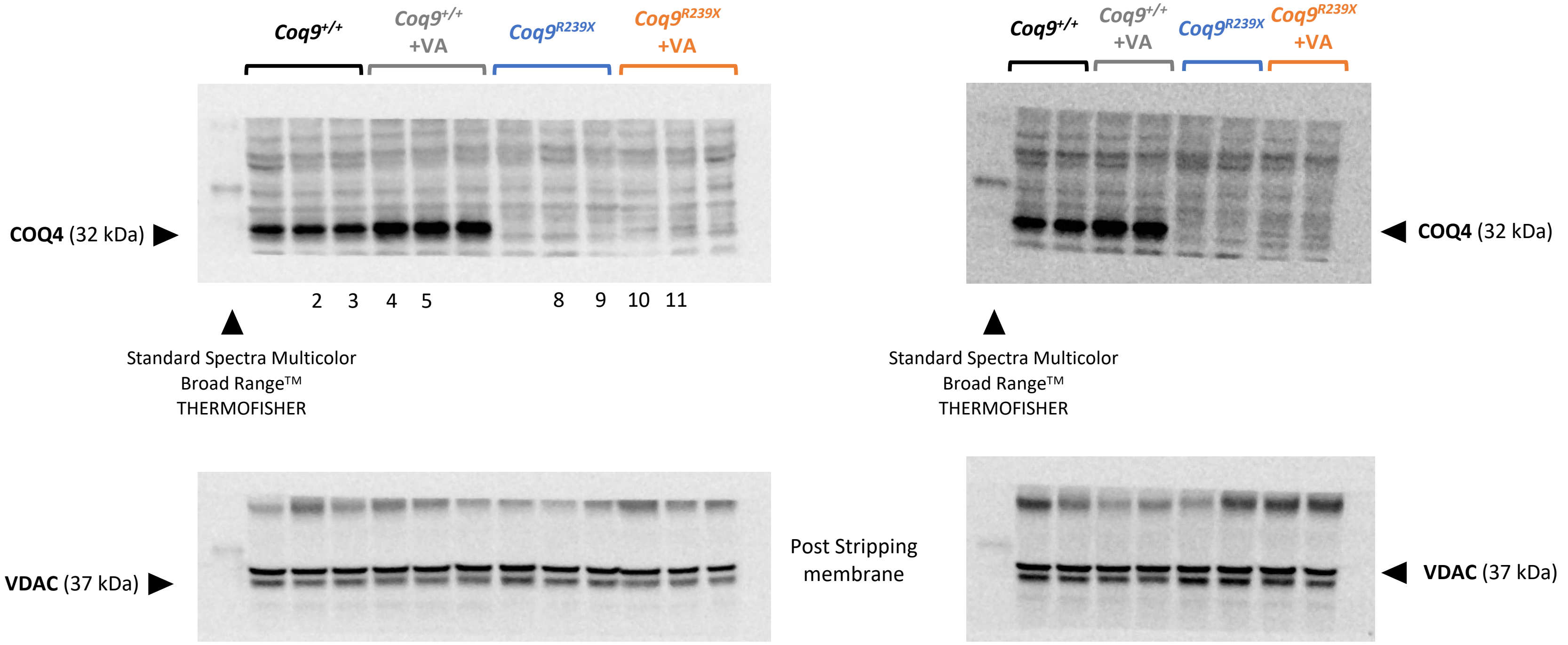

All membranes were cut before the incubation with the primary antibody.

Note: lines 2, 3, 4, 5, 8, 9, 10 and 11 are represented in Figure 3R in the main text.

Figure S5. COQ5 in liver of wild-type and mutant mice with and without treatment.

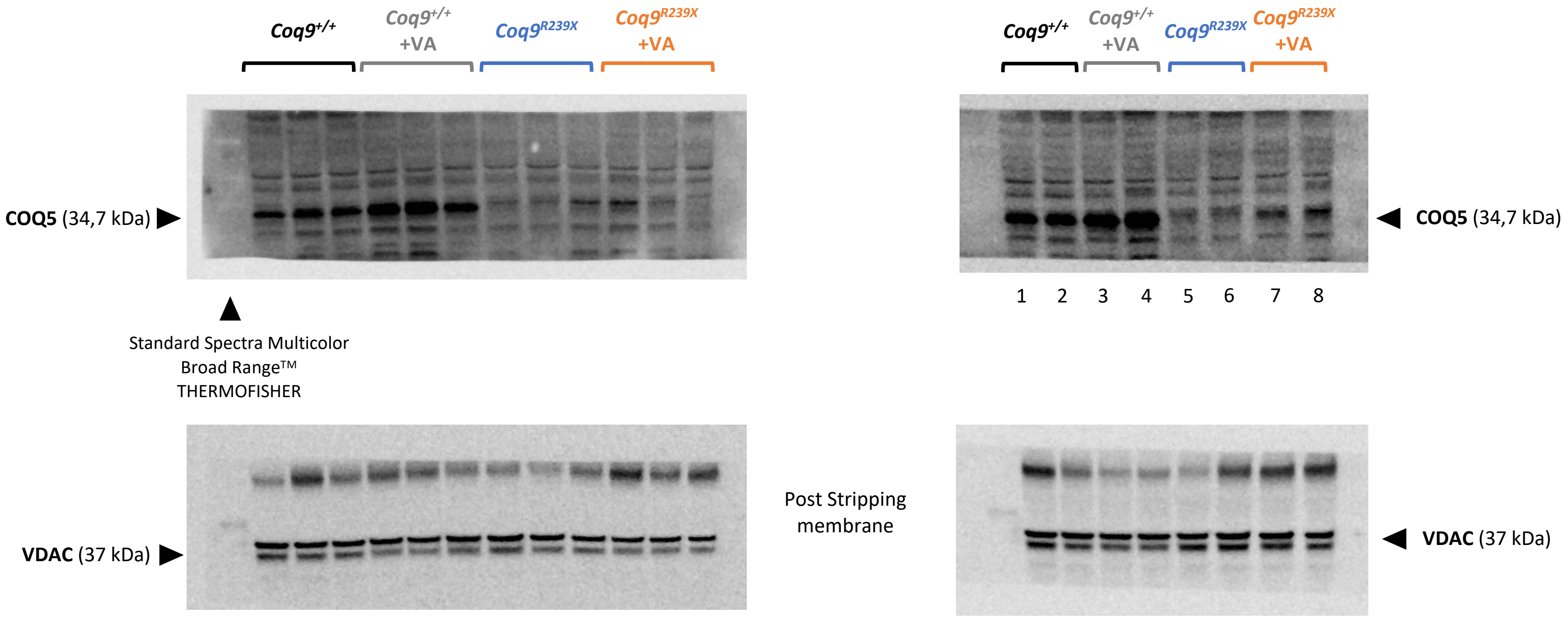

All membranes were cut before the incubation with the primary antibody.

Note: lines 1, 2, 3, 4, 5, 6, 7 and 8 are represented in Figure 3S in the main text.

Figure S5. COQ7 in liver of wild-type and mutant mice with and without treatment.

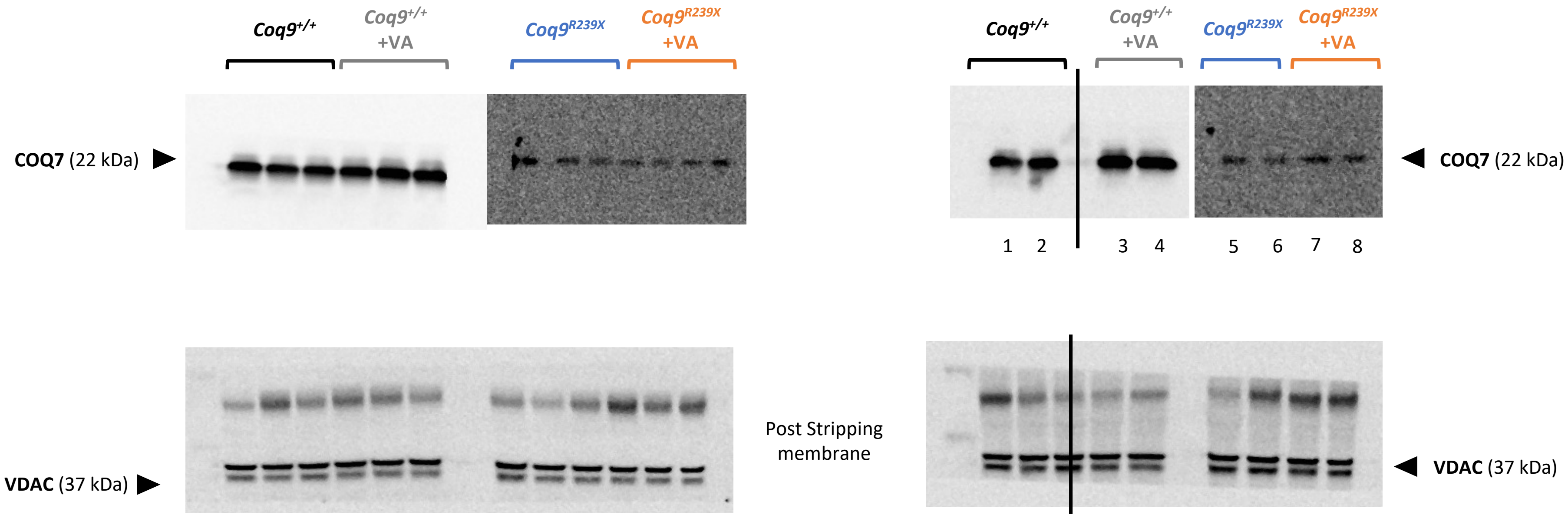

All membranes were cut before the incubation with the primary antibody.

Note: lines 1, 2, 3, 4, 5, 6, 7 and 8 are represented in Figure 3T in the main text.

Figure S9. PRODH in brain of wild-type and mutant mice with and without treatment.

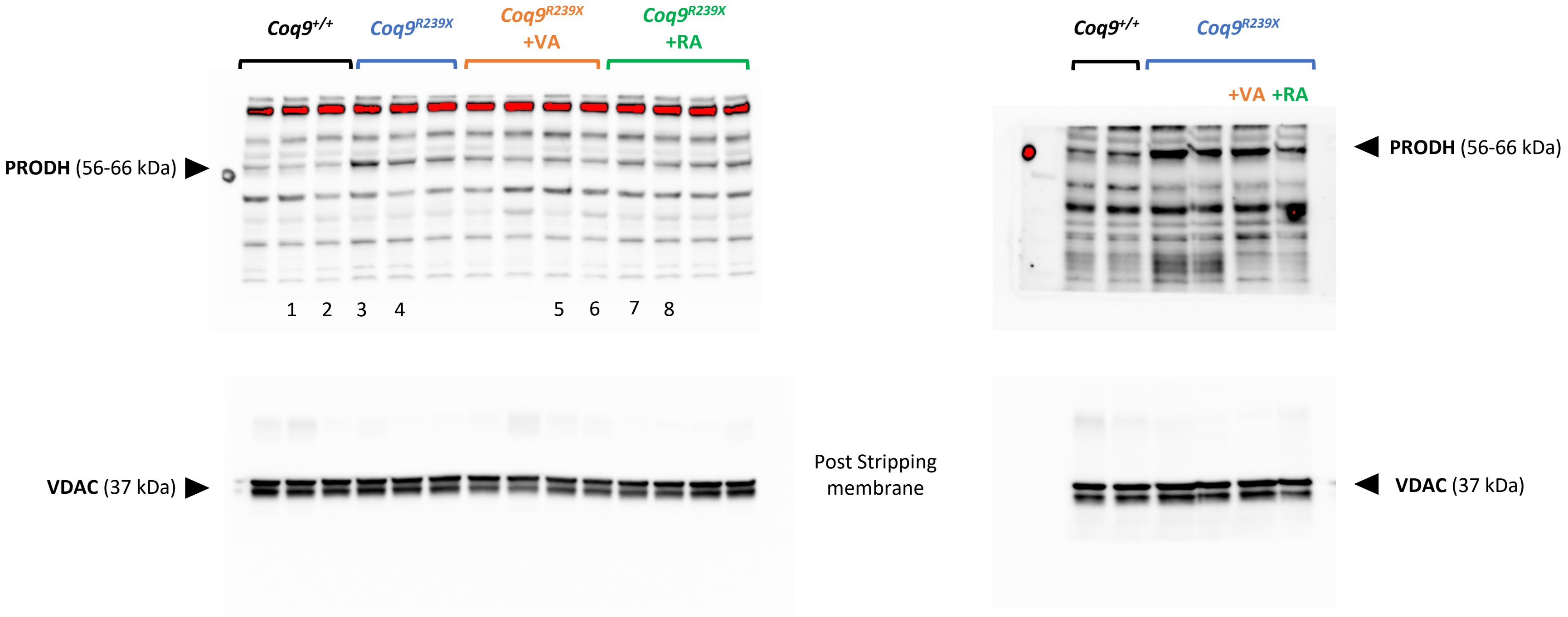

All membranes were cut before the incubation with the primary antibody.

Note: lines 1, 2, 3, 4, 5, 6, 7 and 8 are represented in Figure S8A in supplemental material.

Figure S9. PRODH in kidney of wild-type and mutant mice with and without treatment.

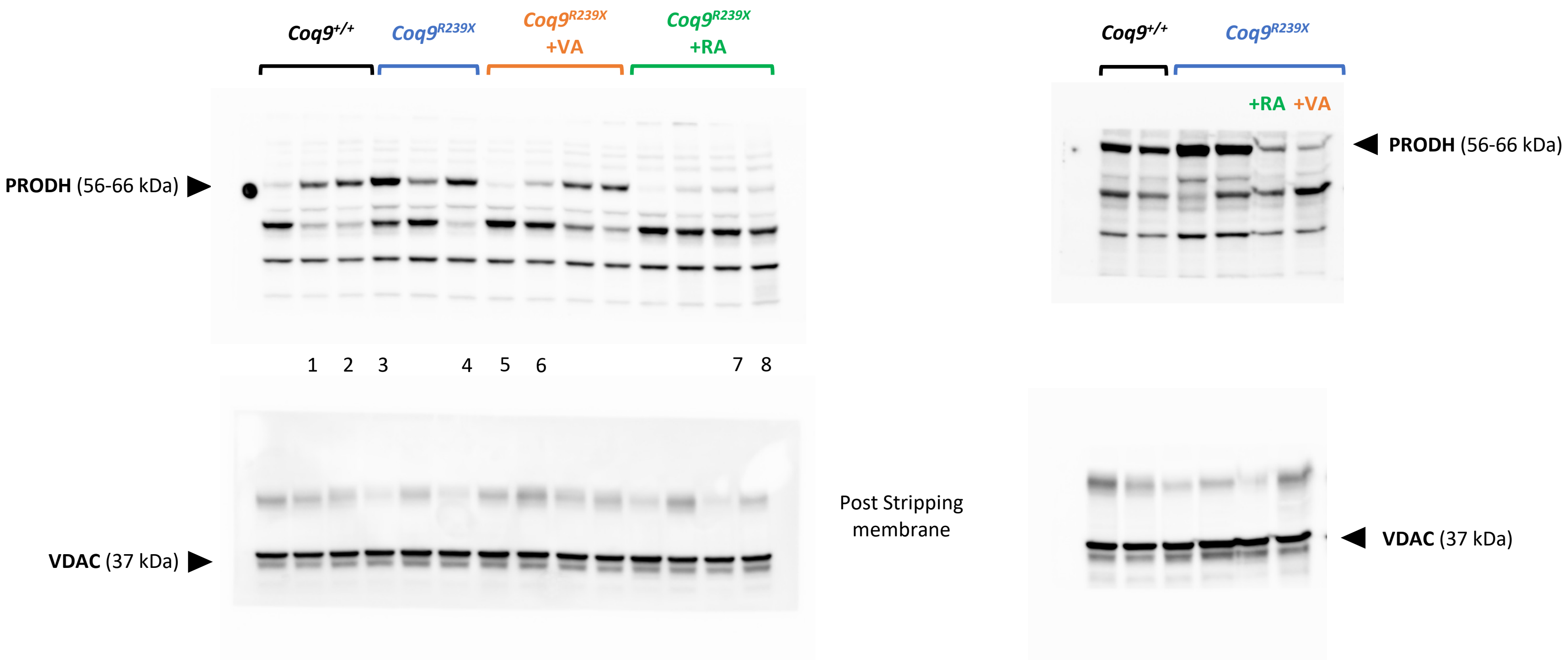

All membranes were cut before the incubation with the primary antibody.

Note: lines 1, 2, 3, 4, 5, 6, 7 and 8 are represented in Figure S8B in supplemental material.

Figure S9. DMGDH in kidney of wild-type and mutant mice with and without treatment.

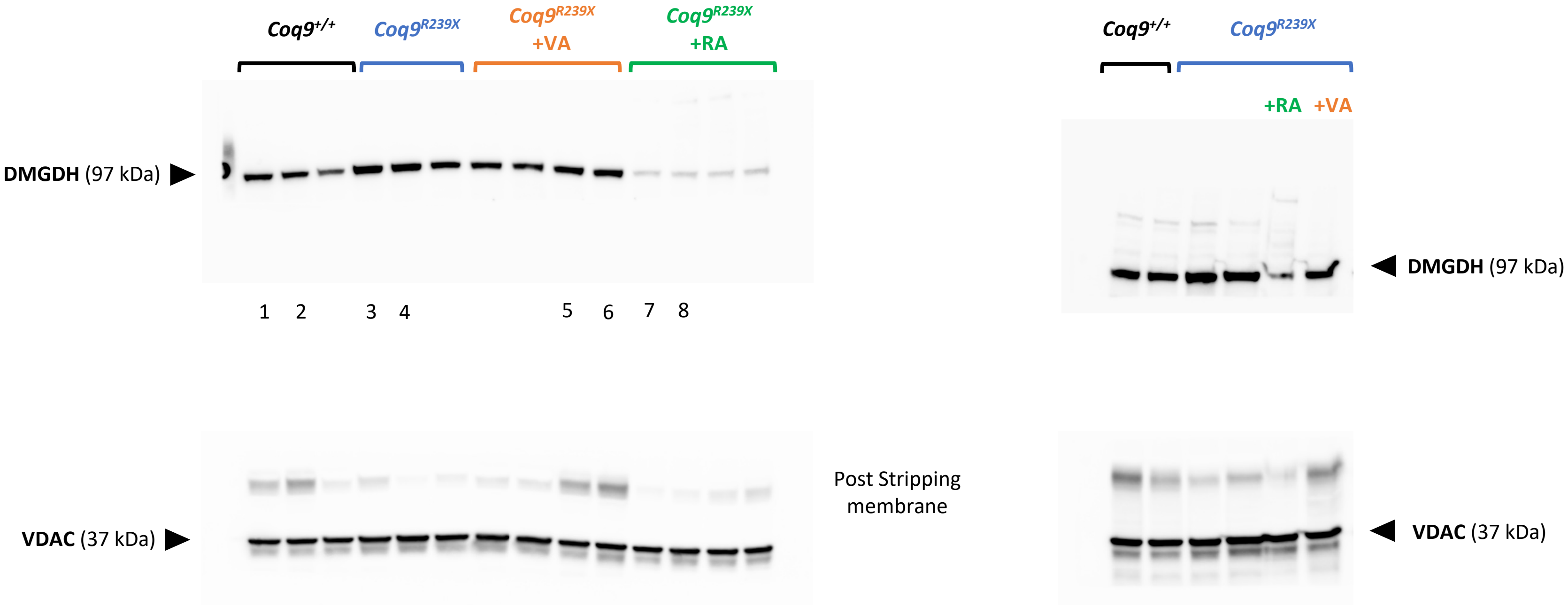

All membranes were cut before the incubation with the primary antibody.

Note: lines 1, 2, 3, 4, 5, 6, 7 and 8 are represented in Figure S8C in supplemental material.

Figure S9. OPA1 in kidney of wild-type and mutant mice with and without treatment.

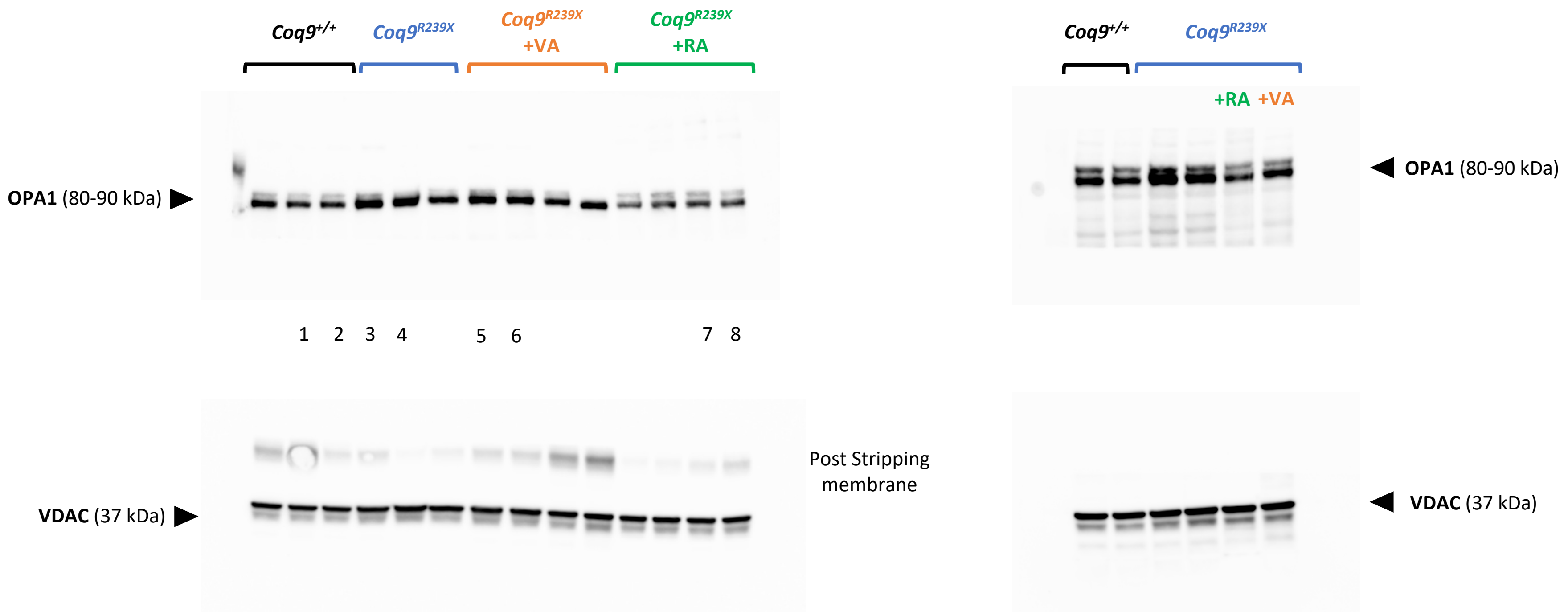

All membranes were cut before the incubation with the primary antibody.

Note: lines 1, 2, 3, 4, 5, 6, 7 and 8 are represented in Figure S8D in supplemental material.

Figure S9. OPA1 in kidney of wild-type and mutant mice with and without treatment.

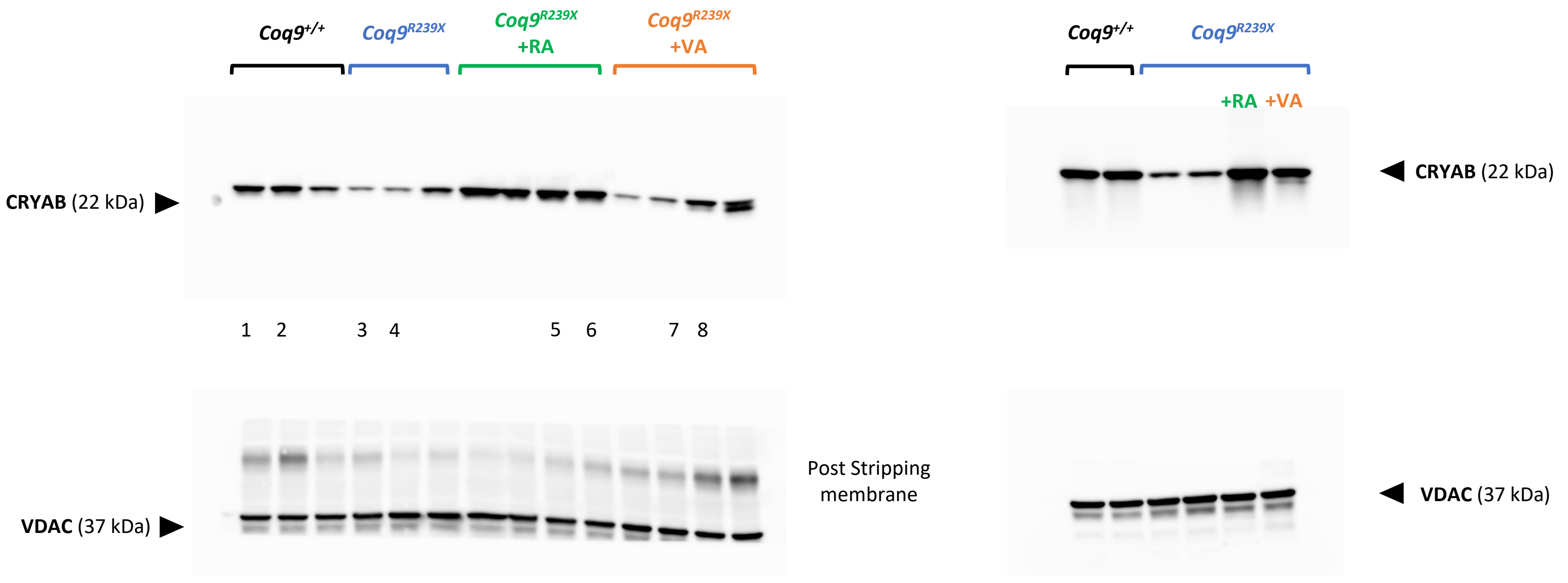

All membranes were cut before the incubation with the primary antibody.

Note: lines 1, 2, 3, 4, 5, 6, 7 and 8 are represented in Figure S8E in supplemental material.
