# Supplementary material for: The Q-junction and the inflammatory response are critical pathological and therapeutic factors in CoQ deficiency
Source: Redox Biol. 2022 Jul 15;55:102403. doi: 10.1016/j.redox.2022.102403 (PMC9301574; doi:10.1016/j.redox.2022.102403)
Supplement: Multimedia component 3 [file mmc3.pdf]

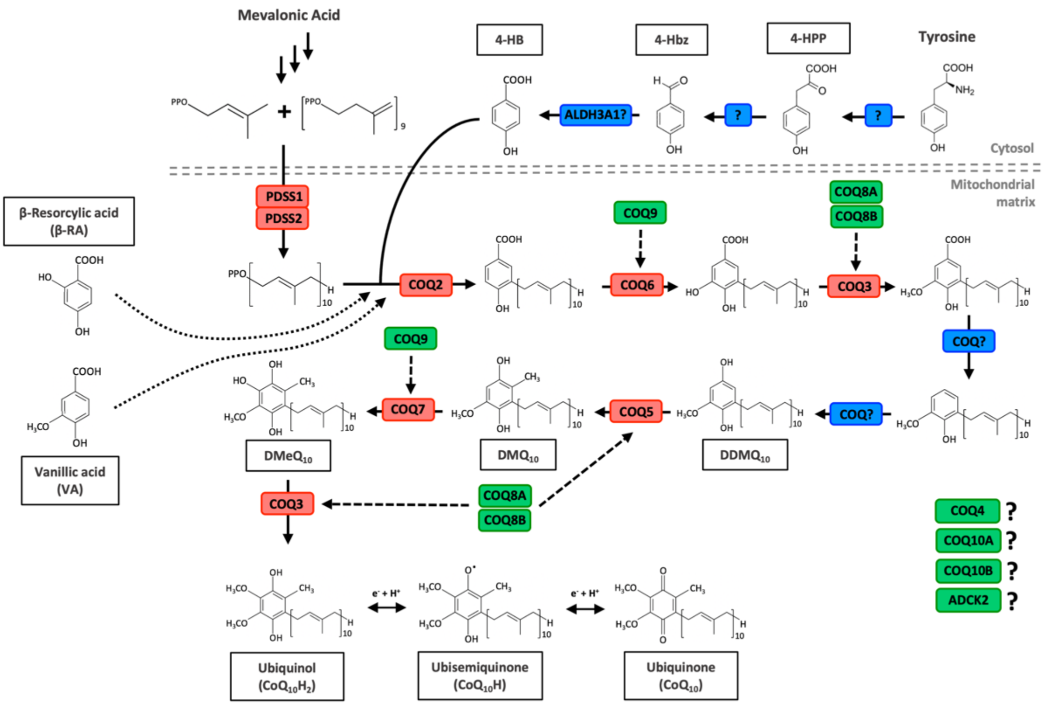

Figure S1. Coenzyme Q_{10} biosynthetic pathway.

Schematic model of human CoQ_{10} biosynthetic pathway. In red color are represented proteins with enzymatic activity. Green color indicates proteins with regulatory function. Blue color shows currently unidentified enzymes.

4-HB = 4-Hydroxybenzoic Acid; 4-Hbz = 4-Hydroxybenzaldehyde; 4-HPP = 4-Hydroxyphenylpiruvate; DDMQ = Demethoxy-Demethyl-Coenzyme Q; DMQ = Demethoxy-Coenzyme Q; DMeQ = Demethyl-Coenzyme Q.

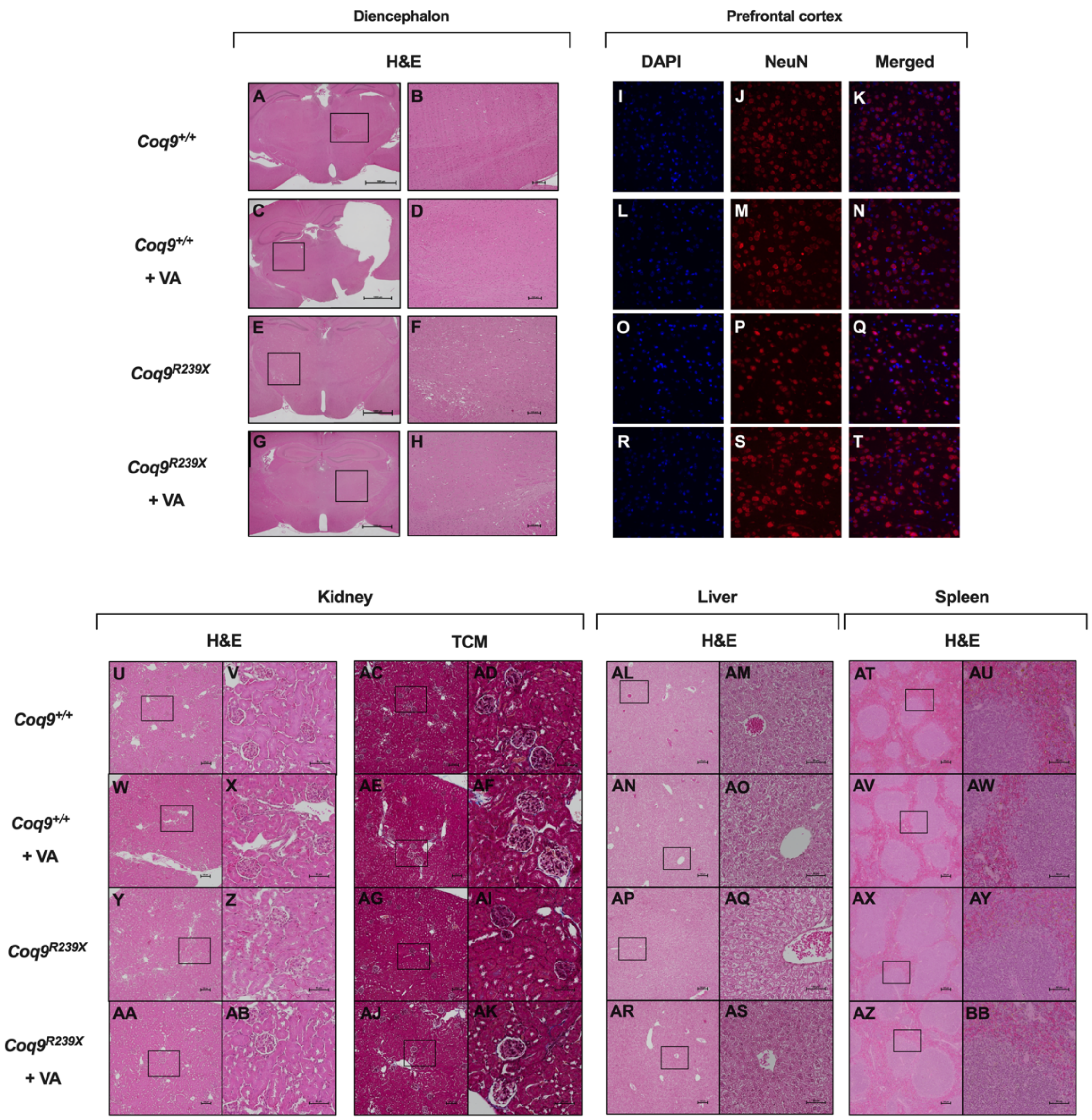

Figure S2. Morphological and histological features of the brain, liver, kidney and spleen of *Coq9^{R239X}* mice under 1% of VA treatment.

(**A-H**) H&E stain in the diencephalon of *Coq9$^{+/+}$* mice (**A** and **B**), *Coq9$^{+/+}$* mice under 1% VA supplementation (**C** and **D**), *Coq9^{R239X}* mice (**E** and **F**) and *Coq9^{R239X}* mice under 1% VA supplementation (**G** and **H**) at 3 months of age.

(**I-T**) NeuN stain in the prefrontal cortex of *Coq9$^{+/+}$* mice (**I, J** and **K**), *Coq9$^{+/+}$* mice under 1% VA supplementation (**L, M** and **N**), *Coq9^{R239X}* mice (**O, P** and **Q**) and *Coq9^{R239X}* mice under 1% VA supplementation (**R, S** and **T**) at 3 months of age.

(**U-AB; AL-BB**) H&E stain in the kidney, liver and spleen of *Coq9*$^{+/+}$ mice (**U, V; AL, AM; AT, AU**), *Coq9*$^{+/+}$ mice under 1% VA supplementation (**W, X; AN, AO; AV, AW**), *Coq9*^{R239X} mice (**Y, Z; AP, AQ; AX, AY**) and *Coq9*^{R239X} mice under 1% VA supplementation (**AA, AB; AR, AS; AZ, BB**) at 3 months of age.

(**AC-AK**) Masson's trichrome stain in the kidney of *Coq9*$^{+/+}$ mice (**AC** and **AD**), *Coq9*$^{+/+}$ mice under 1% VA supplementation (**AE** and **AF**), *Coq9*^{R239X} mice (**AG** and **AI**) and *Coq9*^{R239X} mice under 1% VA supplementation (**AJ** and **AK**) at 3 months of age.

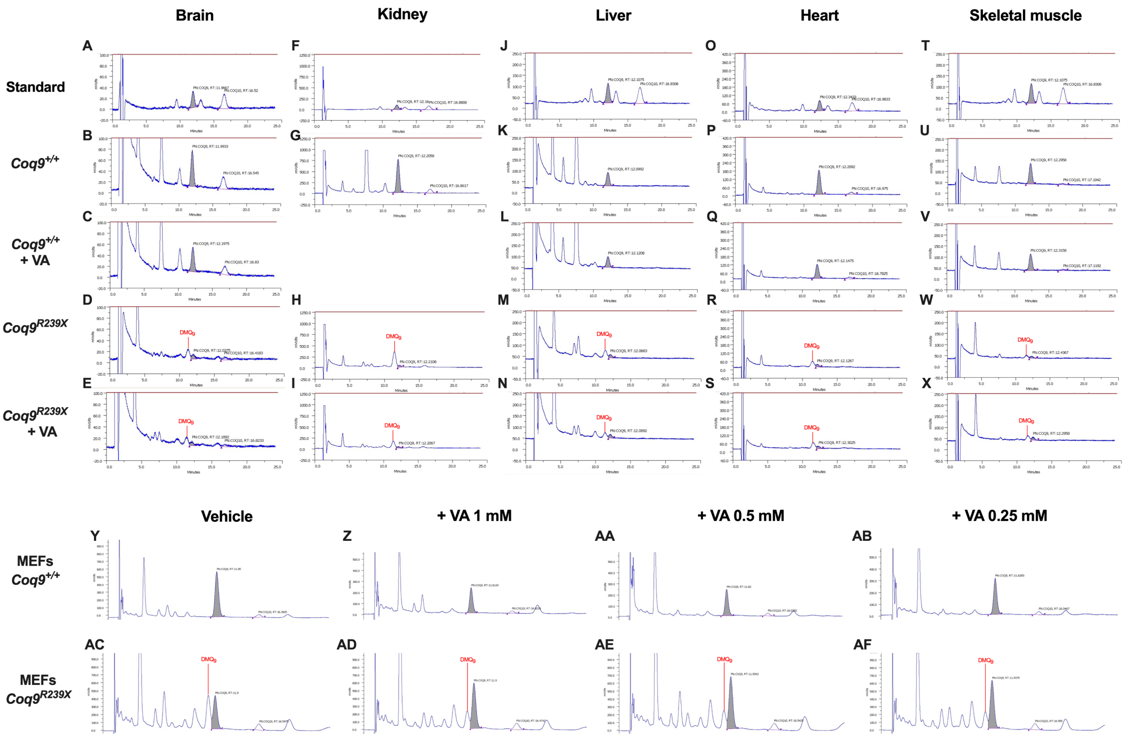

Figure S3. Representative chromatographs of CoQ_9 and DMQ_9 in MEFs and mice tissues after VA treatment.

(**A, F, J, O** and **T**) Chromatographs from CoQ standard.

(**B-E**) Chromatographs from brain samples of *Coq9*$^{+/+}$ mice (**B**), *Coq9*$^{+/+}$ mice under 1% VA supplementation (**C**), *Coq9*^{R239X} mice (**D**) and *Coq9*^{R239X} mice under 1% VA supplementation (**E**) at 3 months of age.

(**G, H** and **I**) Chromatographs from kidney samples of *Coq9*$^{+/+}$ mice (**G**), *Coq9*^{R239X} mice (**H**) and *Coq9*^{R239X} mice under 1% VA supplementation (**I**) at 3 months of age.

(**K-N**) Chromatographs from liver samples of *Coq9*$^{+/+}$ mice (**K**), *Coq9*$^{+/+}$ mice under 1% VA supplementation (**L**), *Coq9*^{R239X} mice (**M**) and *Coq9*^{R239X} mice under 1% VA supplementation (**N**) at 3 months of age.

(**P-S**) Chromatographs from heart samples of *Coq9*$^{+/+}$ mice (**P**), *Coq9*$^{+/+}$ mice under 1% VA supplementation (**Q**), *Coq9*^{R239X} mice (**R**) and *Coq9*^{R239X} mice under 1% VA supplementation (**S**) at 3 months of age.

(**U-X**) Chromatographs from skeletal muscle samples of *Coq9*$^{+/+}$ mice (**U**), *Coq9*$^{+/+}$ mice under 1% VA supplementation (**V**), *Coq9*^{R239X} mice (**W**) and *Coq9*^{R239X} mice under 1% VA supplementation (**X**) at 3 months of age.

(**Y-AB**) Chromatographs from *Coq9*$^{+/+}$ MEFs treated 7 days with vehicle (**Y**), VA 1mM (**Z**), VA 0.5 mM (**AA**), VA 0.25 mM (**AB**).

(**AC-AF**) Chromatographs from *Coq9*^{R239X} MEFs treated 7 days with vehicle (**AC**), VA 1mM (**AD**), VA 0.5 mM (**AE**), VA 0.25 mM (**AF**).

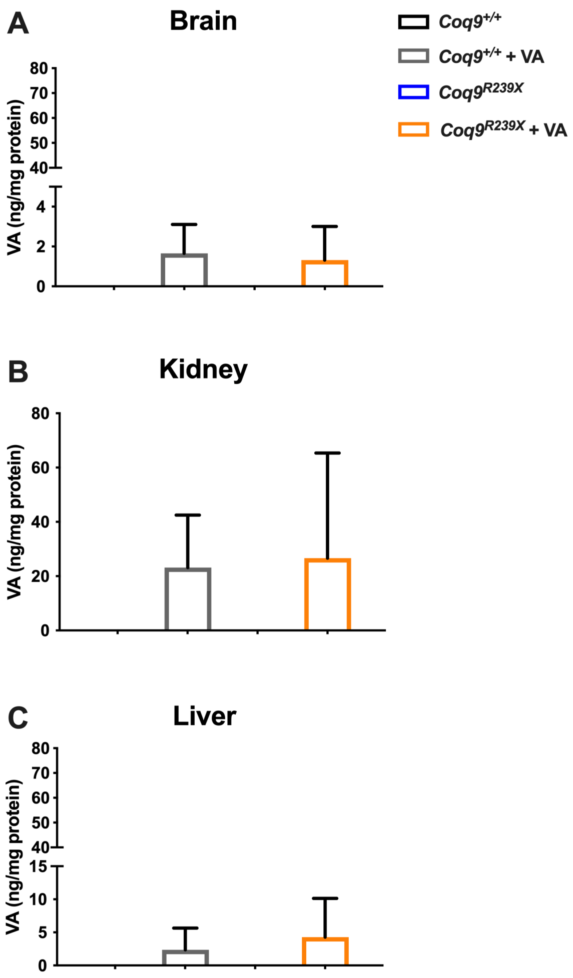

Figure S4. Levels of VA in mouse tissues after de oral supplementation of VA.

(**A, B** and **C**) Levels of VA in brain (**A**), kidney (**B**) and liver (**C**) of *Coq9*$^{+/+}$ mice, *Coq9*$^{+/+}$ mice under 1% VA supplementation, *Coq9*^{R239X} mice and *Coq9*^{R239X} mice under 1% VA supplementation at 3 months of age.

Data are expressed as mean ± SD. *P < 0.05, **P < 0.01, ***P < 0.001, differences *versus* *Coq9*$^{+/+}$; $^{\#}$P < 0.05, $^{\#\#}$P < 0.01, $^{\#\#\#}$P < 0.001, differences *versus* *Coq9*$^{+/+}$ after VA treatment; +P < 0.05, ++P < 0.01, +++P < 0.001, *versus* *Coq9*^{R239X}; (one-way ANOVA with a Tukey's *post hoc* test; n = 5 for each group).

Figure S5. COQ2 in MEFs of wild-type and mutant mice with and without treatment.

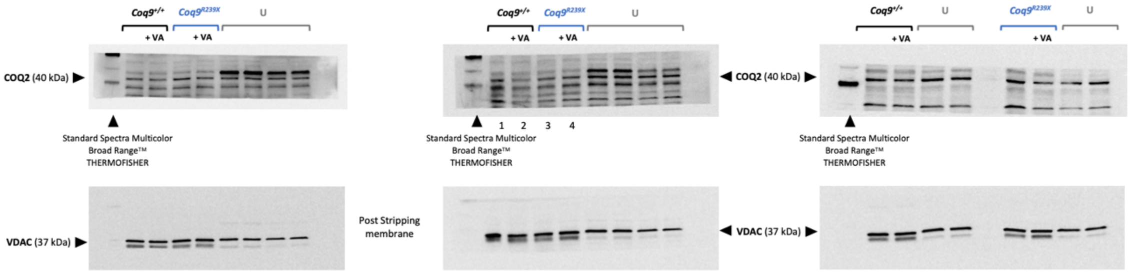

All membranes were cut before the incubation with the primary antibody.

Note: lines 1, 2, 3 and 4 are represented in Figure 3A in the main text.

U = Unrelated to this study

Figure S5. COQ4 in MEFs of wild-type and mutant mice with and without treatment.

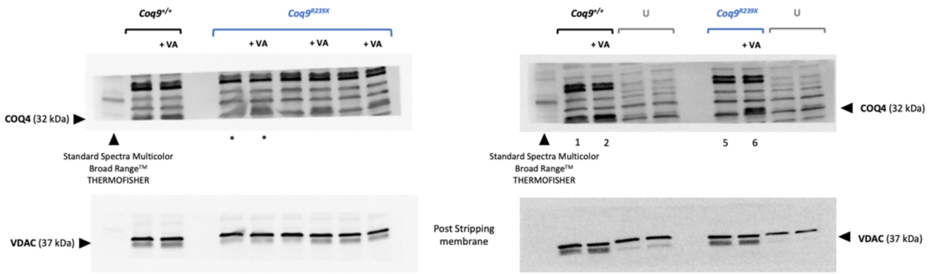

All membranes were cut before the incubation with the primary antibody.

Note: lines 1, 2, 5 and 6 are represented in Figure 3B in the main text.

U = Unrelated to this study

* Repeted samples 5 and 6.

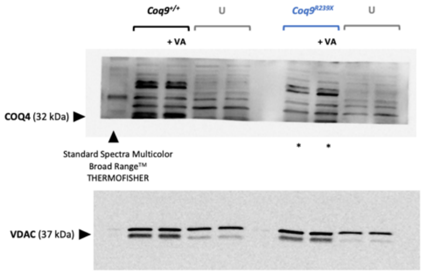

Figure S5. COQ5 in MEFs of wild-type and mutant mice with and without treatment.

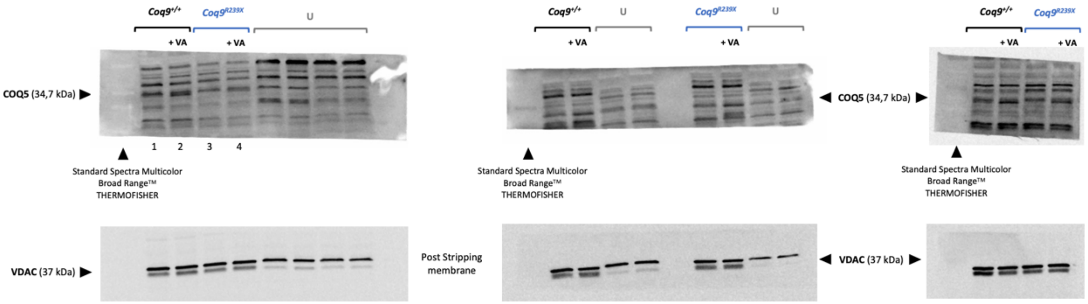

All membranes were cut before the incubation with the primary antibody.

Note: lines 1, 2, 3 and 4 are represented in Figure 3C in the main text.

U = Unrelated to this study

Figure S5. COQ7 in MEFs of wild-type and mutant mice with and without treatment.

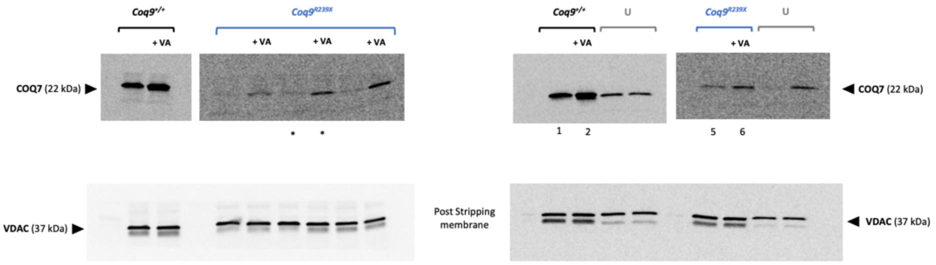

All membranes were cut before the incubation with the primary antibody.

Note: lines 1, 2, 5 and 6 are represented in Figure 3D in the main text.

U = Unrelated to this study

* Repeted samples 5 and 6.

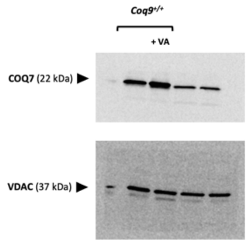

Figure S5. COQ2 in brain of wild-type and mutant mice with and without treatment.

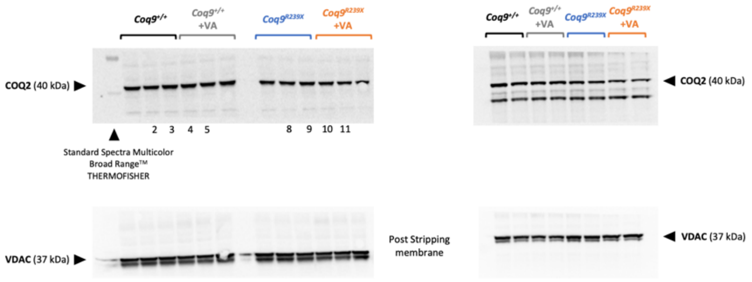

All membranes were cut before the incubation with the primary antibody.

Note: lines 2, 3, 4, 5, 8, 9, 10 and 11 are represented in Figure 3I in the main text.

Figure S5. COQ4 in brain of wild-type and mutant mice with and without treatment.

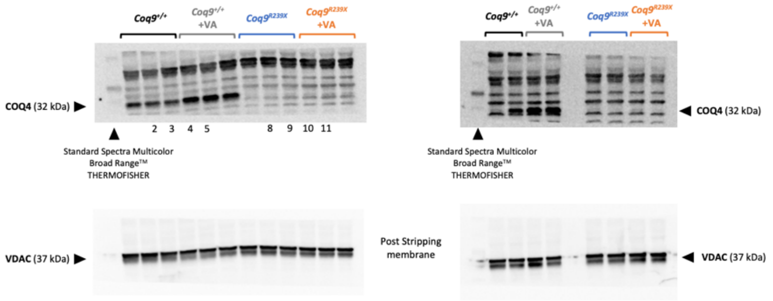

All membranes were cut before the incubation with the primary antibody.

Note: lines 2, 3, 4, 5, 8, 9, 10 and 11 are represented in Figure 3J in the main text.

Figure S5. COQ5 in brain of wild-type and mutant mice with and without treatment.

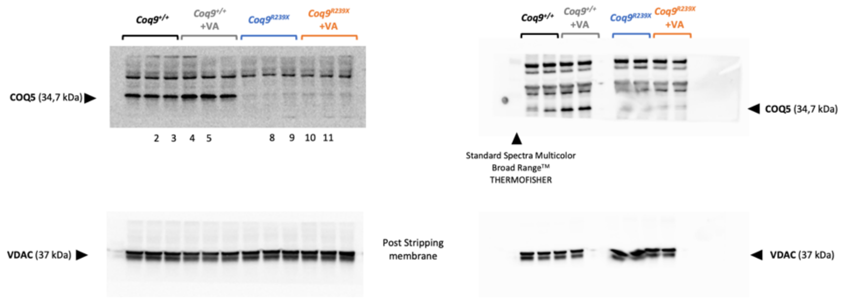

All membranes were cut before the incubation with the primary antibody.

Note: lines 2, 3, 4, 5, 8, 9, 10 and 11 are represented in Figure 3K in the main text.

Figure S5. COQ7 in brain of wild-type and mutant mice with and without treatment.

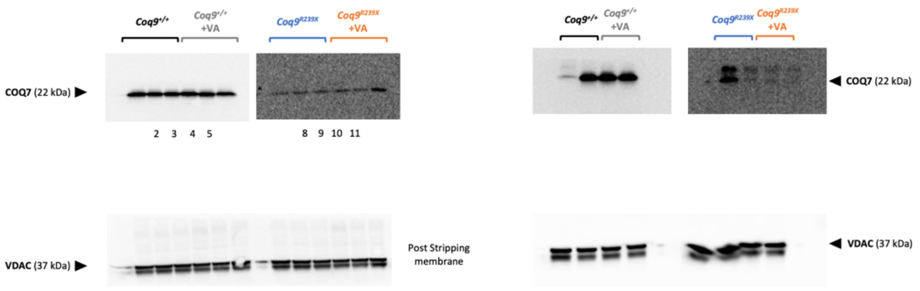

All membranes were cut before the incubation with the primary antibody.

Note: lines 2, 3, 4, 5, 8, 9, 10 and 11 are represented in Figure 3L in the main text.

Figure S5. COQ2 in kidney of wild-type and mutant mice with and without treatment.

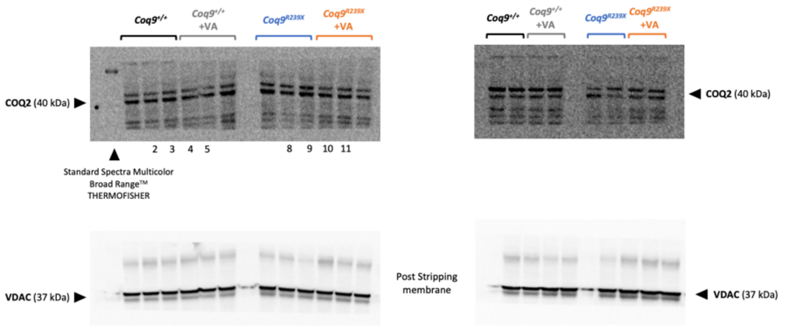

All membranes were cut before the incubation with the primary antibody.

Note: lines 2, 3, 4, 5, 8, 9, 10 and 11 are represented in Figure 3M in the main text.

Figure S5. COQ4 in kidney of wild-type and mutant mice with and without treatment.

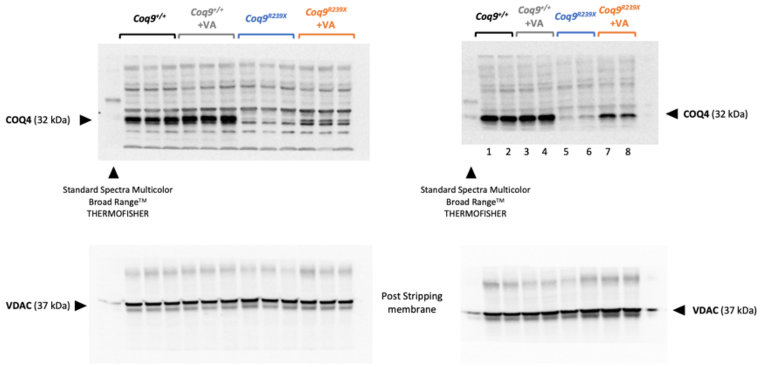

All membranes were cut before the incubation with the primary antibody.

Note: lines 1, 2, 3, 4, 5, 6, 7 and 8 are represented in Figure 3N in the main text.

Figure S5. COQ5 in kidney of wild-type and mutant mice with and without treatment.

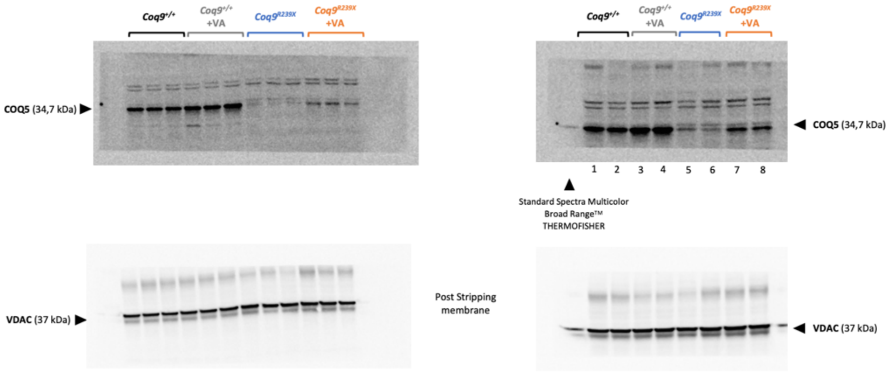

All membranes were cut before the incubation with the primary antibody.

Note: lines 1, 2, 3, 4, 5, 6, 7 and 8 are represented in Figure 3O in the main text.

Figure S5. COQ7 in kidney of wild-type and mutant mice with and without treatment.

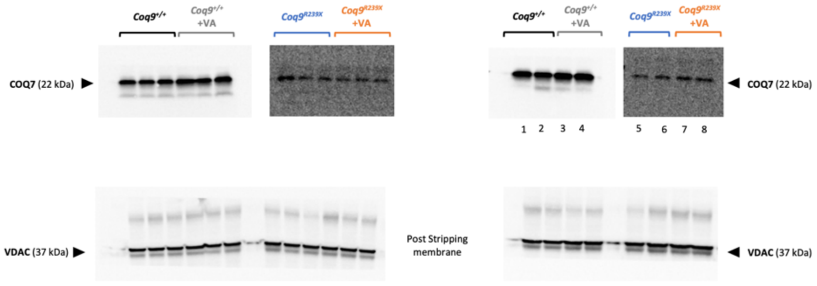

All membranes were cut before the incubation with the primary antibody.

Note: lines 1, 2, 3, 4, 5, 6, 7 and 8 are represented in Figure 3P in the main text.

Figure S5. COQ2 in liver of wild-type and mutant mice with and without treatment.

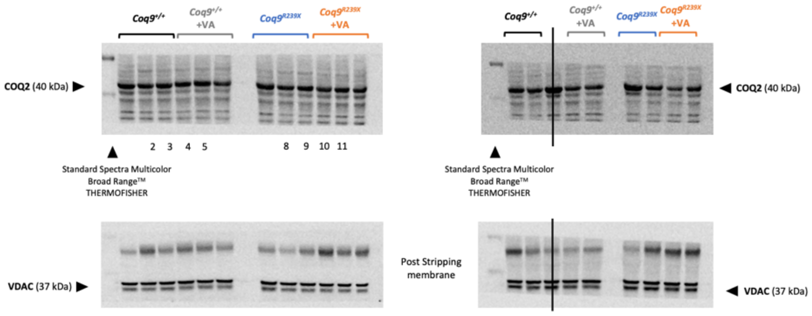

All membranes were cut before the incubation with the primary antibody.

Note: lines 2, 3, 4, 5, 8, 9, 10 and 11 are represented in Figure 3Q in the main text.

Figure S5. COQ4 in liver of wild-type and mutant mice with and without treatment.

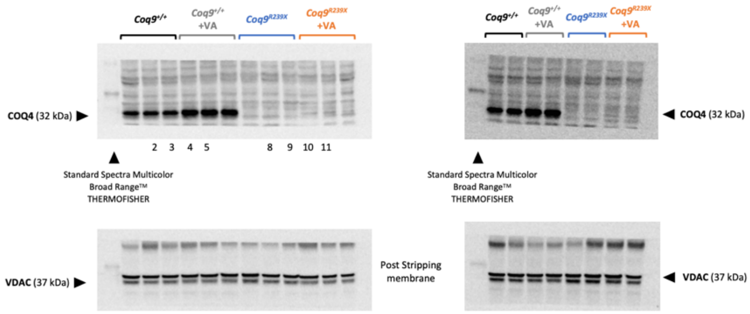

All membranes were cut before the incubation with the primary antibody.

Note: lines 2, 3, 4, 5, 8, 9, 10 and 11 are represented in Figure 3R in the main text.

Figure S5. COQ5 in liver of wild-type and mutant mice with and without treatment.

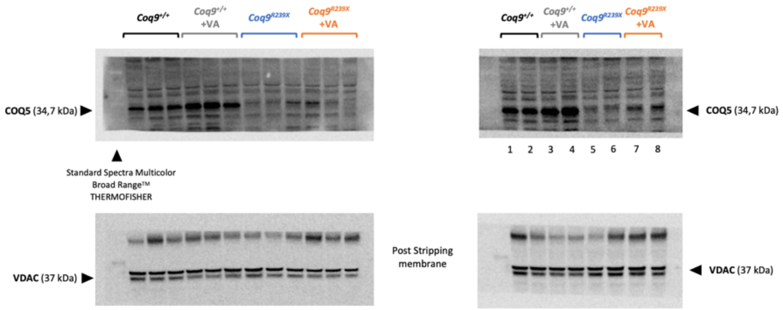

All membranes were cut before the incubation with the primary antibody.

Note: lines 1, 2, 3, 4, 5, 6, 7 and 8 are represented in Figure 3S in the main text.

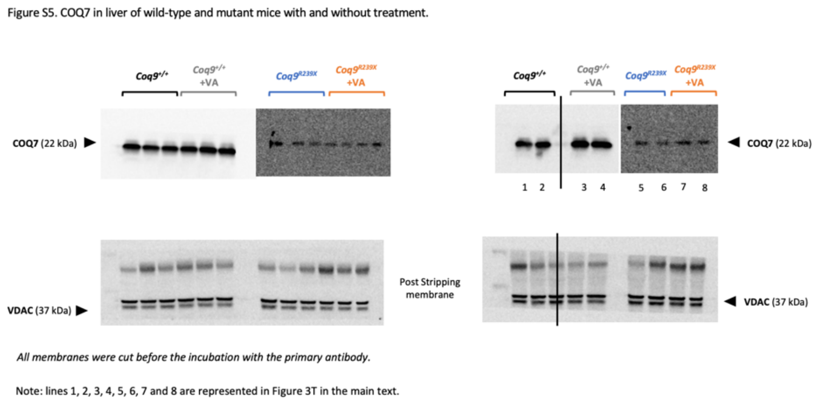

Figure S5. Original membranes of the Western blots of figure 3.

Original membranes for the detection of the CoQ biosynthetic proteins COQ2**,** COQ4, COQ5 and COQ7 in MEFs from *Coq9*$^{+/+}$ and *Coq9*^{R239X} mice after supplementation with VA at 1mM; and in the brain, kidney and liver of *Coq9*$^{+/+}$ mice, *Coq9*$^{+/+}$ mice after 1% VA treatment, *Coq9*^{R239X} mice and *Coq9*^{R239X} mice after 1% VA treatment.

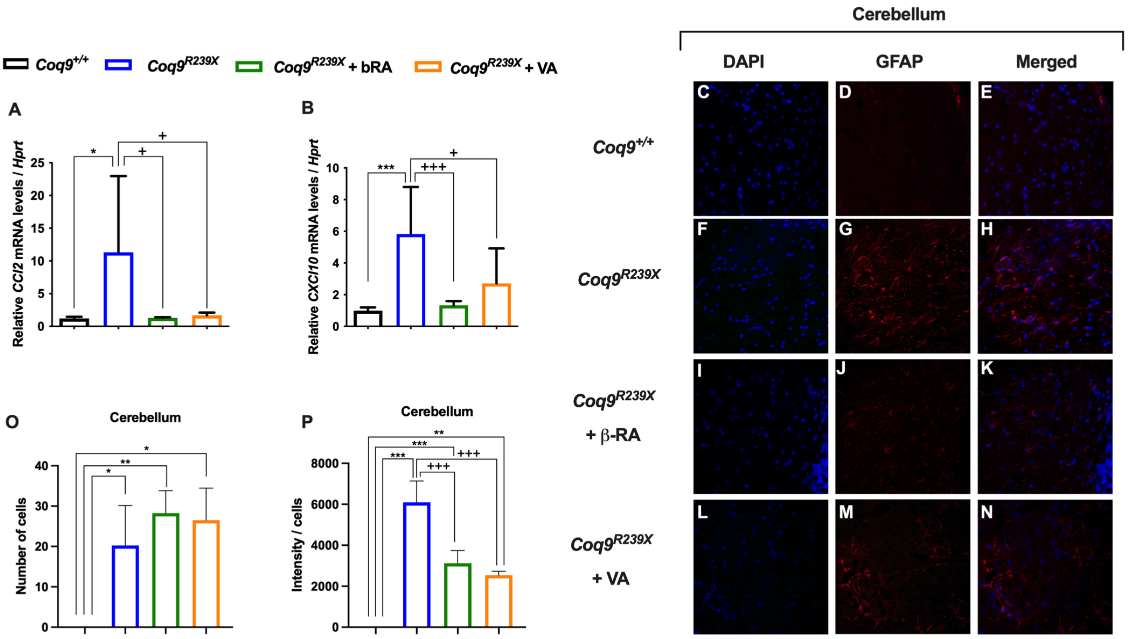

Figure S6. Validation of CCl2 and CXCl10 in brainstem and GFAP stain in the cerebellum of the mutant mice after β-RA and VA treatment.

(**A** and **B**) Relative mRNA levels of *CCl2 (***A***)* and *CXCL10 (***B***)* in the brain of $Coq9^{+/+}$, $Coq9^{R239X}$ mice, $Coq9^{R239X}$ mice under 1% β-RA and $Coq9^{R239X}$ mice under 1% VA supplementation at 3 months of age.

(**C-N**) GFAP stain in the cerebellum of $Coq9^{+/+}$ mice (**C, D** and **E**), $Coq9^{R239X}$ mice (**F, G** and **H**), $Coq9^{R239X}$ mice under 1% β-RA supplementation (**I, J** and **K**) and $Coq9^{R239X}$ mice under 1% VA supplementation (**L, M** and **N**) at 3 months of age.

(**O** and **P**) Quantification of GFAP expression by number of cells (**O**) and intensity/cells (**P**) in the cerebellum of $Coq9^{+/+}$ mice, $Coq9^{R239X}$ mice, $Coq9^{R239X}$ mice under 1% β-RA supplementation and $Coq9^{R239X}$ mice under 1% VA supplementation at 3 months of age. Data are expressed as mean ± SD. *P < 0.05, **P < 0.01, ***P < 0.001, differences *versus* $Coq9^{+/+}$; +P < 0.05, ++P < 0.01, +++P < 0.001, *versus* $Coq9^{R239X}$; (one-way ANOVA with a Tukey's *post hoc* test; n = 5 for each group).

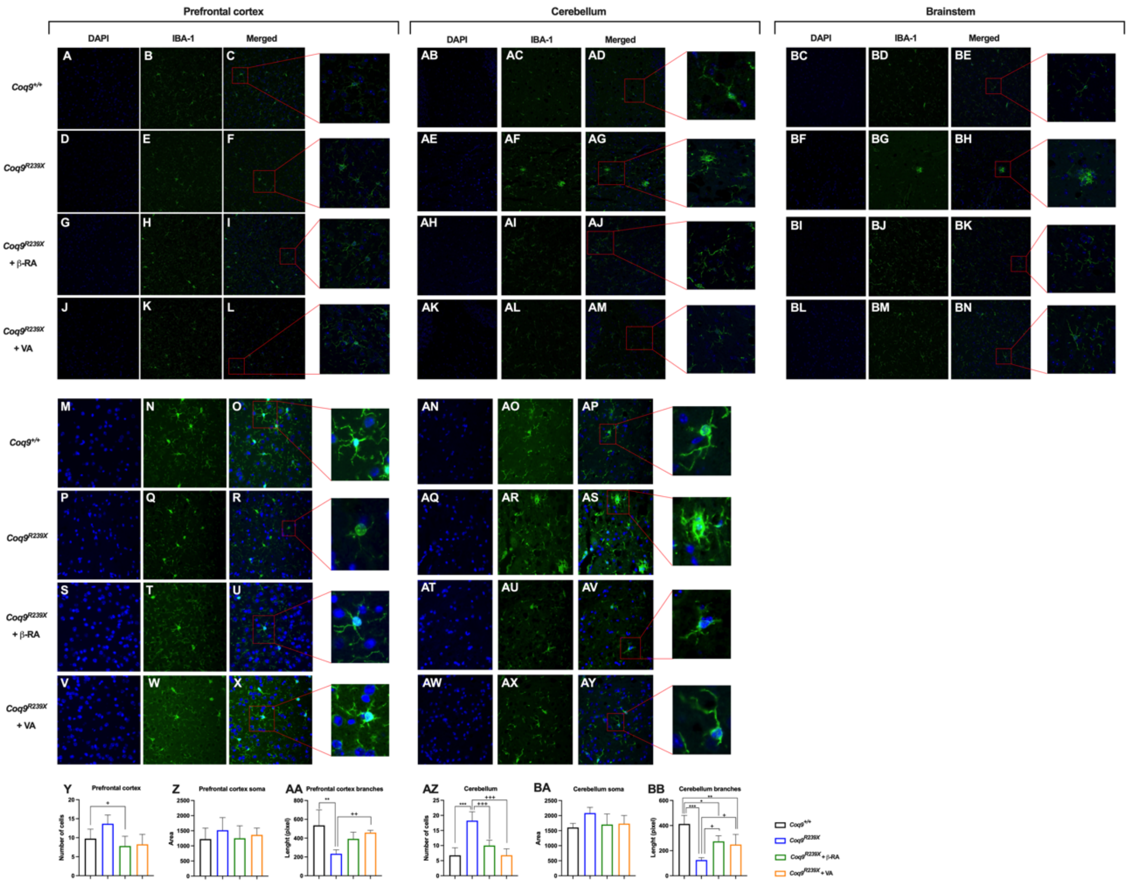

Figure S7. IBA-1 stain in the brain of the mutant mice after β-RA and VA treatment.

(**A**-**X**) IBA-1 stain in the prefrontal cortex of *Coq9*$^{+/+}$ mice, *Coq9*^{R239X} mice, *Coq9*^{R239X} mice under 1% β-RA supplementation and *Coq9*^{R239X} mice under 1% VA supplementation at 3 months of age under confocal microscope (**A-L**) and fluorescence microscope (**M-X**).

(**Y, Z** and **AA**) Quantification of IBA-1 expression by number of cells (**Y**) and phenotype analysis of soma area (**Z**) and branches length (**AA**) in the prefrontal cortex of *Coq9*$^{+/+}$ mice, *Coq9*^{R239X} mice, *Coq9*^{R239X} mice under 1% β-RA supplementation and *Coq9*^{R239X} mice under 1% VA supplementation at 3 months of age.

(**AB**-**AY**) IBA-1 stain in the cerebellum of *Coq9*$^{+/+}$ mice, *Coq9*^{R239X} mice, *Coq9*^{R239X} mice under 1% β-RA supplementation and *Coq9*^{R239X} mice under 1% VA supplementation at 3 months of age under confocal microscope (**AB-AM**) and fluorescence microscope (**AN-AY**).

(**AZ, BA** and **BB**) Quantification of IBA-1 expression by number of cells (**AZ**) and phenotype analysis of soma area (**BA**) and branches length (**BB**) in the cerebellum of *Coq9*$^{+/+}$ mice, *Coq9*^{R239X} mice, *Coq9*^{R239X} mice under 1% β-RA supplementation and *Coq9*^{R239X} mice under 1% VA supplementation at 3 months of age.

(**BC-BN**) IBA-1 stain in the brainstem of *Coq9*$^{+/+}$ mice, *Coq9*^{R239X} mice, *Coq9*^{R239X} mice under 1% β-RA supplementation and *Coq9*^{R239X} mice under 1% VA supplementation at 3 months of age under confocal microscope.

Data are expressed as mean ± SD. *P < 0.05, **P < 0.01, ***P < 0.001, differences *versus* *Coq9*$^{+/+}$; +P < 0.05, ++P < 0.01, +++P < 0.001, *versus* *Coq9*^{R239X}; (one-way ANOVA with a Tukey's *post hoc* test; n = 5 for each group).

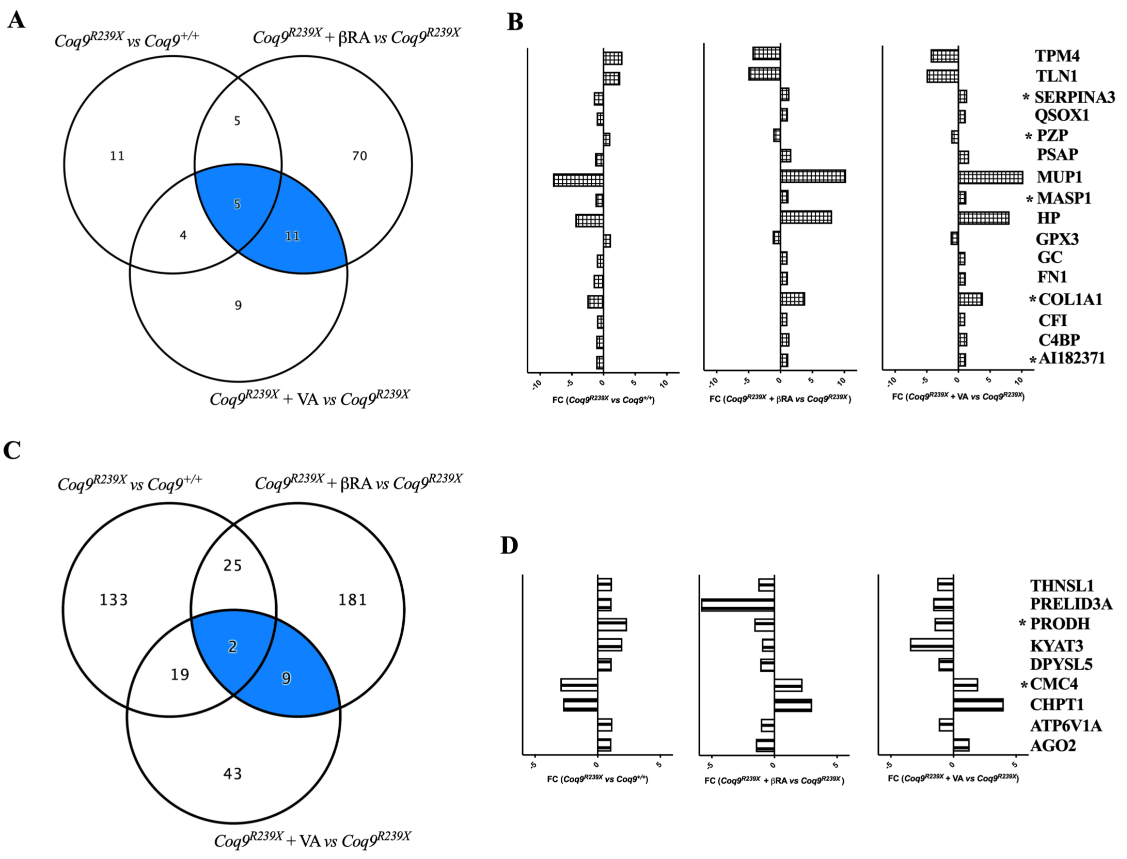

Figure S8. Mitochondrial proteome modulation after 4-HB analogs treatment in the plasma and brain of *Coq9^{R239X}* mice.

(**A**) Global differences in the protein levels between experimental groups in plasma. In blue are marked the 5 proteins altered by the mutation and by β-RA and VA treatments and the 11 proteins modified by β-RA and VA treatments.

(**B**) Fold change of the proteins modified by the mutation and normalized by β-RA and VA treatments in the plasma. $^{*}p < 0.05$. *Coq9$^{+/+}$*, n = 7; *Coq9^{R239X}*, n = 7; *Coq9^{R239X}* after β-RA treatment, n = 7; *Coq9^{R239X}* after VA treatment, n = 7.

(**C**) Global differences in the protein levels between experimental groups in brain. In blue are marked the 2 proteins altered by the mutation and by β-RA and VA treatments and the 9 proteins modified by β-RA and VA treatments.

(**D**) Fold change of the proteins modified by the mutation and normalized by β-RA and VA treatments in the brain mitochondrial proteome. $^{*}p < 0.05$. Mitochondrial proteomics was performed in isolated mitochondria. *Coq9*$^{+/+}$, n = 5; *Coq9*^{R239X}, n = 5; *Coq9*^{R239X} after β-RA treatment, n = 6; *Coq9*^{R239X} after VA treatment, n = 5.

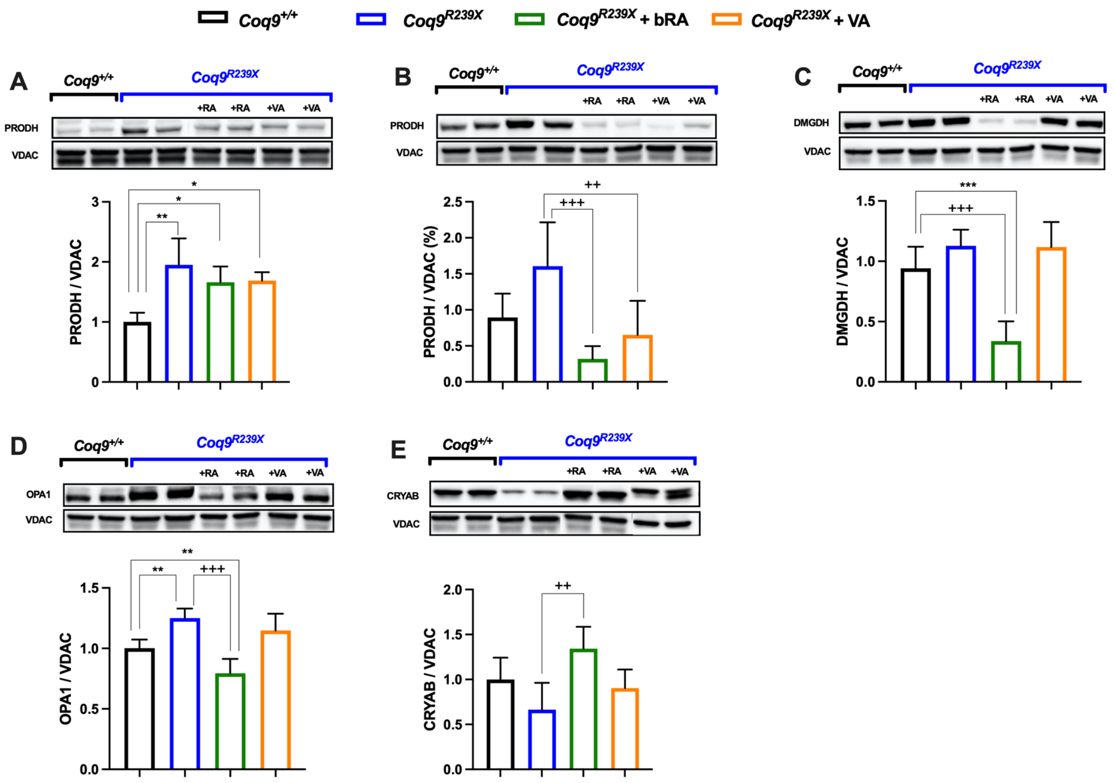

F

Figure S9. PRODH in brain of wild-type and mutant mice with and without treatment.

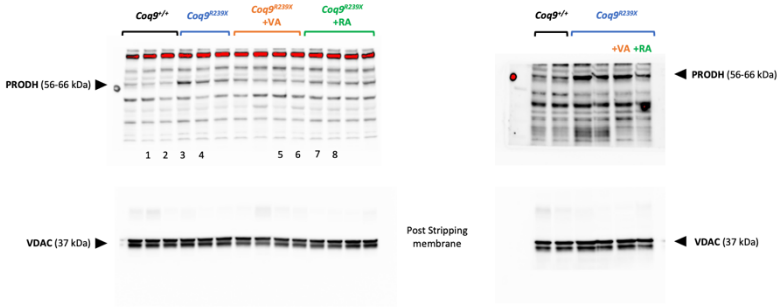

All membranes were cut before the incubation with the primary antibody.

Note: lines 1, 2, 3, 4, 5, 6, 7 and 8 are represented in Figure S8A in supplemental material.

Figure S9. PRODH in kidney of wild-type and mutant mice with and without treatment.

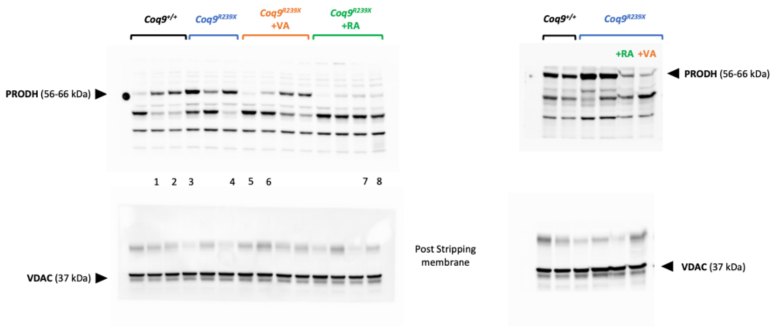

All membranes were cut before the incubation with the primary antibody.

Note: lines 1, 2, 3, 4, 5, 6, 7 and 8 are represented in Figure S8B in supplemental material.

Figure S9. DMGDH in kidney of wild-type and mutant mice with and without treatment.

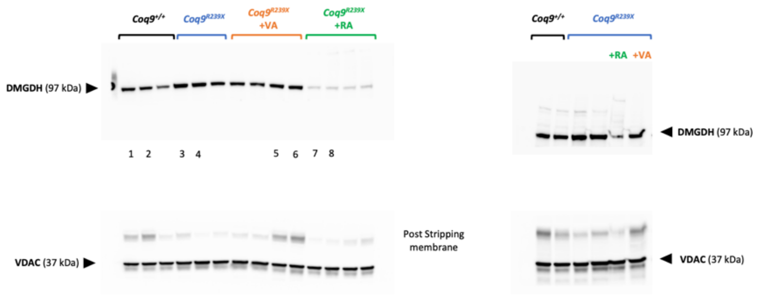

All membranes were cut before the incubation with the primary antibody.

Note: lines 1, 2, 3, 4, 5, 6, 7 and 8 are represented in Figure S8C in supplemental material.

Figure S9. OPA1 in kidney of wild-type and mutant mice with and without treatment.

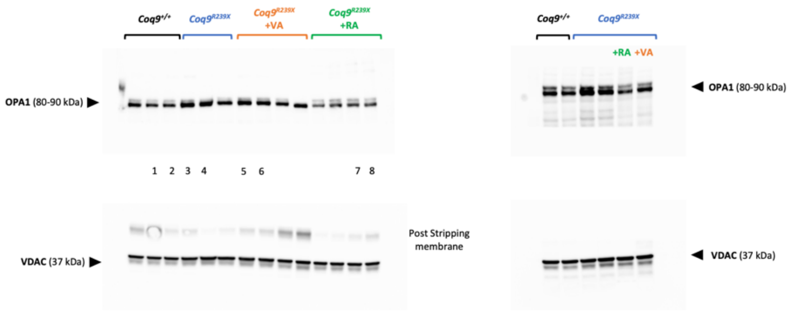

All membranes were cut before the incubation with the primary antibody.

Note: lines 1, 2, 3, 4, 5, 6, 7 and 8 are represented in Figure S8D in supplemental material.

Figure S9. OPA1 in kidney of wild-type and mutant mice with and without treatment.

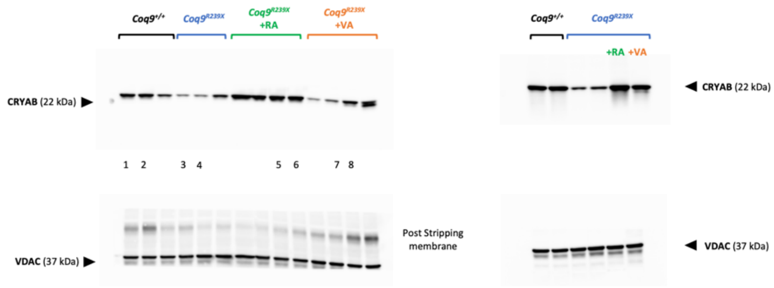

All membranes were cut before the incubation with the primary antibody.

Note: lines 1, 2, 3, 4, 5, 6, 7 and 8 are represented in Figure S8E in supplemental material.

Figure S9. Validation of some key proteins identified in the proteomics analyses.

(**A**) Western blot of PRODH in brain homogenates of *Coq9*$^{+/+}$, *Coq9*^{R239X} mice, *Coq9*^{R239X} mice under 1% β-RA and *Coq9*^{R239X} mice under 1% VA supplementation at 3 months of age.

(**B**-**E**) Western blot of PRODH (**B**), DMGDH (**C**), OPA1 (**D**) and CRYAB (**E**) in kidney homogenates of *Coq9*$^{+/+}$, *Coq9*^{R239X} mice, *Coq9*^{R239X} mice under 1% β-RA and *Coq9*^{R239X} mice under 1% VA supplementation at 3 months of age.

Data are expressed as mean ± SD. *P < 0.05, **P < 0.01, ***P < 0.001, differences *versus* *Coq9*$^{+/+}$; +P < 0.05, ++P < 0.01, +++P < 0.001, *versus* *Coq9*^{R239X}; (one-way ANOVA with a Tukey's *post hoc* test; n = 5 for each group).

(**F**) Original membranes used in this figure.

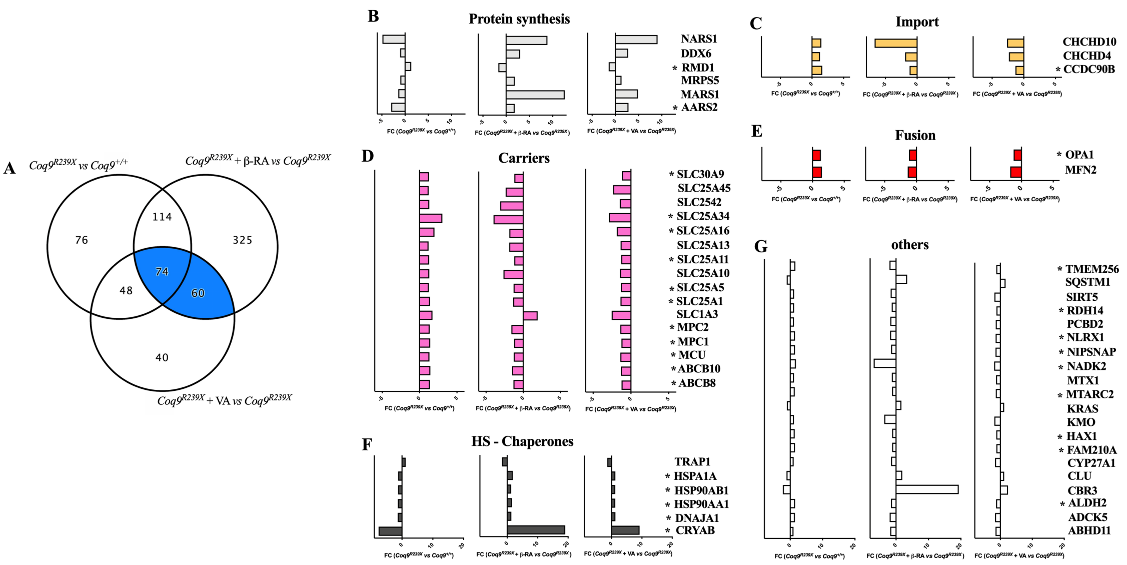

Figure S10. Additional proteomics analysis in the kidneys of *Coq9*^{R239X} mice after 4-HB analogs treatment.

(**A**) Global differences in the protein levels between experimental groups. In blue are marked the 74 proteins altered by the mutation and by β-RA and VA treatments and the 60 proteins modified by β-RA and VA treatments.

(**B-H**) Fold change of the proteins modified by the mutation and normalized by β-RA and VA treatments in the renal mitochondrial proteome in the kidney of *Coq9*$^{+/+}$ mice, *Coq9*^{R239X} mice, *Coq9*^{R239X} mice after 1% β-RA treatment and *Coq9*^{R239X} mice after 1% VA treatment at 3 months of age. Proteins are classified according to its function in protein synthesis (**B**), import (**C**), carriers (**D**), fusion (**E**), chaperones (**F**) and others (**G**). $^*p < 0.05$. Mitochondrial proteomics was performed in isolated mitochondria. *Coq9*$^{+/+}$, n = 5; *Coq9*^{R239X}, n = 5; *Coq9*^{R239X} after β-RA treatment, n = 6; *Coq9*^{R239X} after VA treatment, n = 5.

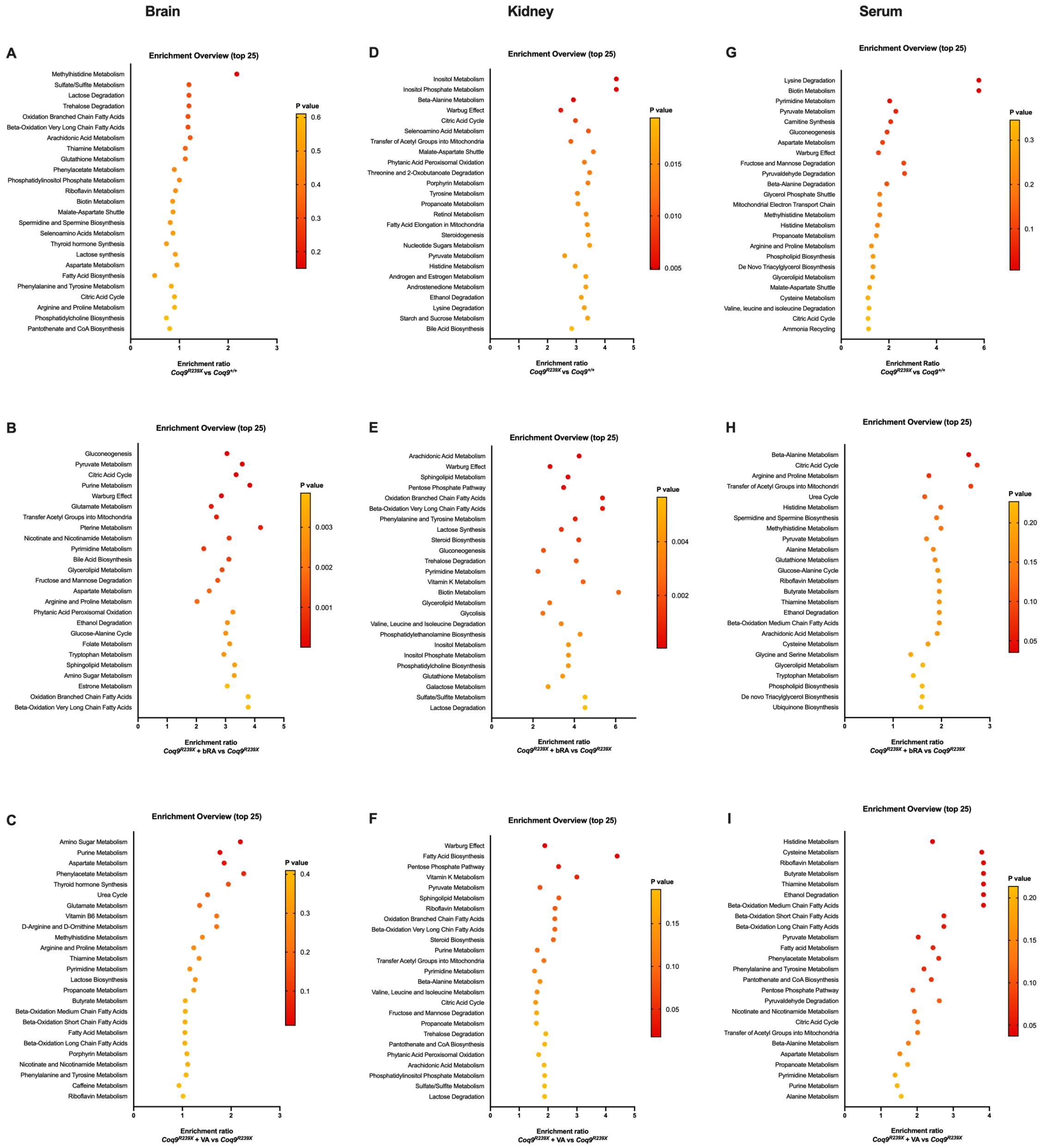

Figure S11. Metabolomics enrichment analysis in the mutant mice.

(A-C) Enrichment analysis of brain samples in the comparison *Coq9*^{R239X} vs *Coq9*$^{+/+}$ (**A**), *Coq9*^{R239X} + β-RA *vs* *Coq9*^{R239X} (**B**) and *Coq9*^{R239X} + VA *vs* *Coq9*^{R239X} (**C**).

(D-F) Enrichment analysis of kidney samples in the comparison *Coq9*^{R239X} vs *Coq9*$^{+/+}$ (**D**), *Coq9*^{R239X} + β-RA *vs* *Coq9*^{R239X} (**E**) and *Coq9*^{R239X} + VA *vs* *Coq9*^{R239X} (**F**).

(G-I) Enrichment analysis of serum samples in the comparison *Coq9*^{R239X} vs *Coq9*$^{+/+}$ (**G**), *Coq9*^{R239X} + β-RA *vs* *Coq9*^{R239X} (**H**) and *Coq9*^{R239X} + VA *vs* *Coq9*^{R239X} (**I**).

Coq9$^{+/+}$, n = 5; *Coq9*^{R239X}, n = 5; *Coq9*^{R239X} after β-RA treatment, n = 5; *Coq9*^{R239X} after VA treatment, n = 5.

Movie S1. Video that shows the difference between a *Coq9*^{R239X} mouse and a *Coq9*^{R239X} mouse after VA treatment, both males at 3 months of age.
